# Supplementary material for: Enzymatic β-elimination in natural product O- and C-glycoside deglycosylation
Source: Nat Commun. 2023 Nov 6;14:7123. doi: 10.1038/s41467-023-42750-0 (PMC10628242; doi:10.1038/s41467-023-42750-0)
Supplement: Supplementary file 1 — Supplementary Information [file 41467_2023_42750_MOESM1_ESM.pdf]

Supplementary Information

Enzymatic β-elimination in natural product *O*- and *C*-glycoside deglycosylation

Johannes Bitter[1], Martin Pfeiffer[1], Annika J. E. Borg[1, 2], Kirill Kuhlmann[3], Tea Pavkov-Keller[3, 4, 5], Pedro A. Sánchez-Murcia[6] and Bernd Nidetzky [1, 2]*

[1] Institute of Biotechnology and Biochemical Engineering, Graz University of Technology, NAWI Graz, Petersgasse 12, A-8010 Graz, Austria

[2] Austrian Centre of Industrial Biotechnology, Krenngasse 37, A-8010 Graz, Austria

[3] Institute of Molecular Biosciences, University of Graz, Humboldtstraße 50/III, A-8010 Graz, Austria

[4] BioTechMed-Graz, Mozartgasse 12/II, A-8010 Graz, Austria

[5] BioHealth Field of Excellence, University of Graz, Humboldtstraße 50, A-8010 Graz, Austria

[6] Laboratory of Computer-Aided Molecular Design, Division of Medicinal Chemistry, Otto-Loewi Research Center, Medical University of Graz, Neue Stiftingstalstraße 6/III, A-8010 Graz, Austria

* Corresponding author; e-mail: bernd.nidetzky@tugraz.at; phone: +43 3168738400

Supplementary Figure 1. General scheme of enzymatic hydrolysis. 5
Supplementary Figure 2. Multistep reaction mechanism of enzymes of glycoside hydrolase family GH4 used in the cleavage of 6-phospho-β-glucoside substrates. 6
Supplementary Figure 3. Puerarin degradation pathway in the human gut bacterium PUE... 7
Supplementary Figure 4. Reaction mechanism of CGE-catalyzed β-elimination as proposed by Mori *et al.* 7
Supplementary Figure 5. *O*- and *C*-glycoside substrates of GlycDH. 8
Supplementary Figure 6. HPLC analysis of GlycDH reaction with different glucosides....... 9
Supplementary Figure 7. *C*-glycosides oxidized by GlycDH. 10
Supplementary Figure 8. TLC analysis of GlycDH-catalyzed oxidations. 11
Supplementary Figure 9. GlycDH catalyzed oxidation of nothofagin. 12
Supplementary Figure 10. ^{1}H NMR spectrum of 3-keto-phlorizin and 3-diol-phlorizin 13
Supplementary Figure 11. COSY analysis of 3-keto-phlorizin and 3-diol-phlorizin. 14
Supplementary Figure 12. ^{1}H NMR (with solvent suppression) spectrum of keto-puerarin 15
Supplementary Figure 13. HSQC analysis of 2-keto-puerarin.. 16
Supplementary Figure 14. TOCSY analysis of 2-keto-puerarin. 17
Supplementary Figure 15. ^{1}H NMR spectrum of 2-keto-nothofagin. 18
Supplementary Figure 16. COSY analysis of 2-keto-nothofagin... 19
Supplementary Figure 17. HPLC traces from GlycDH-catalyzed reactions........................ 20
Supplementary Figure 18. Comparison of operons involving genes homologous to the GlycDH gene of *Rhizobium sp. GIN611*. 21
Supplementary Figure 19. Proposed levoglucosan degradation pathway. 22
Supplementary Figure 20. HPLC chromatogram of an *At*OGE catalyzed deglycosylation of 3-keto-phlorizin. 23
Supplementary Figure 21. TLC analysis of *At*OGE and *At*HYD. 24
Supplementary Figure 22. TLC analysis of 3-keto-phlorizin conversion by enzymes of the glucoside degrading operon of *A. tumefaciens*.. 25
Supplementary Figure 23. *At*OGE 2.0 Å crystal structure. 26
Supplementary Figure 24. Graphical representation of *Pu*CGE subunit α and *At*OGE secondary structures. 27
Supplementary Figure 25. Superposition of *At*OGE subunit α and subunit β. 28
Supplementary Figure 26. Superposition of *At*OGE and CGEs.. 29
Supplementary Figure 27. *At*OGE variants catalyzing 3-keto-phlorizin elimination. 30
Supplementary Figure 28. Overlay of *At*OGE crystal structure and structure with modelled loops. 31
Supplementary Figure 29. RMSD values for enzyme and substrate during MD simulation.32
Supplementary Figure 30. Ring pucker analysis. 33
Supplementary Figure 31. MD-refined binding mode of 3-keto-phlorizin:*At*OGE. 34
Supplementary Figure 32. Catalytically relevant interatomic distances in the active site of *At*OGE in complex with 3-keto-phlorizin. 35
Supplementary Figure 33. MD-refined binding mode of 3-keto-phlorizin:*Pu*CGE. 36
Supplementary Figure 34. Catalytically relevant interatomic distances in the active site of *Pu*CGE in complex with 3-keto-phlorizin. 37
Supplementary Figure 35. MD-refined binding mode of 3-keto-puerarin:*Pu*CGE 38

Supplementary Figure 36. Catalytically relevant interatomic distances in the active site of *Pu*CGE in complex with 3-keto-puerarin. 39
Supplementary Figure 37. *Pu*CGE-catalyzed elimination of keto-*C*-glycosides. 40
Supplementary Figure 38. Time courses of *At*OGE-catalyzed elimination of oxidized *C*-glycoside and oxidized/unoxidized *O*-glycoside substrates. 41
Supplementary Figure 39. Kinetic analysis of OGE- and CGE-catalyzed elimination of 3-keto-phlorizin. 42
Supplementary Figure 40. Relative activity of *Pu*CGE- and *At*OGE in deglycosylation reactions of *O*- and *C*-glycosides in metal-chelating conditions. 43
Supplementary Figure 41. ^{1}H NMR spectrum of products from *At*OGE reaction with 3-keto-phlorizin. 44
Supplementary Figure 42. TOCSY analysis from 2-hydroxy-3-keto-glucal product of *At*OGE-catalyzed 3-keto-phlorizin deglycosylation. 45
Supplementary Figure 43. COSY analysis of products from *At*OGE reaction with 3-keto-phlorizin. 46
Supplementary Figure 44. In situ ^{1}H NMR analysis of *At*OGE reaction with 3-keto-phlorizin. 47
Supplementary Figure 45. ^{1}H NMR spectrum of product from *Pu*CGE reaction with 2-/3-keto-puerarin. 48
Supplementary Figure 46. ^{1}H NMR spectrum of products from *Pu*CGE reaction with 3-keto-phlorizin. 49
Supplementary Figure 47. ^{1}H NMR spectrum of 4NPα3ketoG. 50
Supplementary Figure 48. ^{1}H NMR spectrum of 4NPβ3ketoG. 51
Supplementary Figure 49. Product identification from the GlycDH-catalyzed reaction with maltose. 52
Supplementary Figure 50. Product identification from the GlycDH-catalyzed reaction with cellobiose. 53
Supplementary Figure 51. Stereochemical preference of *At*OGE and *Pu*CGE. 54
Supplementary Figure 52. ^{1}H NMR spectra of *At*OGE-catalyzed elimination of 4NPα3ketoG and 4NPβ3ketoG. 55
Supplementary Figure 53. ^{1}H NMR spectra of *At*OGE-catalyzed elimination of 3'-ketomaltose and 3'-keto-cellobiose. 56
Supplementary Figure 54. Spontaneous deglycosylation of 3-keto-phlorizin. 57
Supplementary Figure 55. Conserved Tyr residues among CGEs. 58
Supplementary Figure 56. Sequence alignment of CGEs. 59
Supplementary Figure 57. Relative activity of *Pu*CGE variants with *O*- and *C*-glycosides compared to the wild-type (WT). 60
Supplementary Figure 58. BglK catalyzed phosphorylation of phlorizin and nothofagin. 61
Supplementary Figure 59. TLC analysis of BglK-catalyzed phosphorylation. 62
Supplementary Figure 60. Time courses of GlvA-catalyzed reactions. 63
Supplementary Figure 61. Time courses of BglT-catalyzed reactions. 64
Supplementary Figure 62. TLC analysis of GlvA-catalyzed deglycosylation of phlorizin6P and nothofagin6P. 65

Supplementary Figure 63. HPLC chromatogram of GlvA-catalyzed deglycosylation of phlorizin6P. 66
Supplementary Figure 64. Proposed mechanisms of glycosyltransferase and alkyltransferase reactions. 67
Supplementary Figure 65. SDS-PAGE showing the purified enzymes used in this study. . 68

Supplementary Table 1. Activity parameters of GlycDH. 69
Supplementary Table 2. Crystallographic data of *At*OGE. 70
Supplementary Table 3. RMSD values of *At*OGE monomer β superimposed with monomer α and CGEs. 71
Supplementary Table 4. Activity parameters of deglycosylating enzyme systems compared. 72
Supplementary Table 5. Activity of *Pu*CGE variants with *O*- and *C*-glycosides compared to the wild-type form. 73
Supplementary Table 6. Sequences of enzymes. 74
Supplementary Table 7. Oligonucleotide primers used in this study. 78

Supplementary Figures

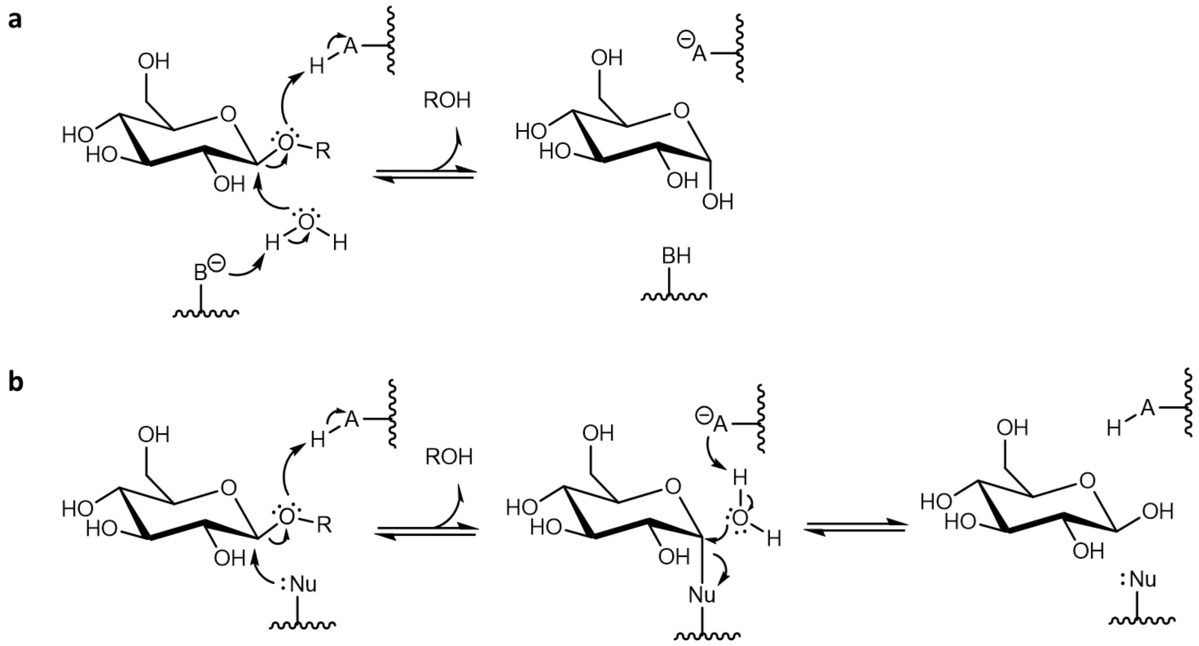

Supplementary Figure 1. General scheme of enzymatic hydrolysis of a β-glucoside by an inverting (**a**) and retaining (**b**) β-glycoside hydrolase. Aglycone shown as R, the enzyme catalytic base and acid as B and A, respectively. Drawn based on literature.[1,2] Nu = nucleophile; A = general acid catalyst; B = general base catalyst.

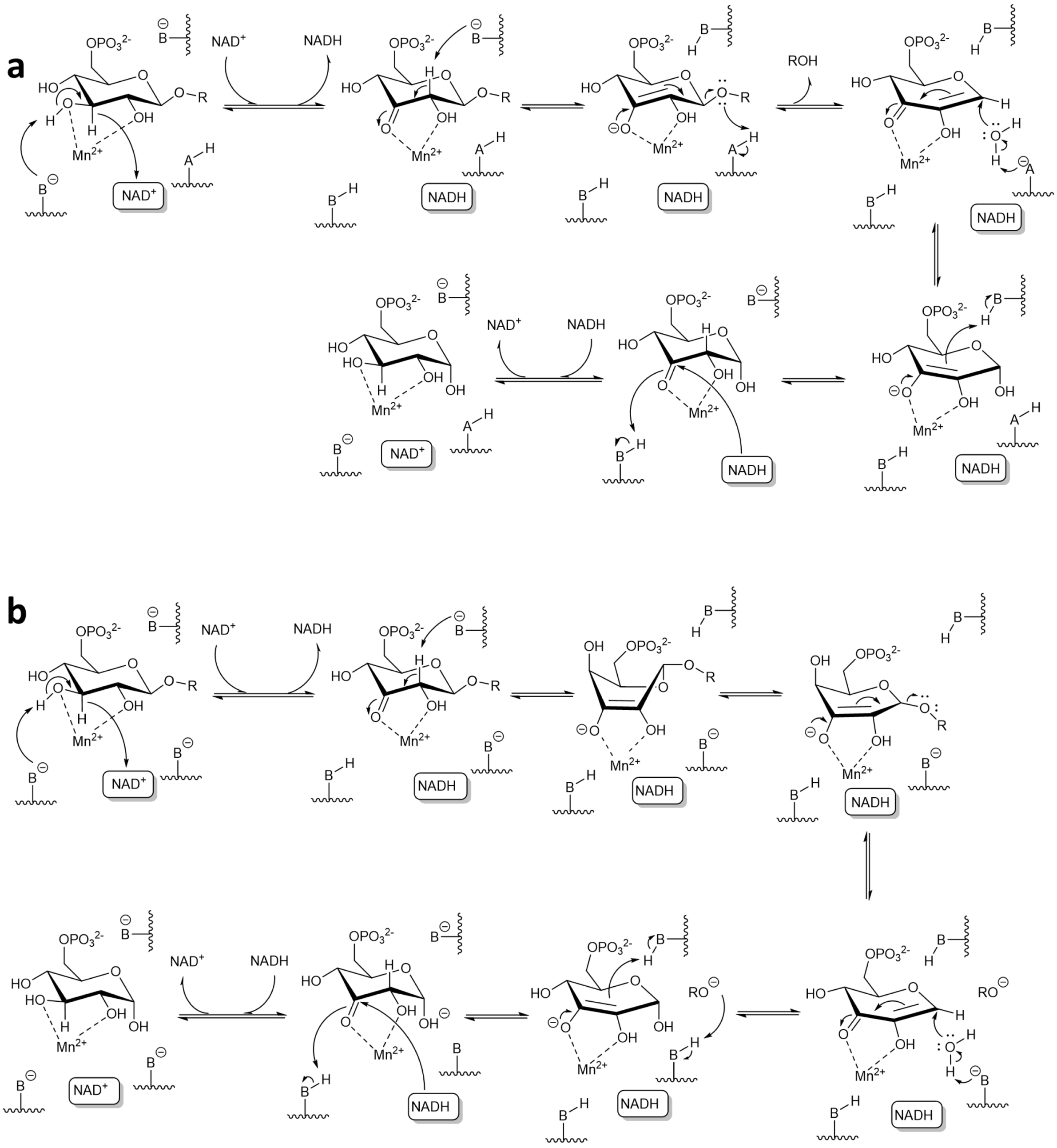

Supplementary Figure 2. Multistep reaction mechanism of enzymes of glycoside hydrolase family GH4 used in the cleavage of 6-phospho-β-glucoside substrates. **a.** Proposed mechanism involving acid-assisted β-elimination using GlvA from *Bacillus subtilis*[3] as an example. The scheme is based on Rajan et al. [4] and Yip et al.[5] **b.** Proposed reaction mechanism for GH4 family enzyme α-galactosidase from *Citrobacter freundii* (MelA) involving sugar ring puckering as a significant rate limiting step and β-elimination independent of acid catalysis. The reaction mechanism is based on Sannikova et al.[6] B = general base catalyst.

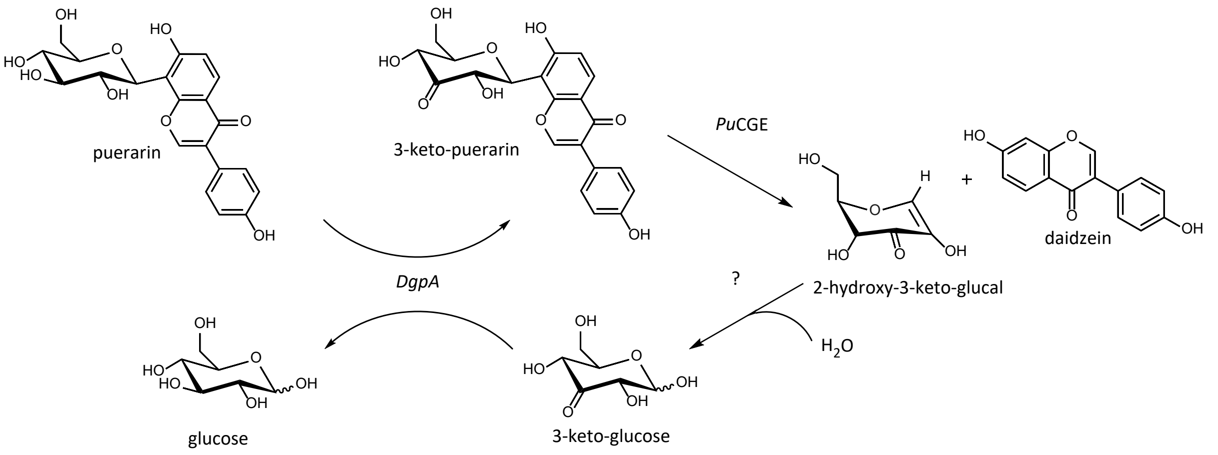

Supplementary Figure 3. Puerarin degradation pathway in the human gut bacterium PUE (*Pu*CGE), as proposed by Nakamura *et al.*[7] The pathway involves the oxidoreductase DgpA and the *C*-glycoside eliminase *Pu*CGE. Puerarin is oxidized by DgpA to 3-keto-puerarin. *Pu*CGE deglycosylates the compound to the respective aglycone daidzein and 2-hydroxy-3-keto-glucal, which is subsequently converted to 3-keto-glucose and then reduced to glucose. The conversion of 3-keto-glucal to 3-keto-glucose involves an enzyme yet to be shown.

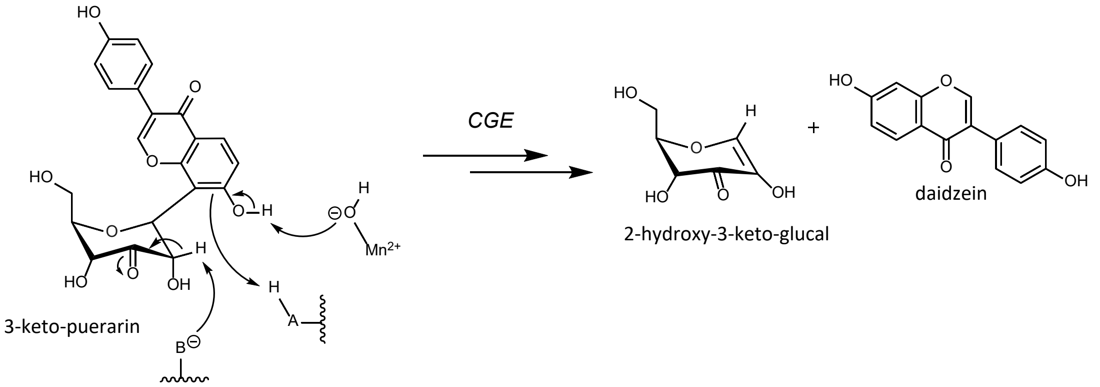

Supplementary Figure 4. Reaction mechanism of CGE-catalyzed β-elimination of 3-keto-puerarin, as proposed by Mori *et al.*[8] A catalytic base promotes abstraction of the C2 hydrogen of the 3-keto-D-glucosyl moiety. The hydroxy group *ortho* to the reactive carbon of the aglycone is deprotonated by metal-bound hydroxide. Proton transfer from a catalytic acid leads to a proposed de-aromatization of the aglycone. Elimination causes cleavage of the C-C linkage and yields 2-hydroxy-3-keto-glucal and daidzein. A = general acid catalyst; B = general base catalyst.

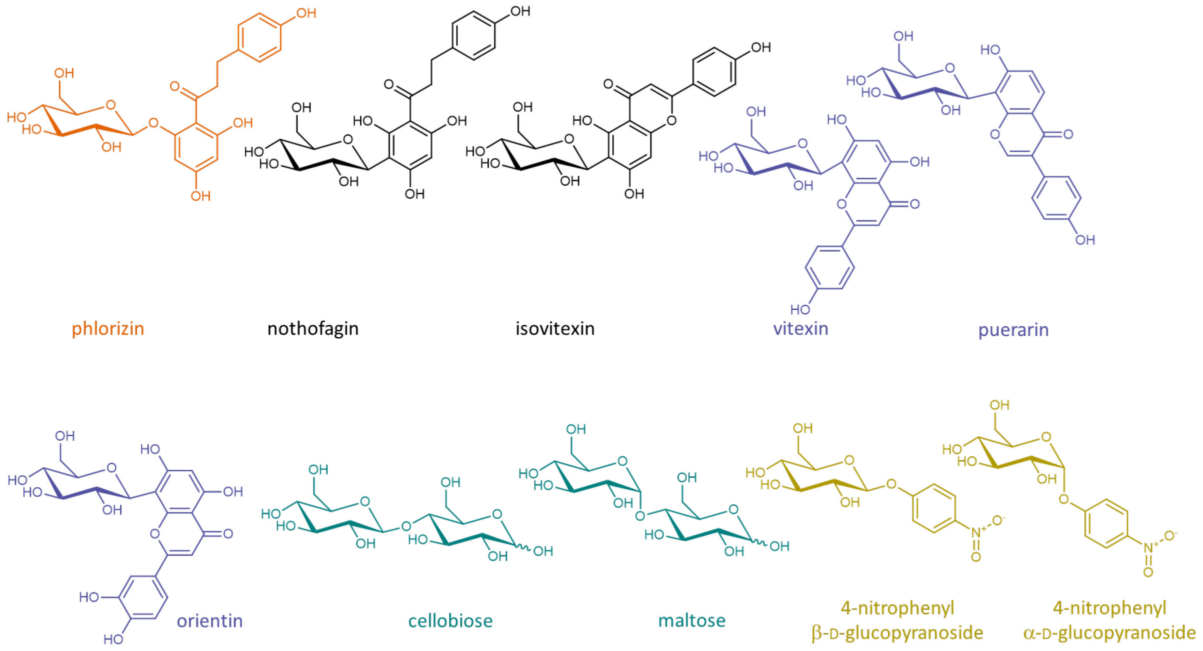

Supplementary Figure 5. *O*- and *C*-glycoside substrates of GlycDH. *O*-glycosides in orange, *C*-6-glycosides in black, *C*-8-glycosides in blue, *O*-linked disaccharides in turquoise and 4-nitrophenyl substrates in ochre.

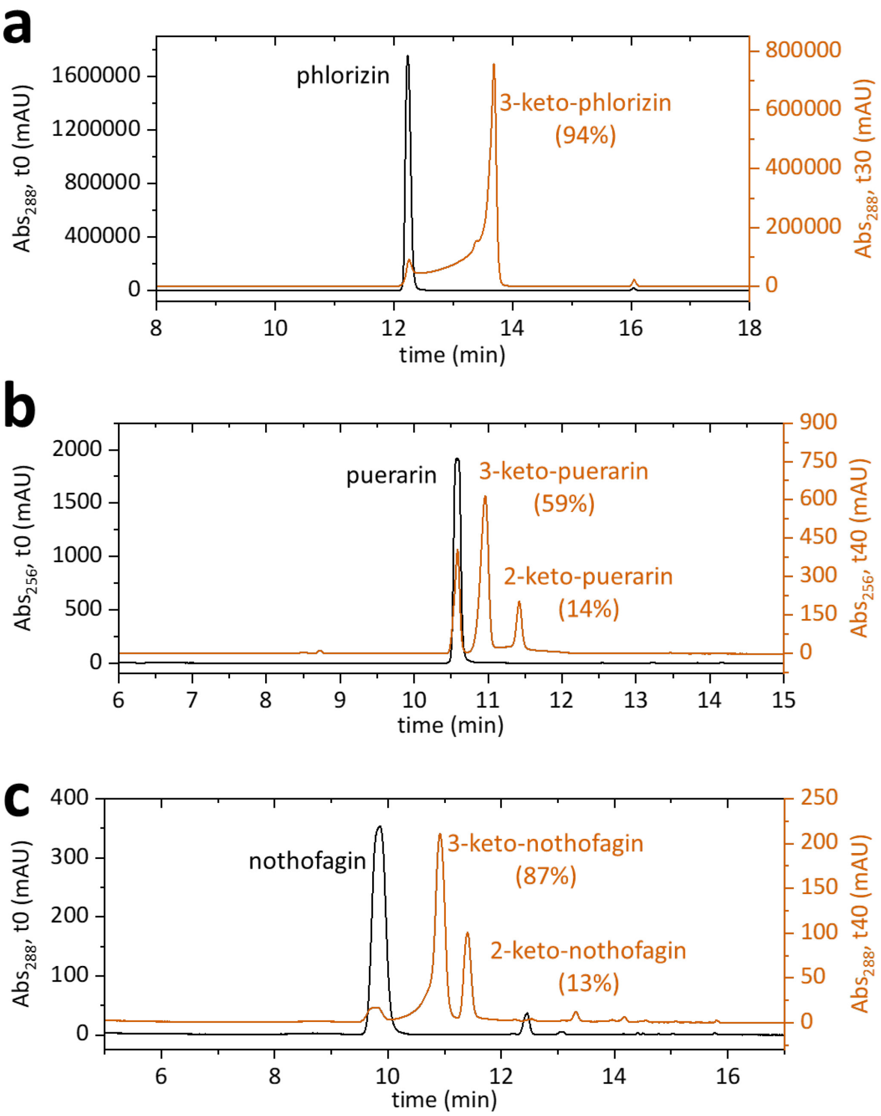

Supplementary Figure 6. HPLC analysis of GlycDH reaction with different glucosides (n = 1 individual experiment). **a.** Phlorizin. **b.** Puerarin. **c.** Nothofagin. Reactions **a**, **b** and **c** were performed in potassium phosphate buffer (100 mM; pH 5.7) with 0.02, 0.25 and 0.10 mg/mL enzyme, respectively. For puerarin, 2.0 mM TCEP was added. All reactions were supplied with 8.0 mM $K_3[Fe(CN)_6]$ as electron acceptor and substrates were dissolved in 10% DMSO. Reactions were carried out at 37 °C under agitation (650 rpm), quenched in MeCN and analyzed on HPLC. The 2- and 3-keto products were separated by HPLC and their identity was assigned based NMR analysis (Supplementary Figure 10-16). For further experimental details and for the analytical procedures used, see the Methods section of the main manuscript.

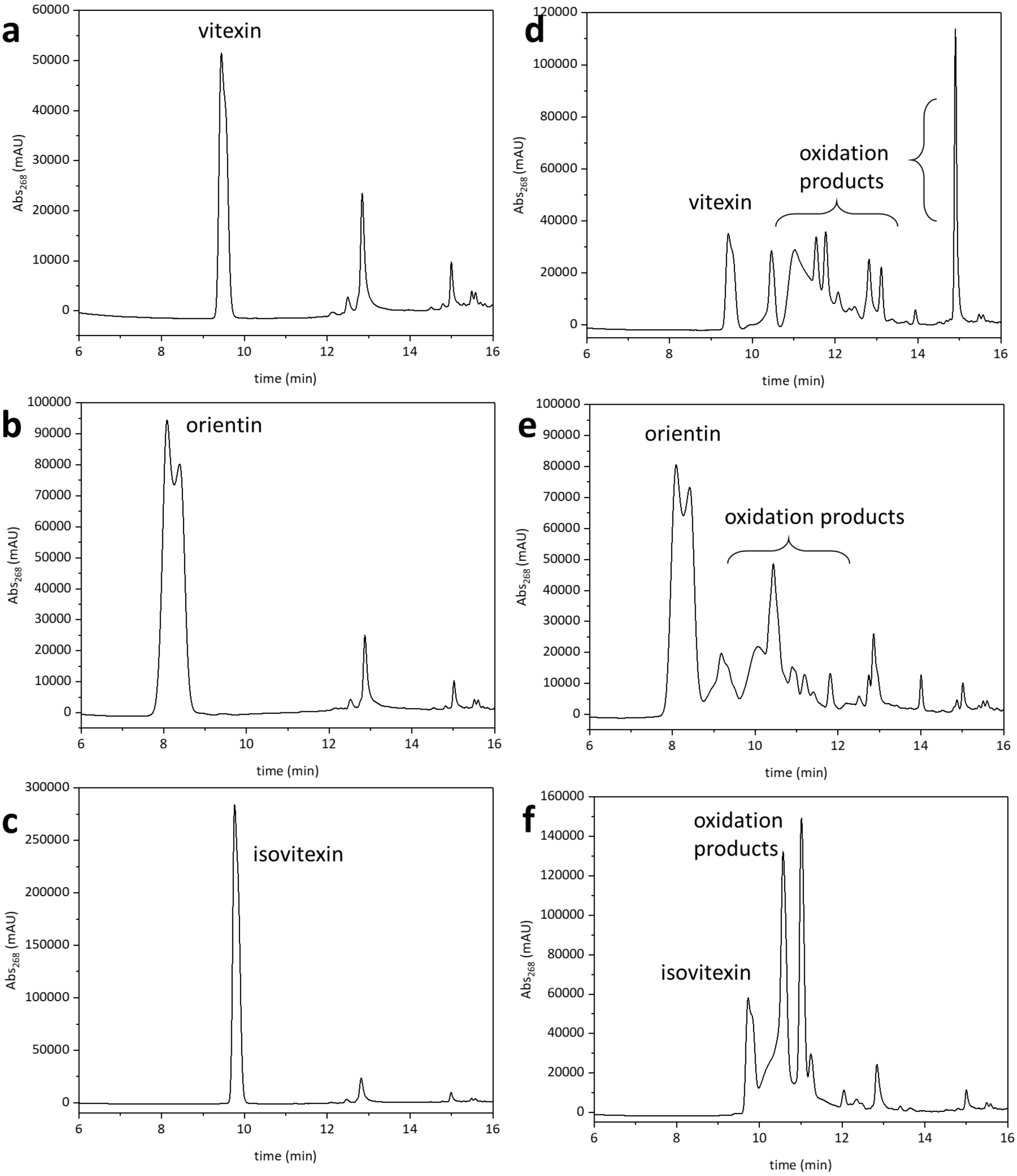

Supplementary Figure 7. *C*-glycosides oxidized by GlycDH (n = 1 individual experiment). **a**, **b** and **c**. Negative controls with no enzyme added; **d**, **e** and **f**. Reactions catalyzed by GlycDH (0.25 mg/mL). Substrates (2.0 mM) were dissolved in potassium phosphate buffer (100 mM; pH 5.7) containing 10% (v/v) DMSO, 8.0 mM $K_3[Fe(CN)_6]$. Reactions were carried out at 37 °C and 650 rpm agitation. Samples were quenched with MeCN and measured on HPLC.

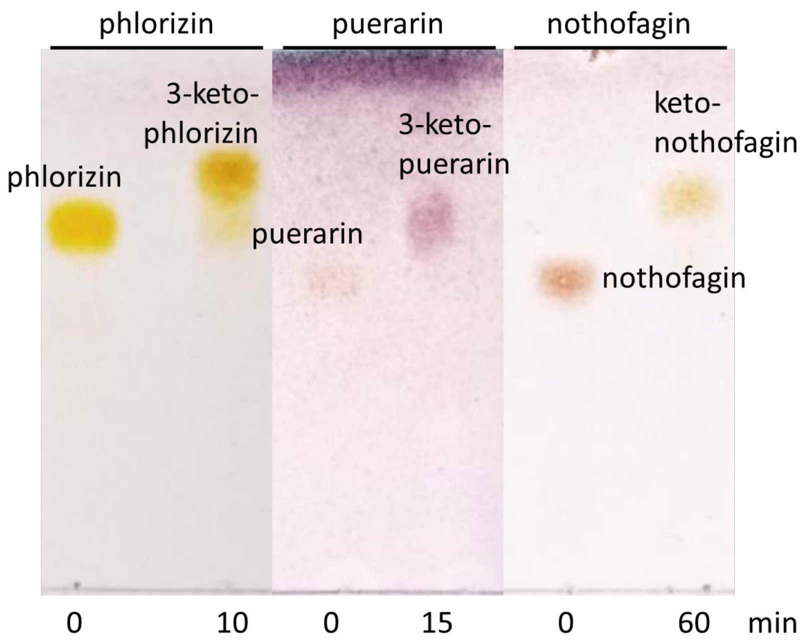

Supplementary Figure 8. TLC analysis of GlycDH-catalyzed oxidation of phlorizin, puerarin and nothofagin (n = 1 individual experiment). Phlorizin, puerarin and nothofagin oxidations were performed in potassium phosphate buffer (100 mM; pH 5.7) with 0.02, 0.25 and 0.10 mg/mL enzyme, respectively. For puerarin, 2.0 mM TCEP were added. All reactions were supplied with 8.0 mM $K_3[Fe(CN)_6]$ as electron acceptor and substrates were dissolved in 10% (v/v) DMSO. Reactions were performed in 37 °C under agitation (650 rpm). Compounds were separated with a 9:1 mobile phase of MeCN with H_2O and TLC plates were stained with *p*-anisaldehyde based stain. Source data are provided as a Source Data file.

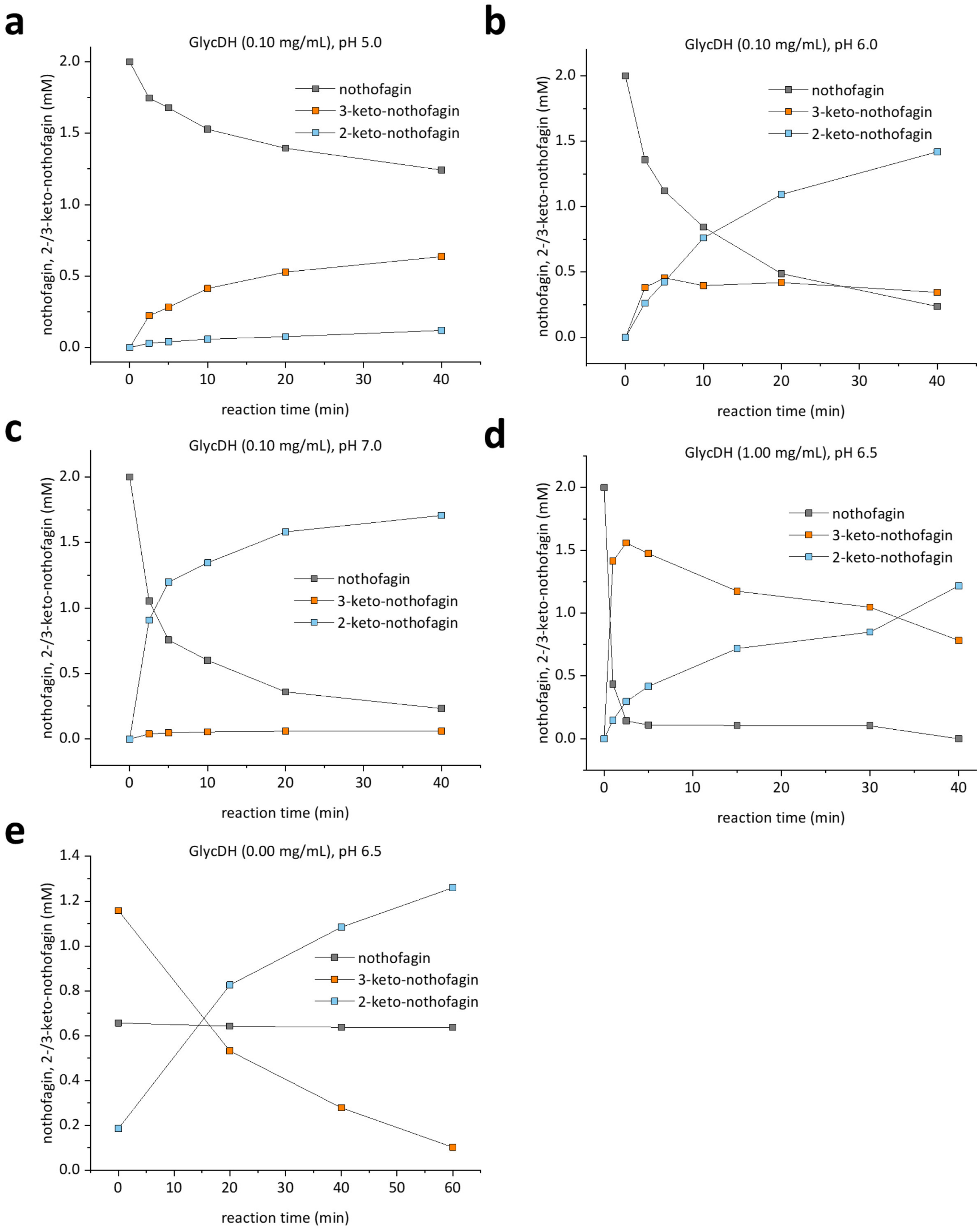

Supplementary Figure 9. GlycDH catalyzed oxidation of nothofagin and spontaneous isomerization of 3-keto- to 2-keto-nothofagin. The effect of pH was analyzed at pH 5.0, 6.0, and 7.0 (**a-c**). The effect of excess GlycDH was studied at a protein loading of 1.00 mg/mL (**d**). The isomerization of isolated 2-/3-keto-nothofagin in the absence of protein is shown in **e**. Reactions are performed at 37 °C and 650 rpm agitation. Standard conditions used were 100 mM potassium phosphate buffer (pH 6.5), 10% (v/v) DMSO, 8.0 mM $K_3[Fe(CN)_6]$, 2.0 mM nothofagin. GlycDH was used at a concentration of 0.10 mg/mL unless mentioned otherwise. For further experimental details and for the analytical procedures used, see the Methods section of the main manuscript. (n = 1 individual experiment). Source data are provided as a Source Data file.

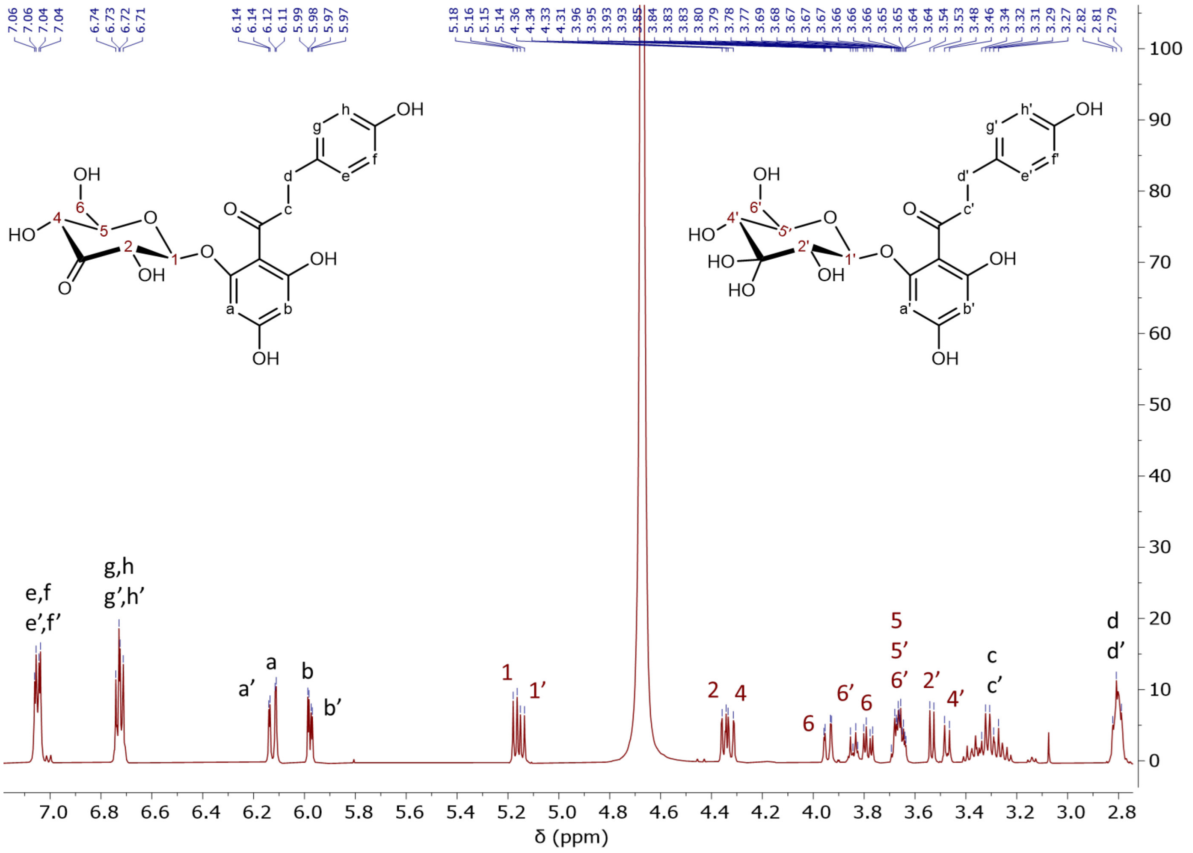

Supplementary Figure 10. ^{1}H NMR spectrum of 3-keto-phlorizin and 3-diol-phlorizin (500 MHz, 10% DMSO-d6, phosphate buffer D_2O pD 7.0): δ 5.17 (d, J = 7.9 Hz, 1H, H-1), 5.14 (d, J = 7.9 Hz, 1H, H-1'), 4.35 (d, J = 7.8 Hz, 1H, H-2), 4.32 (d, J = 10.2 Hz, 1H, H-4), 3.94 (dd, J = 12.5, 2.1 Hz, 1H, H-6), 3.84 (dd, J = 9.8, 4.4 Hz, 1H, H-6'), 3.78 (dd, J = 12.5, 4.9, 1H, H-6), 3.69–3.64 (m, 3H, H-5, H-5' and H.6'), 3.53 (d, J = 7.9, 1H, H-2'), 3.47 (d, J = 9.6, 1H, H-4')

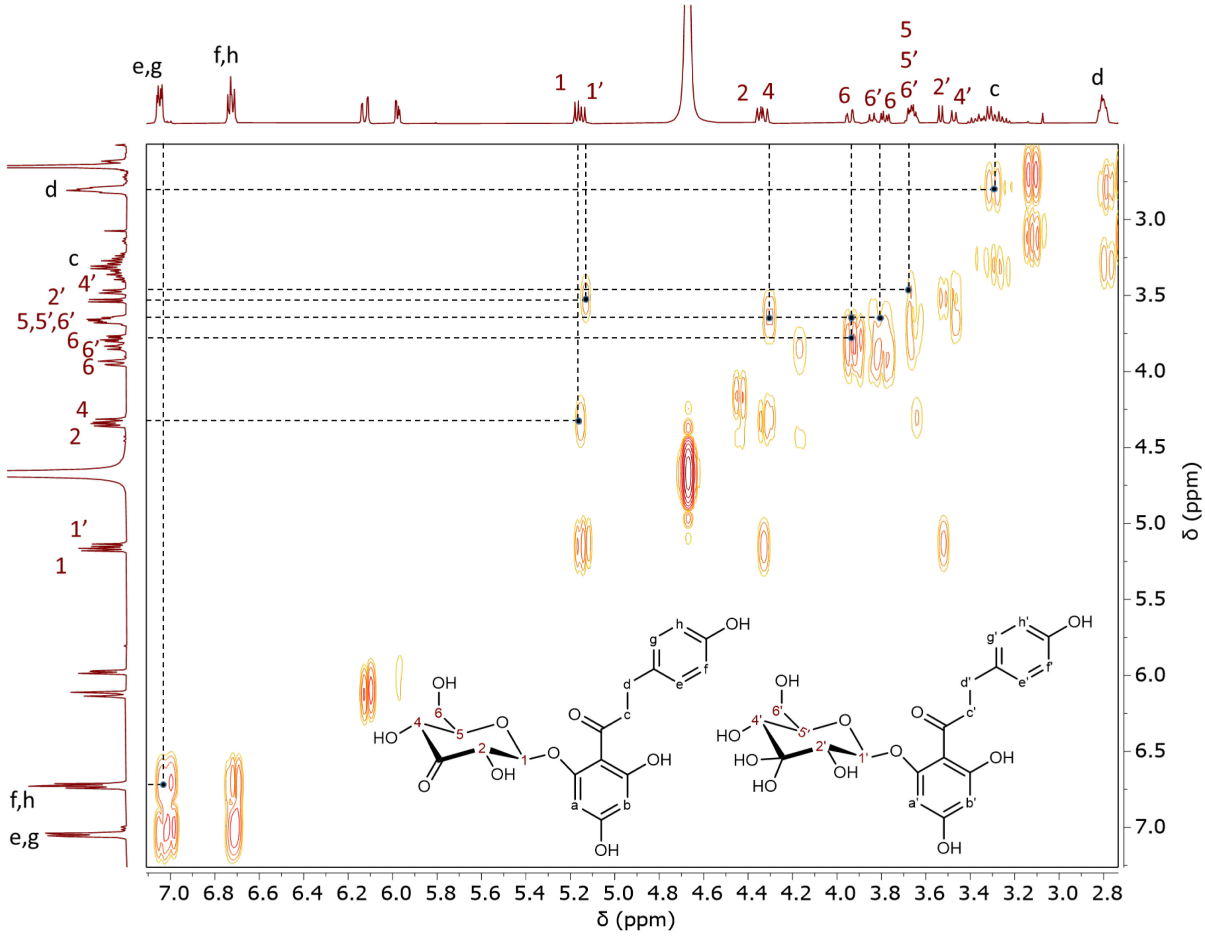

Supplementary Figure 11. Correlated spectroscopy (COSY) analysis of 3-keto-phlorizin and 3-diol-phlorizin (500 MHz, 10% DMSO-d6, phosphate buffer in D_2O, pD 7.0). 2D-NMR shows proton coupling of H-e and H-g with H-f and H-h, H-1 with H-2, H-4 with H-5, H-5 with H-6, H-6 with H-6, H-1′ with H-2′, H-4′ with H-5′, H-5′ with H-6′, H-6′ with H-6′, and H-c with H-d.

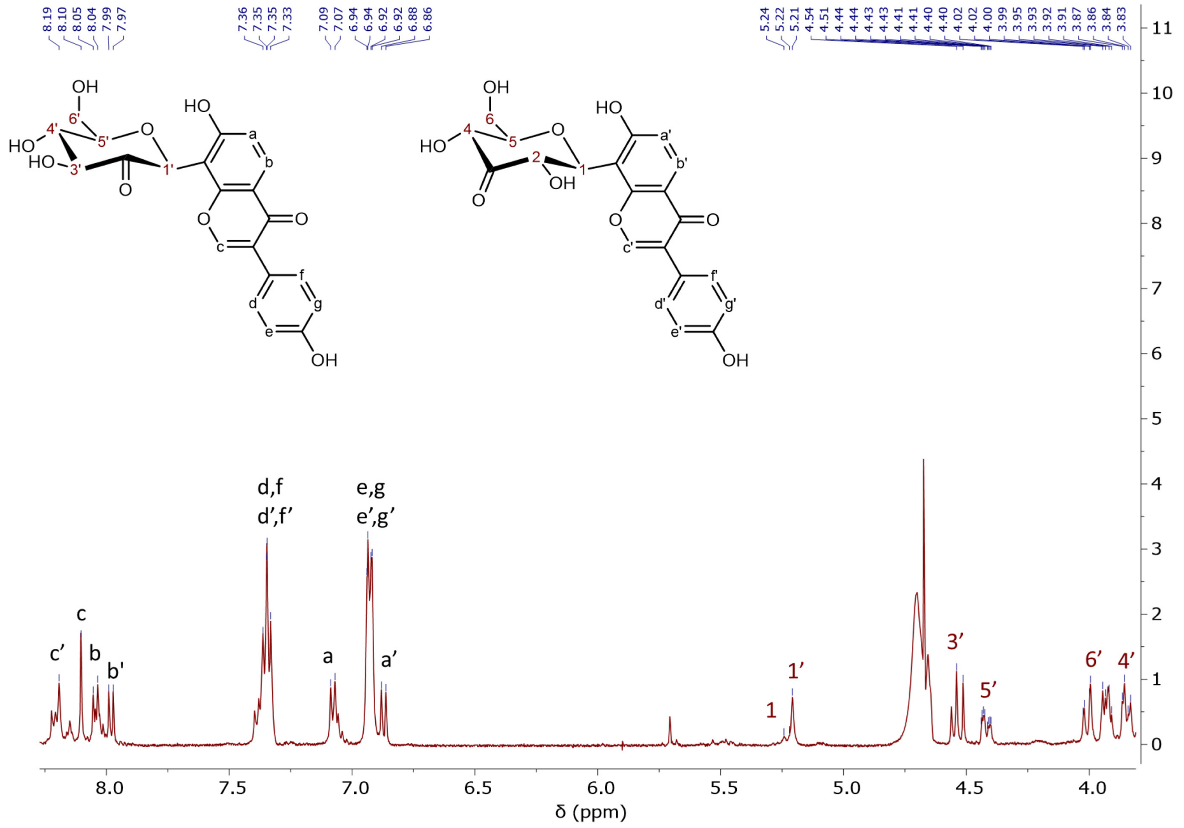

Supplementary Figure 12. ^{1}H NMR (with solvent suppression) spectrum of keto-puerarin (500 MHz, 10% DMSO-d6, D_2O): δ 8.19 (s, 1H, H-c’), 8.10 (s, 1H, H-c), 8.04 (d, J = 9.1 Hz, 1H, H-b), 7.98 (d, J = 9.2 Hz, 1H, H-b’), 7.38–7.31 (m, 4H, H-d, H-f, H-d’ and H-f’), 7.08 (d, J = 8.8 Hz, 1H, H-a), 6.94–6.92 (m, 4H, H-e, H-g, H-e’ and H-g’), 6.87 (d, J = 9.1 Hz, 1H, H-a’), 5.23 (d, J = 11.2 Hz, 1H, H-1), 5.21 (s, 1H, H-1’), 4.53 (d, J = 13.3 Hz, 1H, H-3’), 4.42 (m, J = 12.9, 4.9, 2.2, 1H, H-5’), 4.01 (dd, J = 12.3, 2.3 Hz, 1H, H-6’), 3.93 (dd, J = 12.8, 5.3 Hz, 1H, H-6’), 3.84 (d, J = 12.5 Hz,1H, H-4’)

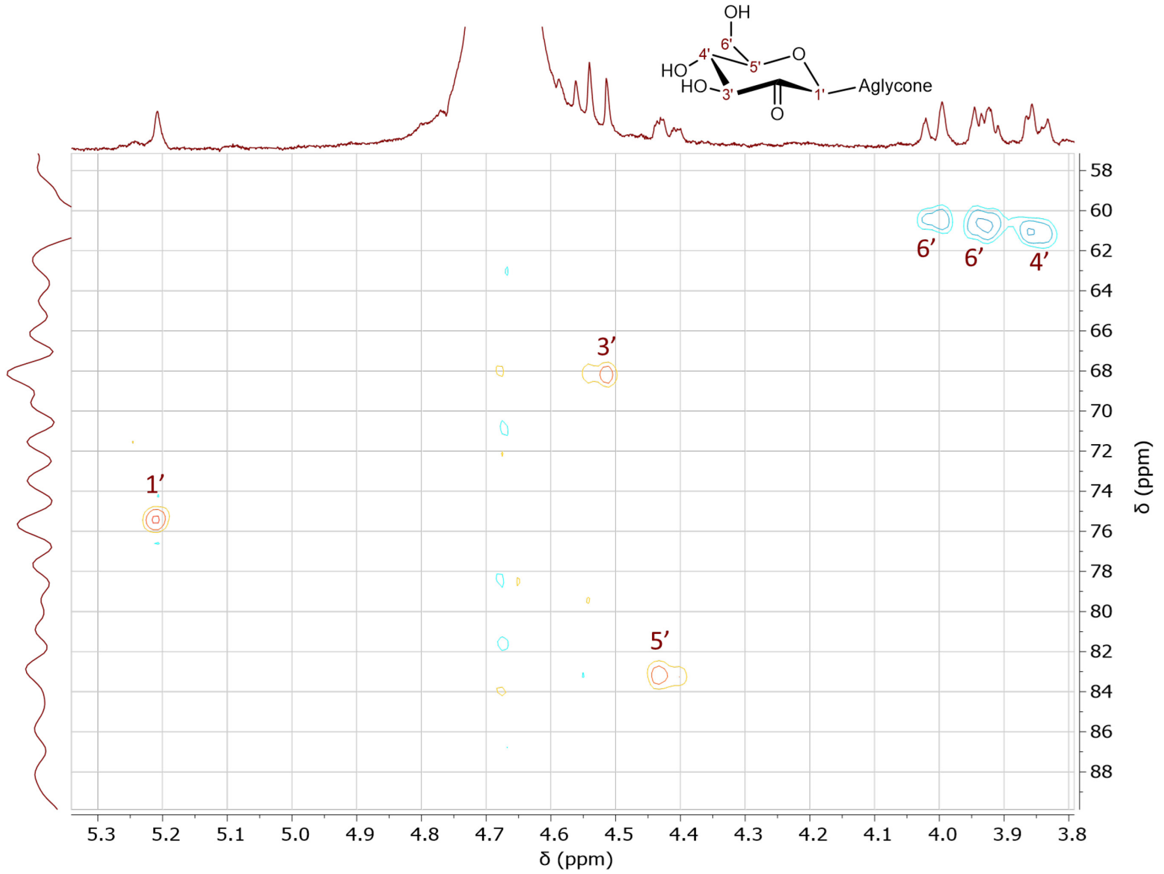

Supplementary Figure 13. Heteronuclear single quantum coherence spectroscopy (HSQC) analysis of 2-keto-puerarin (500 MHz, 10% DMSO-d6, D_2O). Coupling of carbons and protons of sugar moiety (2-keto-β-glucosyl moiety) is shown.

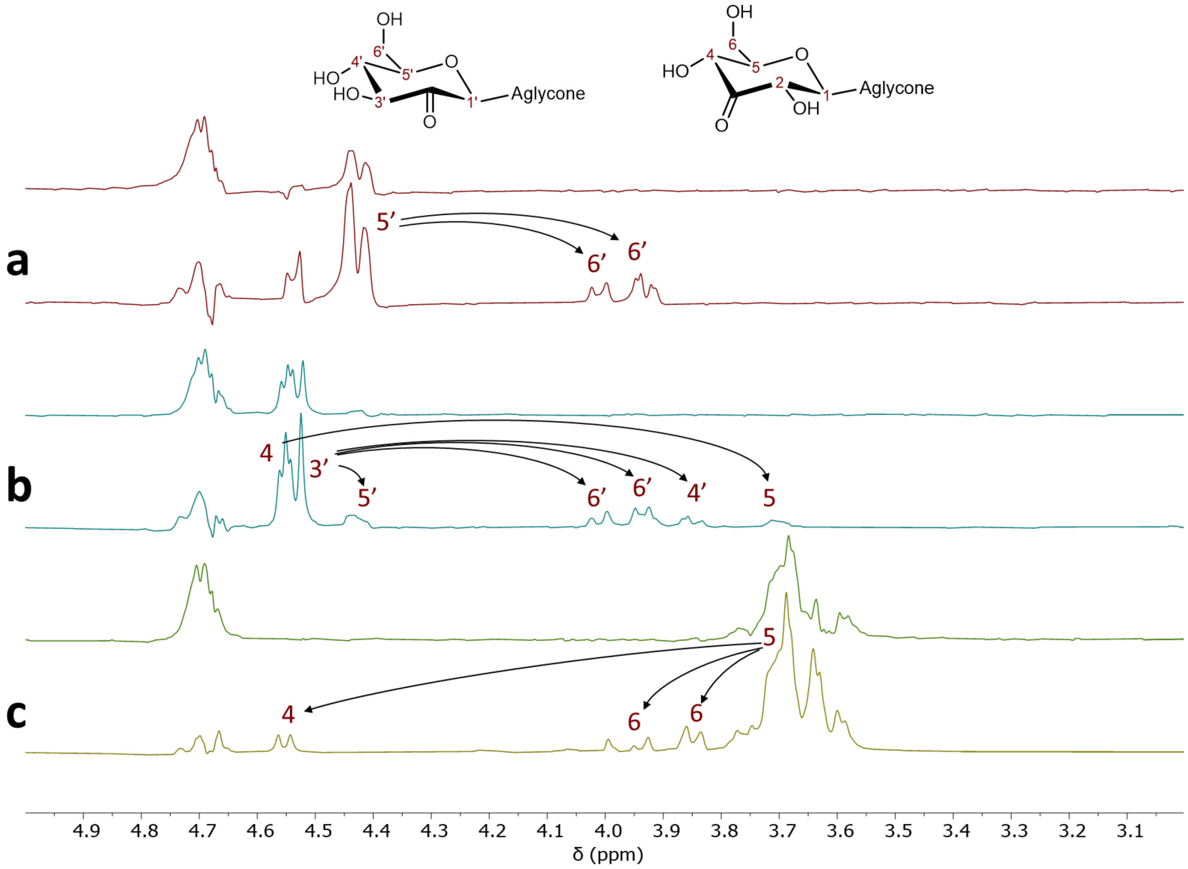

Supplementary Figure 14. Total correlation spectroscopy (TOCSY) analysis of 2-keto-puerarin (500 MHz, 10% DMSO-d6, D_2O). **a.** The maroon upper graph was pulsed at a selective band center of 4.42 ppm and a width of 29.2 Hz. **b.** The turquoise upper graph was pulsed at a selective band center of 4.54 ppm and a width of 34.9 Hz. **c.** The olive upper graph was pulsed at a selective band center of 3.67 ppm and a width of 117 Hz.

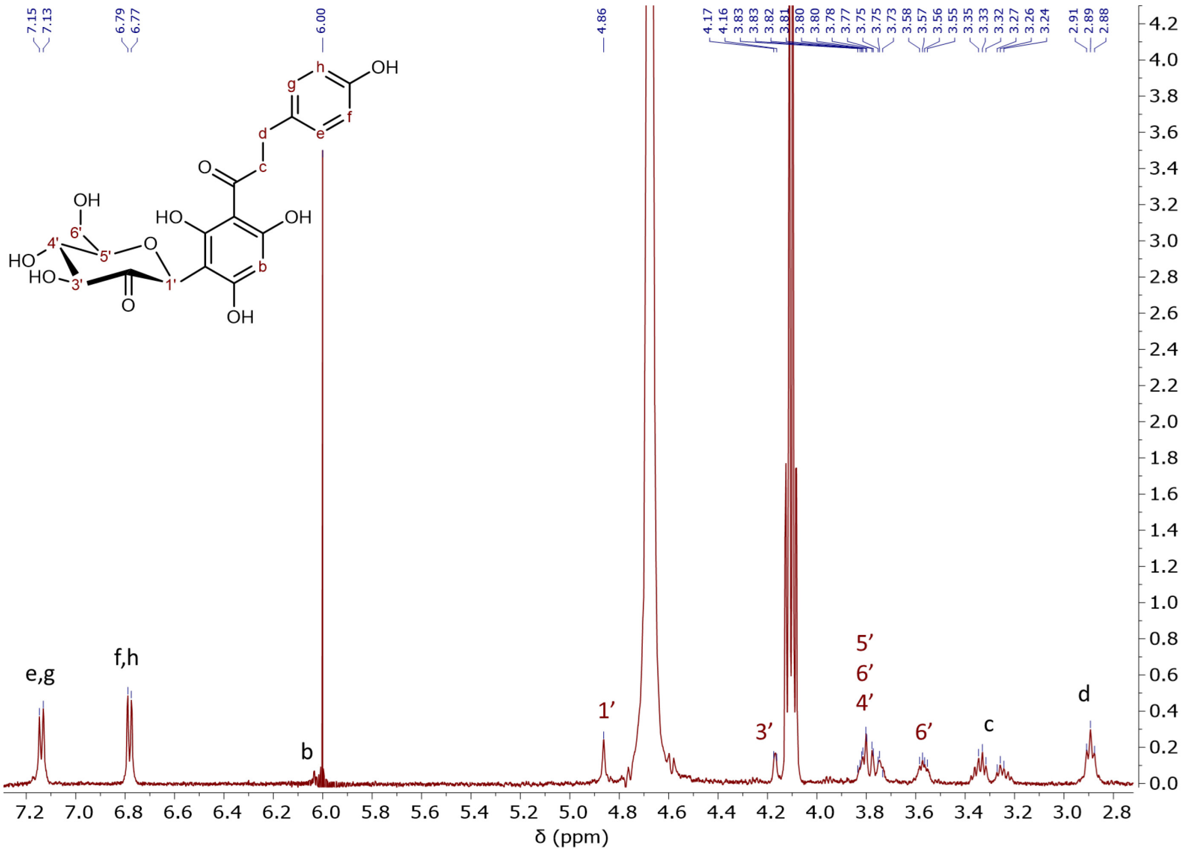

Supplementary Figure 15. ^{1}H NMR spectrum of 2-keto-nothofagin (500 MHz, 10% DMSO-d6, D_2O): δ 7.14 (d, J = 7.8 Hz, 2H, H-e and H-g), 6.78 (d, J = 6.9 Hz, 2H, H-f and H-h), 6.03 (s, 1H, H-b), 4.86 (s, 1H, H-1'), 4.17 (d, J = 5.8 Hz, 1H, H-3'), 3.84–3.81 (m, 1H, H-5'), 3.79 (dd, J = 12.9, 2.1 Hz, 1H, H-6'), 3.74 (t, J = 6.0 Hz, 1H, H-4'), 3.57 (dd, J = 10.0, 5.8 Hz, 1H, H-6'), 3.33 (t, J = 7.6 Hz, 1H, H-c), 3.26 (t, J = 7.6 Hz, 1H, H-c), 2.90 (t, J = 7.8 Hz, 2H, H-d).

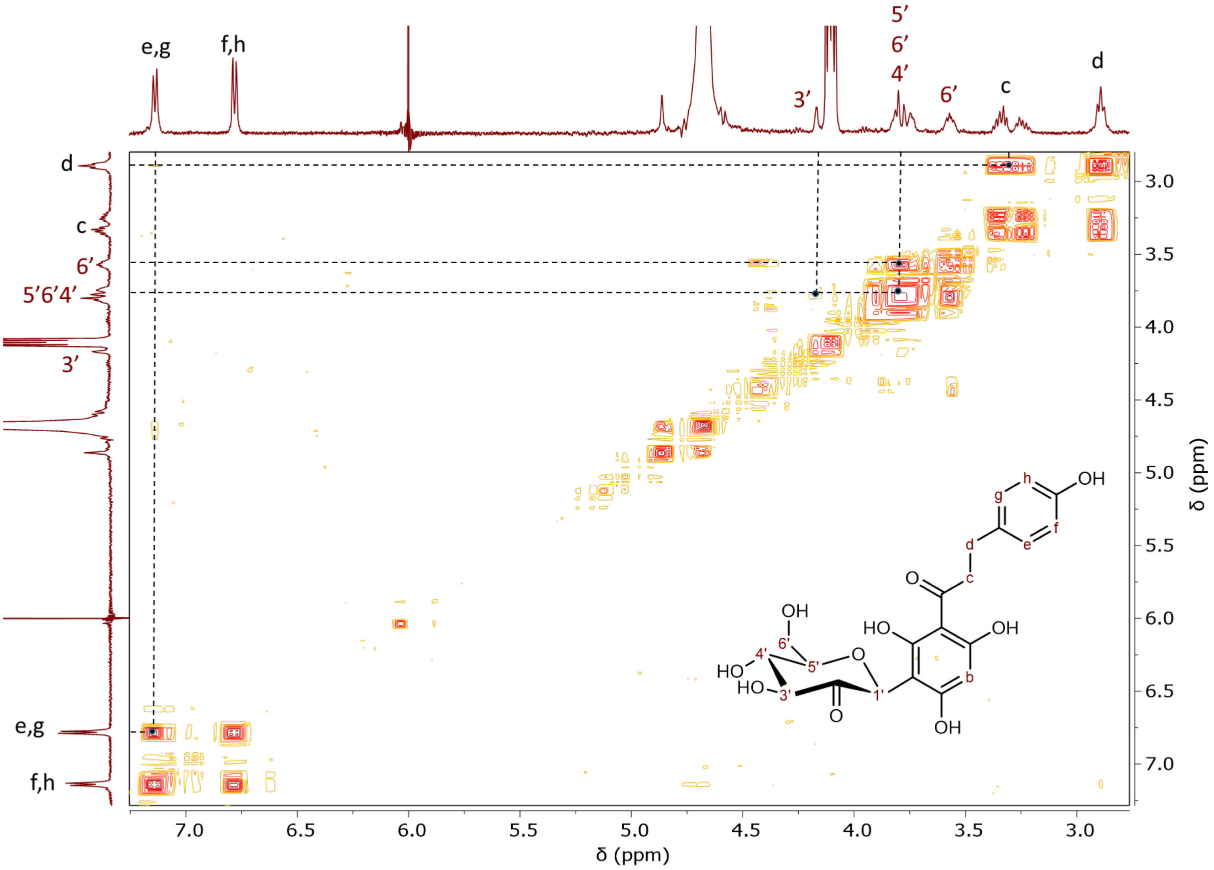

Supplementary Figure 16. COSY analysis of 2-keto-nothofagin (500 MHz, 10% DMSO-d6, D_2O). 2D-NMR shows proton coupling of H-e and H-g with H-f and H-h, H-3’ with H-4’, H-4’ with H-5’, H-5’ with H-6’, H-6’ with H-6’ and H-c with H-d.

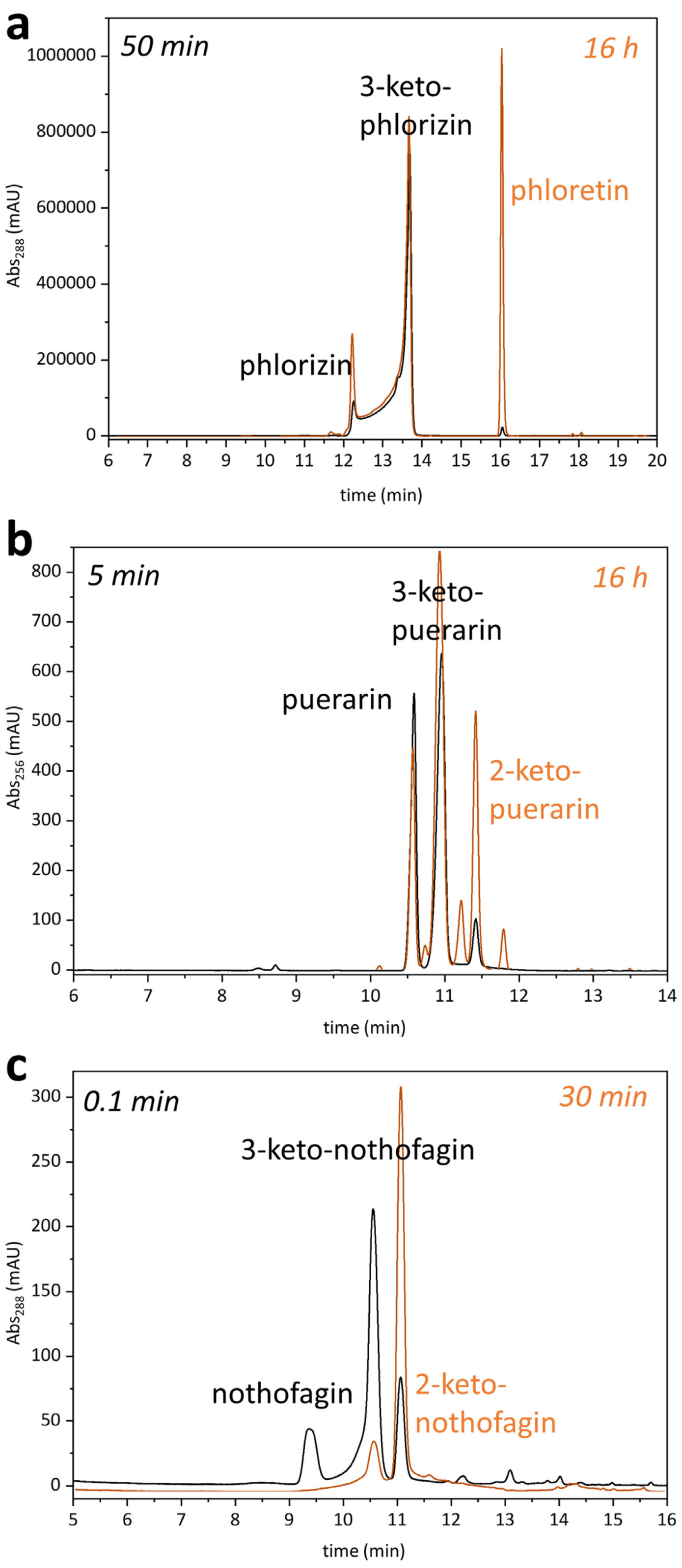

Supplementary Figure 17. HPLC traces from GlycDH-catalyzed reactions and spontaneous isomerization/degradation products (n = 1 individual experiment). **a**, **b** and **c.** Oxidation products from phlorizin, puerarin and nothofagin, respectively, in black and side products upon longer incubation in orange.

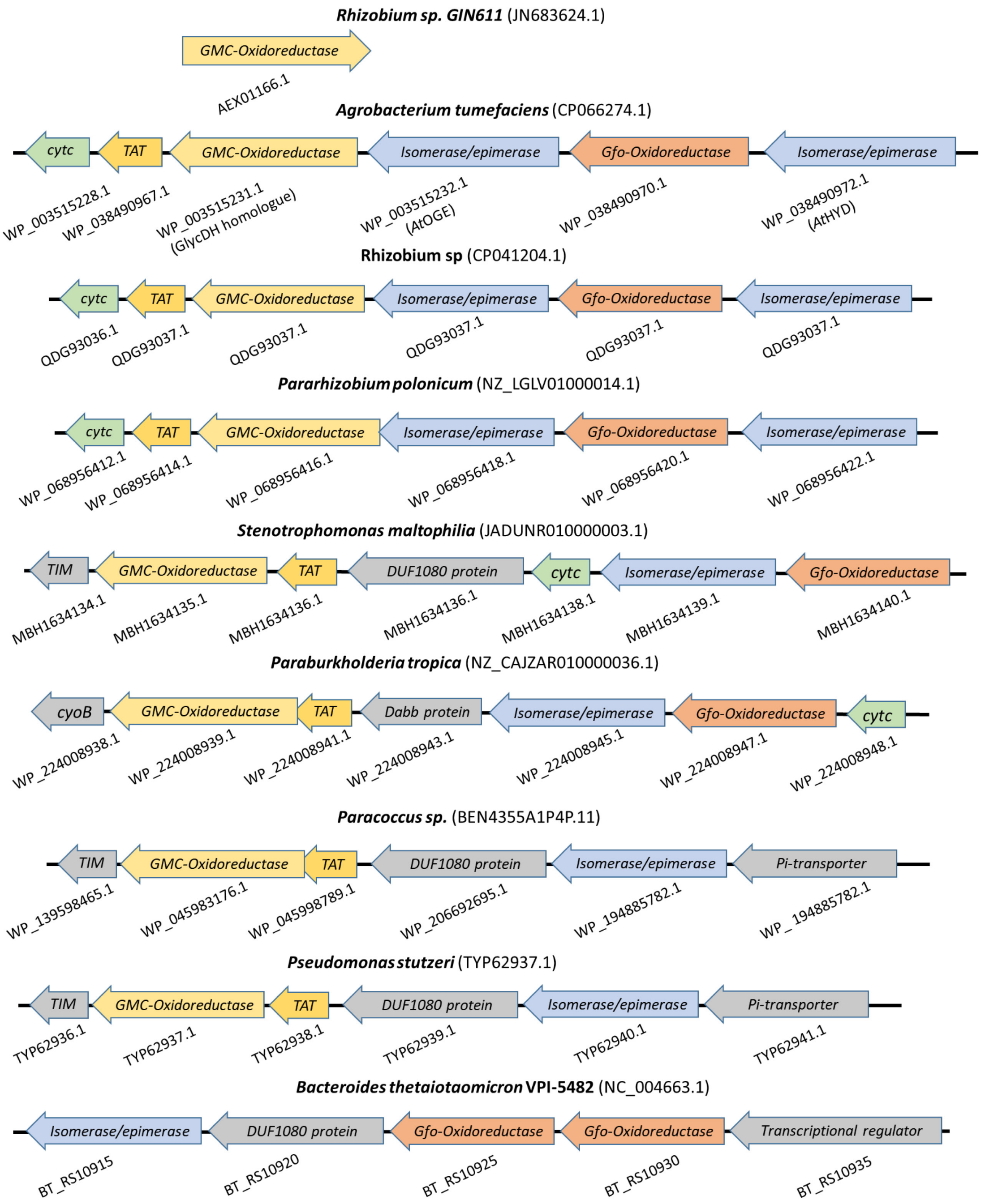

Supplementary Figure 18. Comparison of operons involving genes homologous to the GlycDH gene of *Rhizobium sp. GIN611.*[9] Microorganism names are shown in bold, NCBI reference sequence numbers in brackets, predicted protein families in arrows and respective NCBI reference sequences underneath. Enzyme families are depicted using identical colors, grey is used for various enzymes.

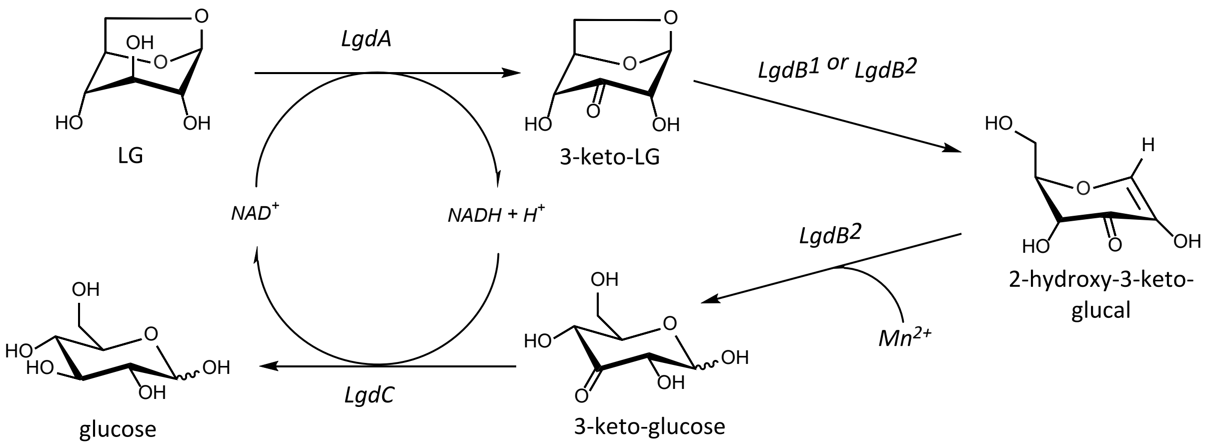

Supplementary Figure 19. Proposed levoglucosan (LG) degradation pathway from *Bacillus smithii* S-2701M by Yoshida *et al.*[10]

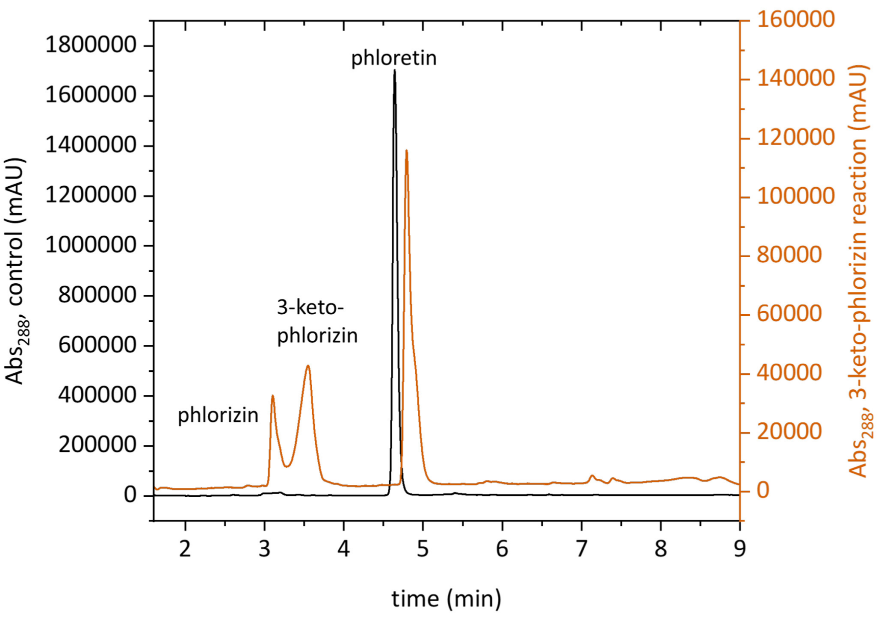

Supplementary Figure 20. HPLC chromatogram of an *At*OGE (putative sugar phosphate isomerase/epimerase; WP_003515232.1) catalyzed deglycosylation of 3-keto-phlorizin (n = 1 individual experiment). Isolated 3-keto-phlorizin was dissolved in 10 mM potassium phosphate buffer (pH 7.0), the reaction was started with *At*OGE (0.30 mg/mL) at 37 °C and agitation (650 rpm), quenched with MeCN and analyzed on HPLC. Besides the aglycone phloretin released from the substrate, unoxidized starting substrate phlorizin is visible. This is due to an incomplete reaction of GlycDH for 3-keto-phlorizin synthesis. Reaction products are shown as an orange trace, the phloretin standard is superimposed (black) and slightly shifted for clarity.

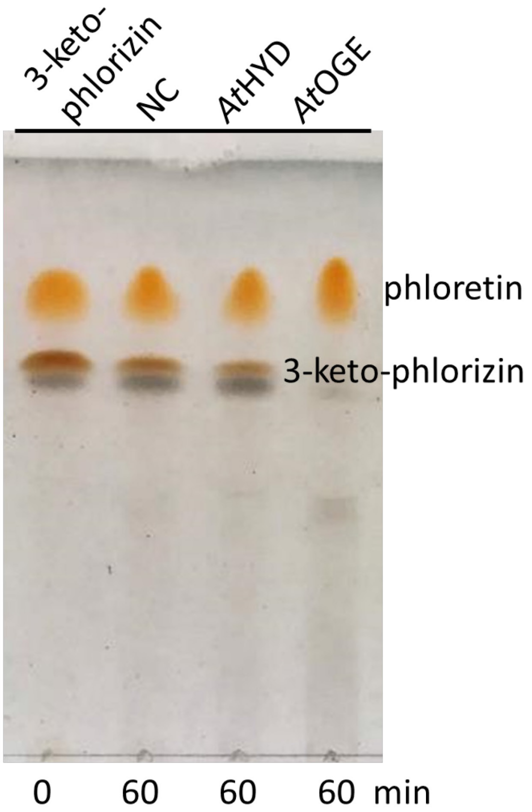

Supplementary Figure 21. TLC analysis of *At*OGE and *At*HYD (putative sugar phosphate isomerase/epimerase; WP_038490972.1) catalyzed elimination of 3-keto-phlorizin (n = 1 individual experiment). Negative control with no enzyme added (NC). Phloretin is present in each column due to spontaneous deglycosylation. As substrate, 10 mM of 3-keto-phlorizin were incubated with enzyme (0.30 mg/mL) in potassium phosphate buffer (10 mM; pH 7.0). Enzymes were pre-incubated with $MnCl_2$ (1.0 mM) for 120 min at 4.0 °C. The reaction was incubated at 37 °C and 650 rpm for 60 min. Compounds were separated with a 9:1 mobile phase of MeCN and H_2O. A *p*-anisaldehyde based stain was used. Source data are provided as a Source Data file.

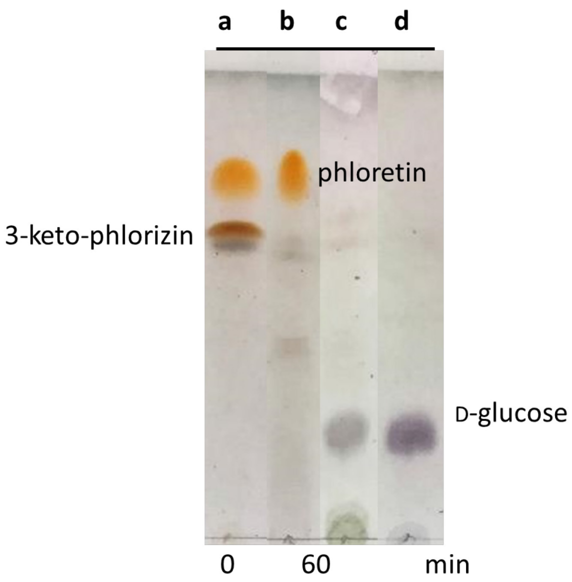

Supplementary Figure 22. TLC analysis of 3-keto-phlorizin conversion by enzymes of the glucoside degrading operon of *A. tumefaciens* (n = 1 individual experiment). Isolated 3-keto-phlorizin (2.0 mM) was used as starting substrate (**a**), incubation with *At*OGE yields phloretin (**b**). Incubation with *At*OGE, *At*HYD and Gfo oxidoreductase yields glucose (**c**). A glucose standard (10 mM) was used (**d**). All reactions were conducted in potassium phosphate buffer (10 mM; pH 7.0) at 37 °C under agitation (650 rpm) with 0.30 mg/mL of each enzyme. For reactions with Gfo oxidoreductase, 4.0 mM NADH were supplied. *At*OGE and *At*HYD were pre-incubated with $MnCl_2$ (1.0 mM; 120 min). Compounds were separated with a 9:1 mobile phase of MeCN with H_2O and TLC plates were stained with a *p*-anisaldehyde based stain. Source data are provided as a Source Data file.

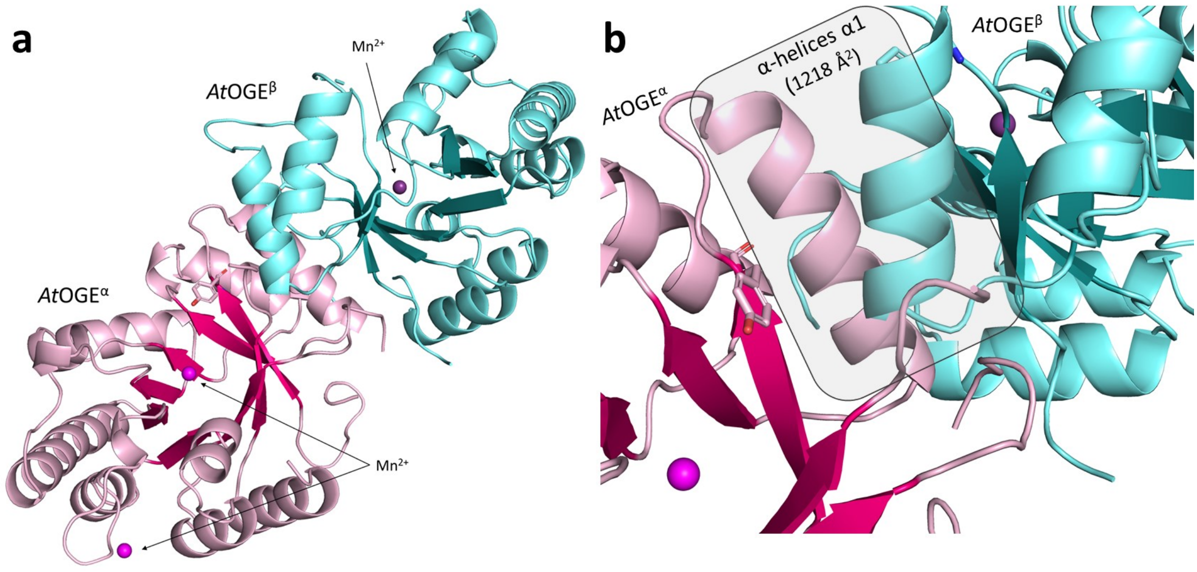

Supplementary Figure 23. *At*OGE 2.0 Å crystal structure in full view (**a**) and zoomed into dimer interface (**b**). Chain A (subunit α) of putative homodimer is colored in pink, chain B (subunit β) in cyan and metal ions (Mn^{2+}) present in the crystallization buffer in purple.

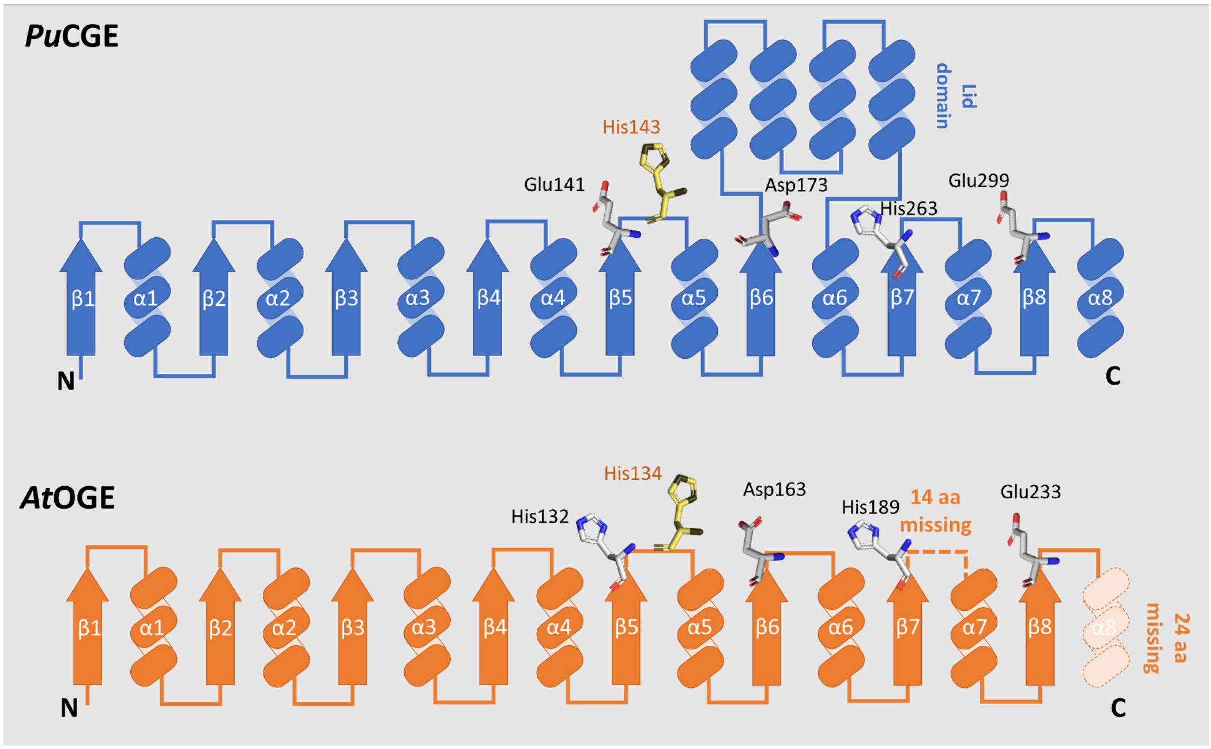

Supplementary Figure 24. Graphical representation of *Pu*CGE[8] subunit α and *At*OGE secondary structures. Key residues are shown schematically at their secondary structure position. Mn^{2+}-coordinating residues are shown in grey, catalytic residues in yellow. *At*OGE contains a short loop as similarly observed in the CGE from *Eubacterium cellulosolvens* (*Eu*CGE)[8]. The loop is located in a flexible region between β7 and α7 and unfortunately could not be modeled due to low electron density. Despite sharing a similar topology, the sequence similarity between *At*OGE and *Pu*CGEs is less than 30%.

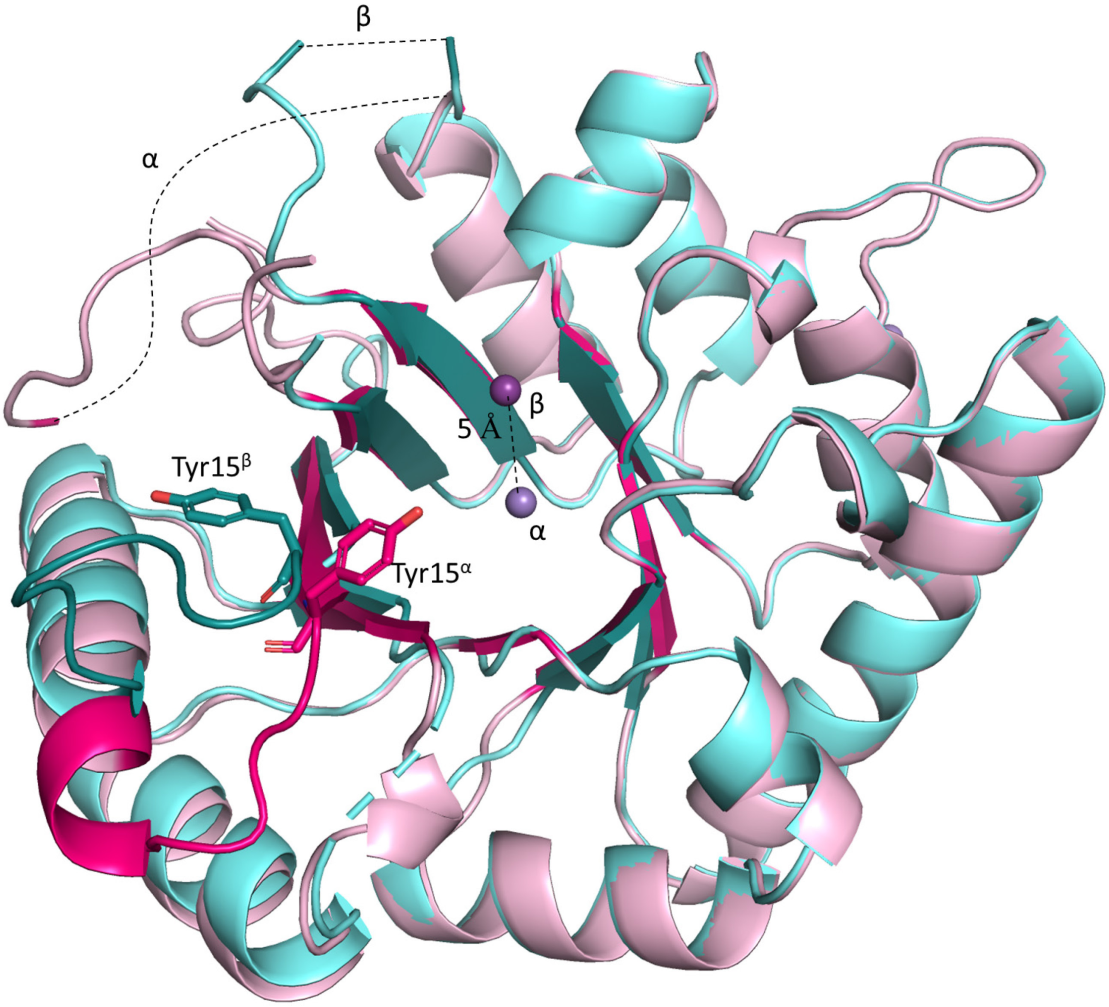

Supplementary Figure 25. Superposition of *At*OGE subunit α (magenta) and subunit β (cyan) with RMSD of 0.221 Å. Notable differences in the two subunits are: the position of the Mn^{2+} ion shown in in purple, the position of the loop (in saturated colors) adjacent to Tyr15, and the conformation of the missing loop between β7 and α7 (dashed line; Thr198 – Gly211).

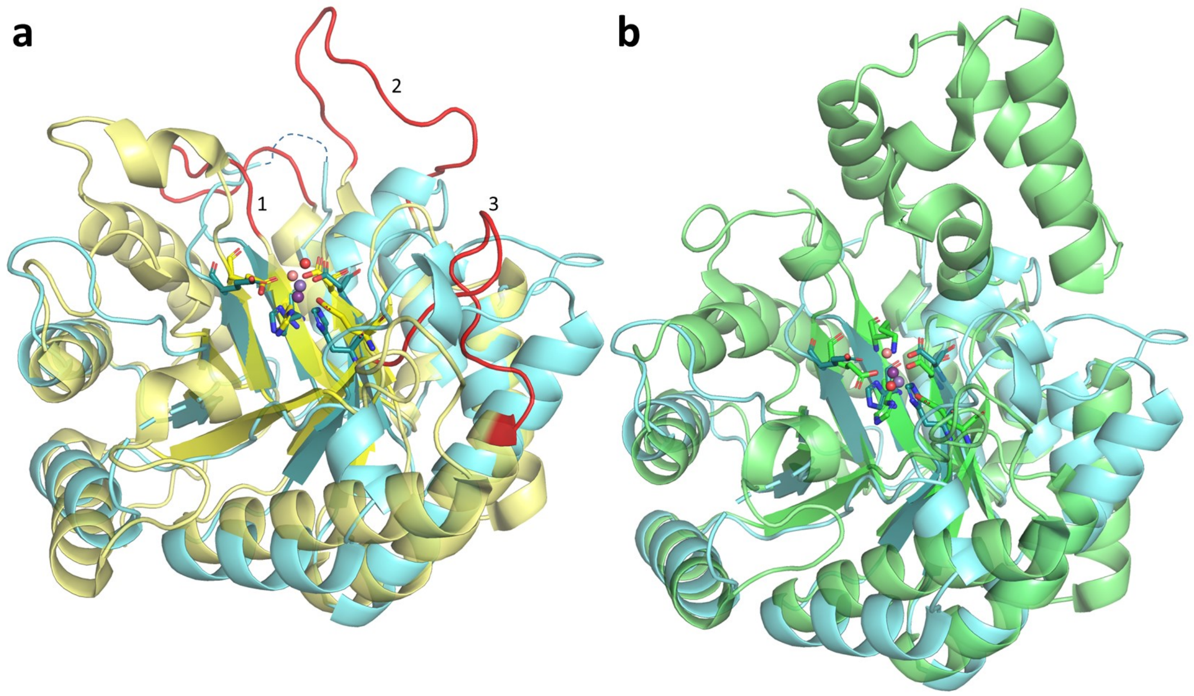

Supplementary Figure 26. Superposition of *At*OGE and CGEs. Subunit β of *At*OGE (cyan) is compared with subunit α of CGEs from *E. cellulosolvens*[8] (yellow) (**a**) and *A. globiformis*[8] (green) (**b**). Mn^{2+} ion (purple), Mn^{2+} coordinating residues and water (red) are shown. In **a**, three possible loop systems (red) for forming the active site cavity of *E. cellulosolvens* are proposed.

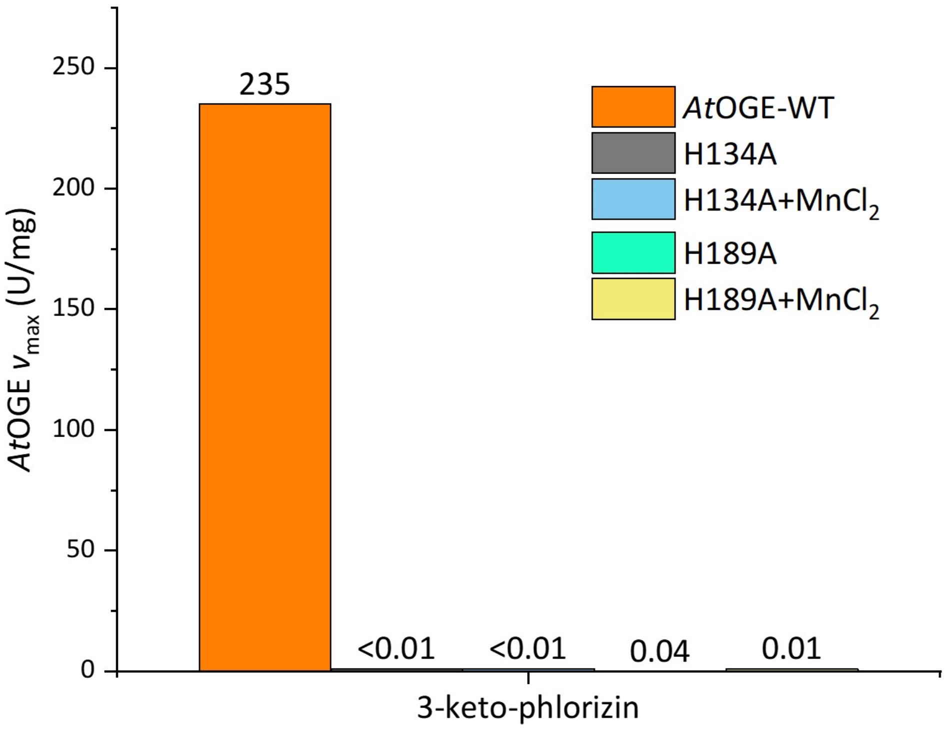

Supplementary Figure 27. *At*OGE variants catalyzing 3-keto-phlorizin elimination and their comparison to the wildtype enzyme. Initial rates were determined in potassium phosphate buffer (10 mM, pH 6.0) containing 5.0% (v/v) DMSO and 2.40 mg/mL enzyme (n = 1 individual experiment). Reactions were carried out at 37 °C and 650 rpm agitation for 60 min. In an attempt to saturate H189A and H134A mutant with metal cofactor, the enzyme was pre-incubated with $MnCl_2$ (5.0 mM) for 120 min at 4.0 °C prior to usage. Note that WT displays almost full activity (89%) without $MnCl_2$ preincubation (Supplementary Figure 40b). Samples were quenched with MeCN and analyzed on HPLC. WT rates are from initial rate measurements in Figure S39a. For further experimental details and for the analytical procedures used, see the Methods section of the main manuscript. Source data are provided as a Source Data file.

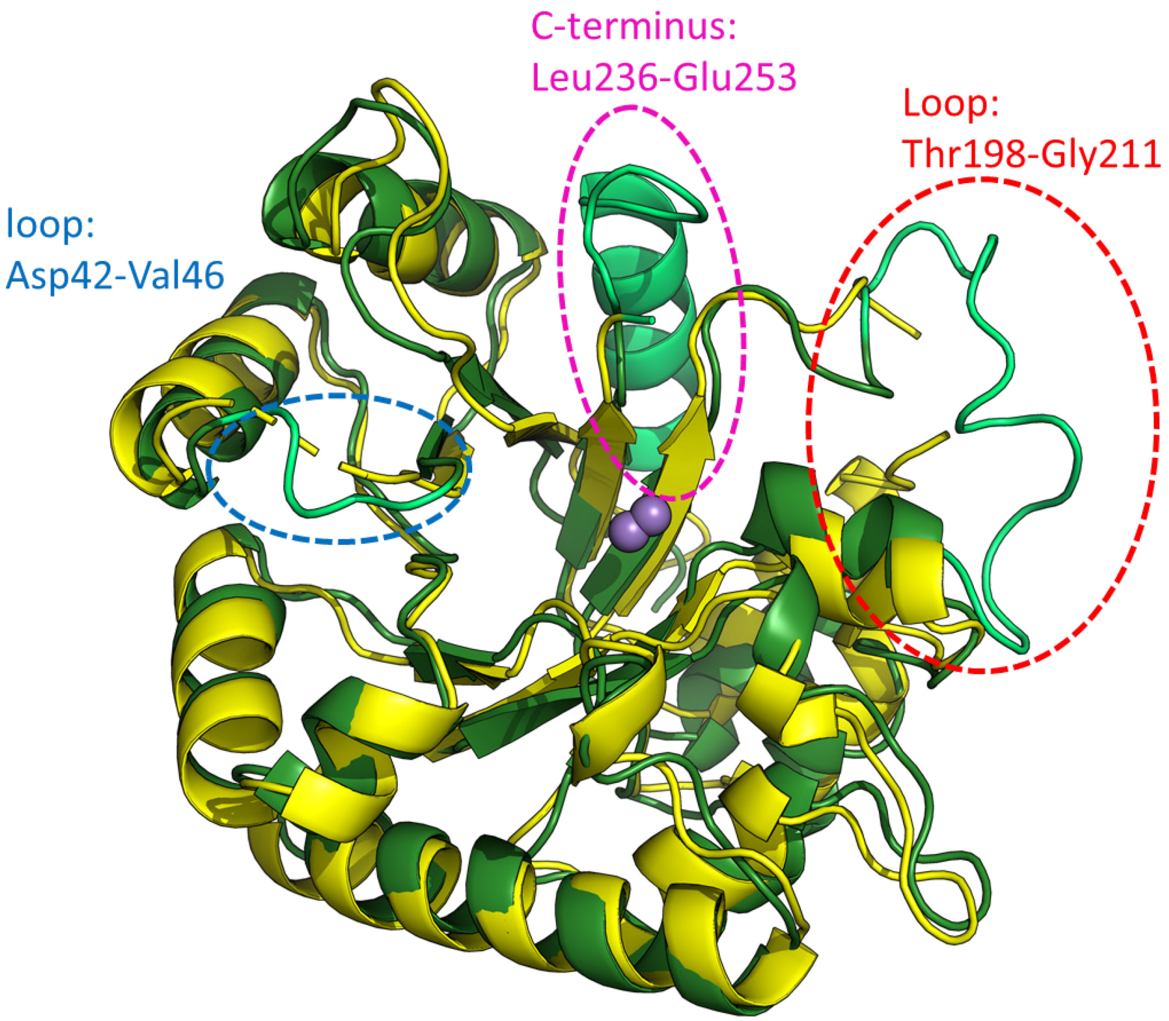

Supplementary Figure 28. Overlay of *At*OGE crystal structure (yellow) and structure with modelled loops (green). The missing loop between Asp42−Val46 is highlighted by a blue circle, the loop between Thr198−Gly211 is highlighted by a red circle and the modelled C-terminus is highlighted by a purple circle.

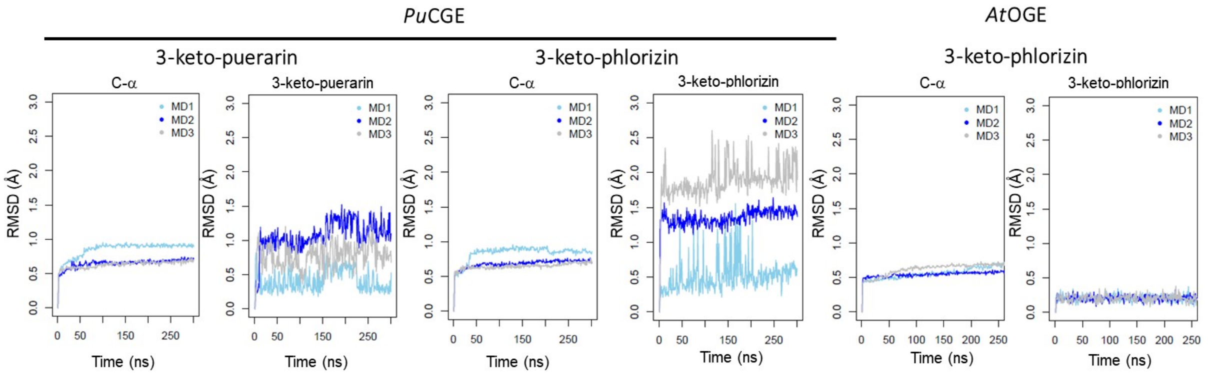

Supplementary Figure 29. RMSD values for enzyme and substrates during MD simulation. The main chain C-α atoms and substrate atoms were measured. The simulation was conducted three times (n = 3 individual simulations) and represented by grey, blue, and cyan lines. The C-α traces indicate that the protein remained stable throughout the simulation. The Substrates have higher fluctuations as compared to the C-α atoms, but remain in the active site throughout the simulation. Source data are provided as a Source Data file.

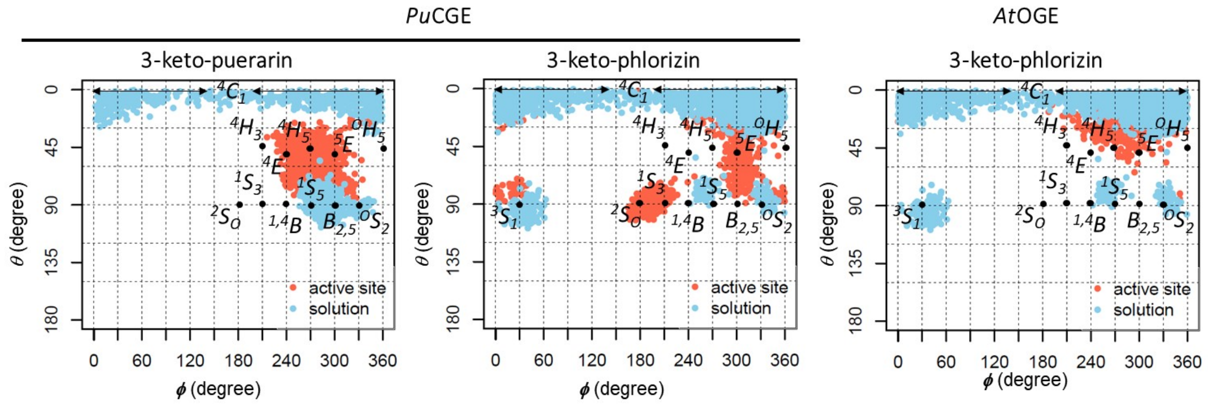

Supplementary Figure 30. Ring pucker analysis of the 3-keto-glycosyl moiety of 3-keto-puerarin and 3-keto-phlorizin in solution and in the active site of *Pu*CGE and *At*OGE. To evaluate the effect of the active site environment on the sugar ring puckering, two simulations of the ligand in water were performed and compared to those performed with the enzyme-ligand complexes. Ring pucker analyses were performed using the Cremer-Pople puckering descriptors Q, theta and phi. All simulations were performed on the force field level of theory. For further details, consult the Docking and molecular dynamics simulations sections in described in the methods. Source data are provided as a Source Data file.

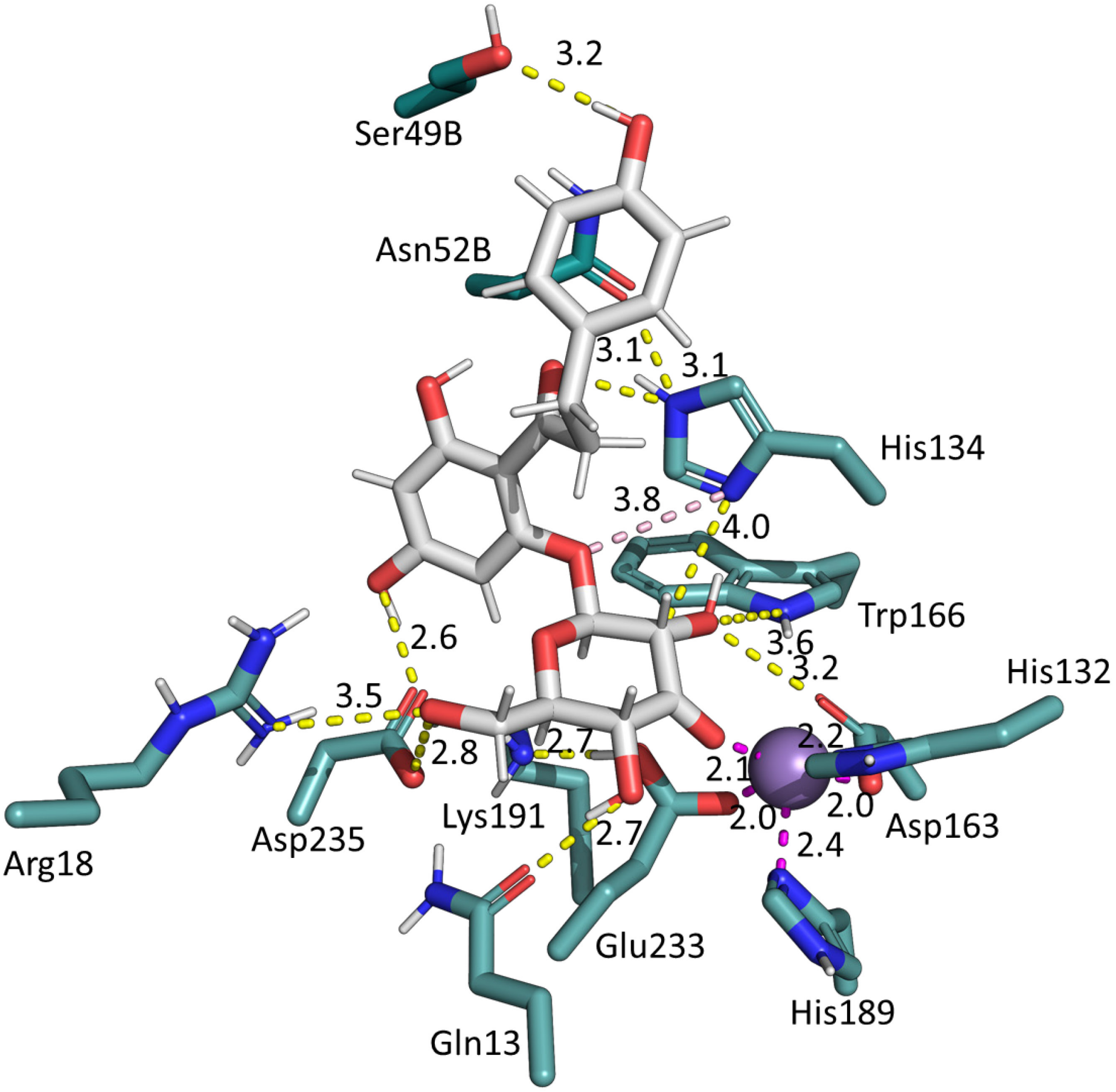

Supplementary Figure 31. MD-refined binding mode of 3-keto-phlorizin (C-atoms in white) to the active site of *At*OGE. A representative structure was selected from the most populated cluster of conformers out of three simulations (n = 3 individual experiments). Clustering was achieved using the hierarchical agglomerative (bottom-up) algorithm. All polar interactions between substrate and enzyme are shown. Dashed lines show interactions: hydrogen bonds in yellow; interactions with Mn^{2+} in magenta; and distance (in Å) between the catalytic base (*At*OGE: H134) and the glycosidic oxygen in light pink.

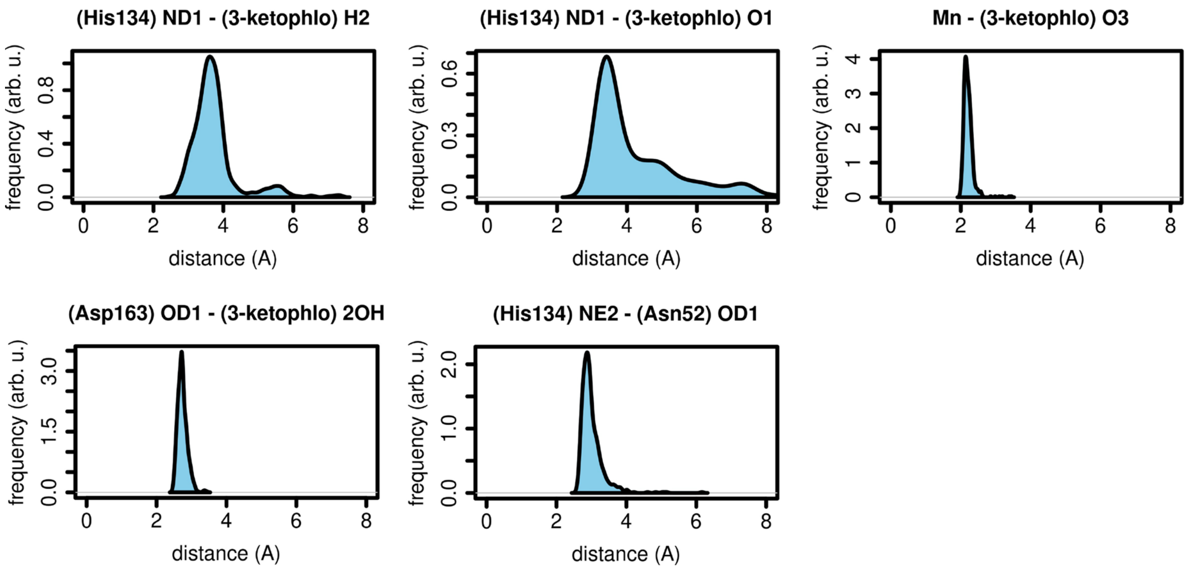

Supplementary Figure 32. Catalytically relevant interatomic distances in the active site of *At*OGE in complex with 3-keto-phlorizin. 3-ketophlo = 3-keto-phlorizin; Plots show the distance distribution observed during MD simulations (n = 3 individual simulations), the frequency is given as arbitrary units (arb. u.) and distances are given in Å. Source data are provided as a Source Data file.

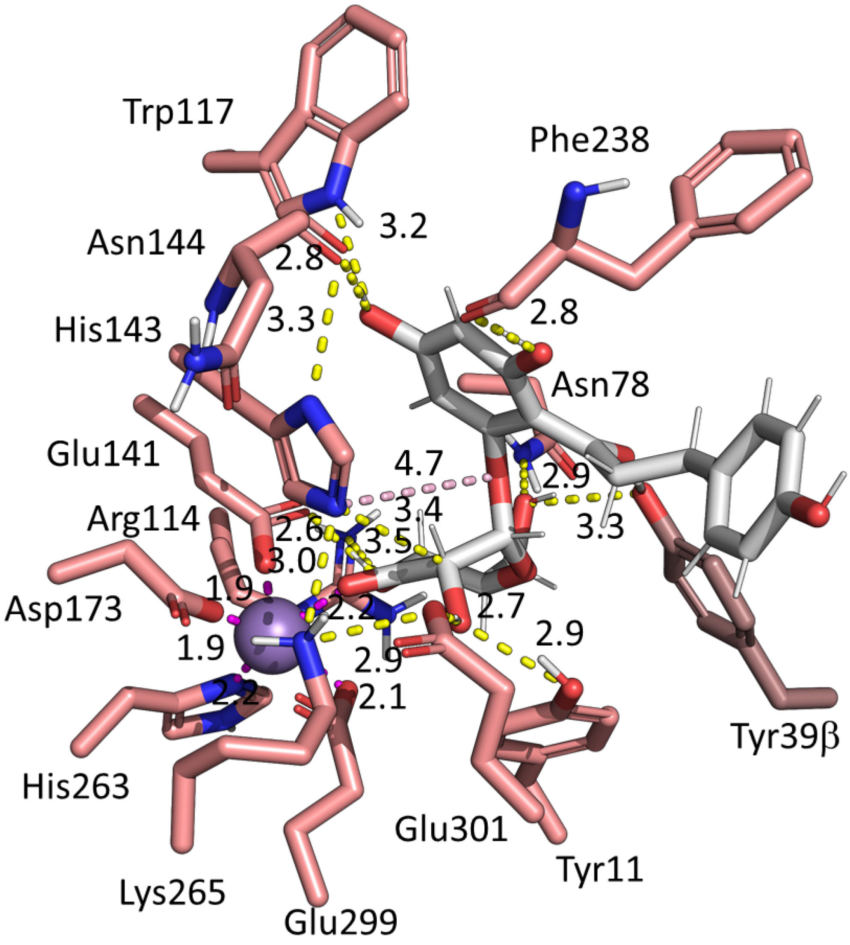

Supplementary Figure 33. MD-refined binding mode of 3-keto-phlorizin (C-atoms in white) to the active site of *Pu*CGE. A representative structure was selected from the most populated cluster of conformers out of three simulations (n = 3 individual experiments). Clustering was achieved using the hierarchical agglomerative (bottom-up) algorithm. All polar interactions between substrate and enzyme are shown. Residues from the β-subunit are shown in light brown. Dashed lines show interactions: hydrogen bonds in yellow; interactions with Mn^{2+} in magenta; and distance (in Å) between the catalytic base (*Pu*CGE: H143) and the glycosidic carbon in light pink.

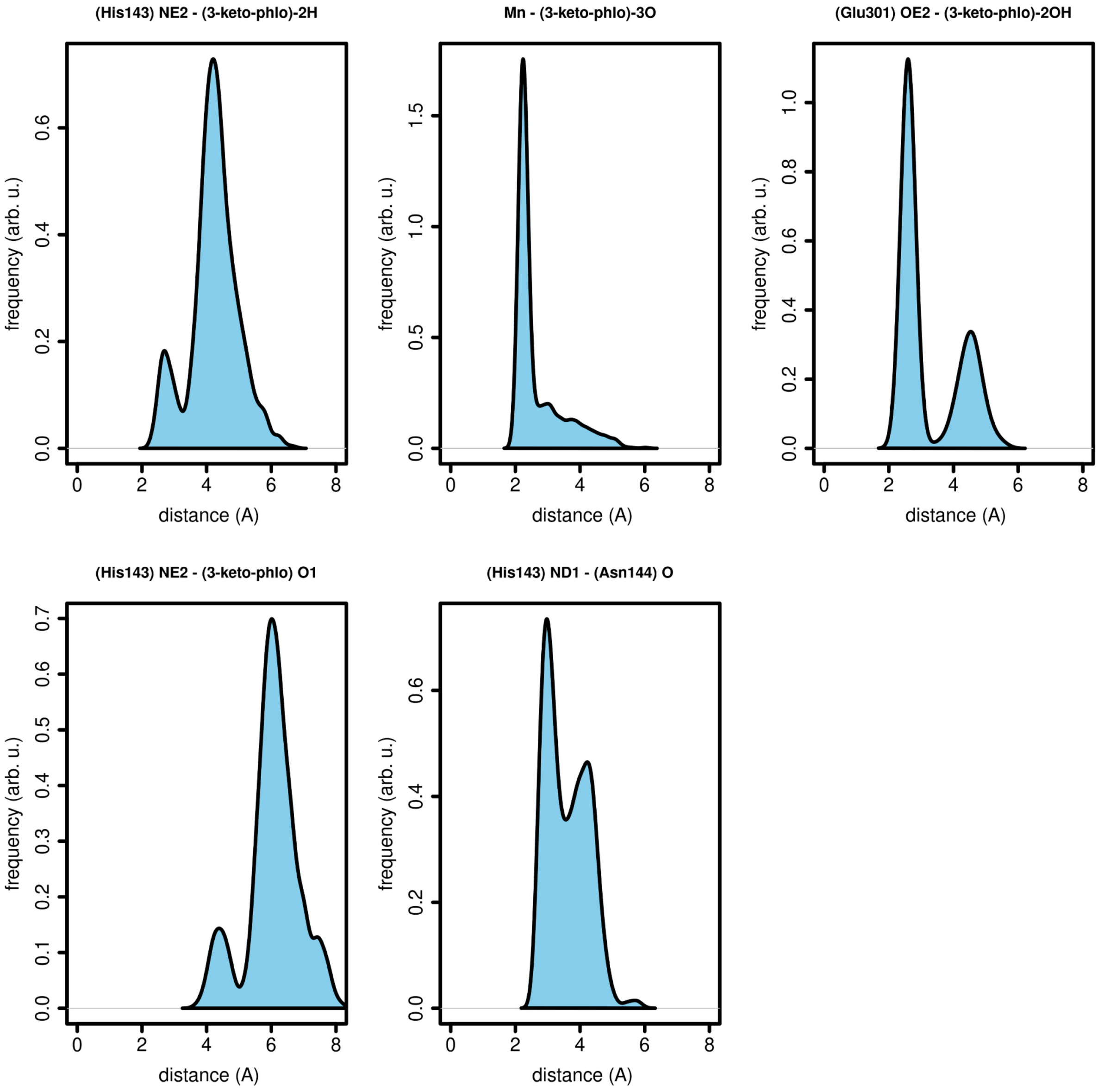

Supplementary Figure 34. Catalytically relevant interatomic distances in the active site of *Pu*CGE in complex with 3-keto-phlorizin. 3-keto-phlo = 3-keto-phlorizin; Plots show the distance distribution observed during MD simulations (n = 3 individual simulations), the frequency is given as arbitrary units (arb. u.) and distances are given in Å. Source data are provided as a Source Data file.

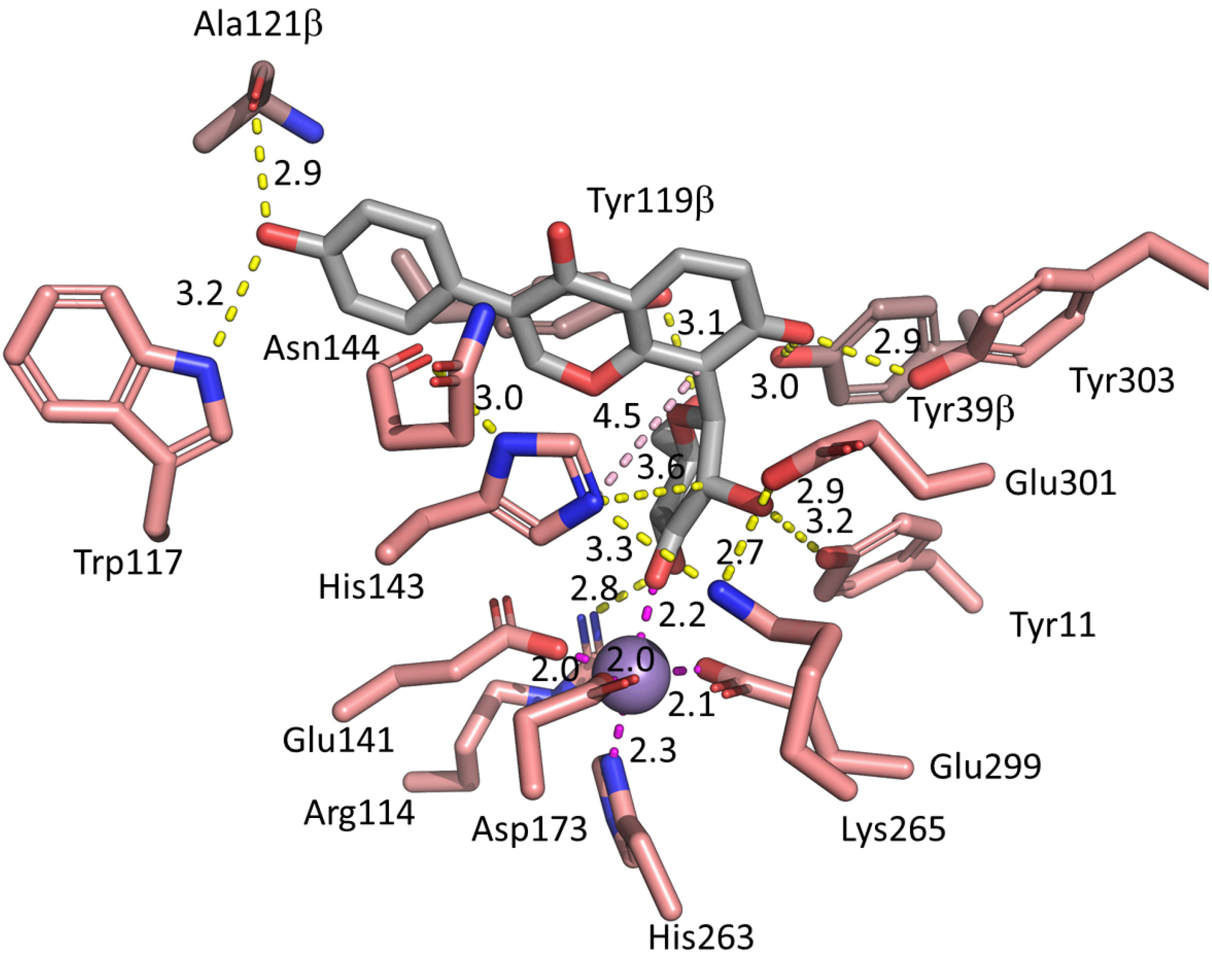

Supplementary Figure 35. MD-refined binding mode of 3-keto-puerarin (C-atoms in grey) to the active site of *Pu*CGE. A representative structure was selected from the most populated cluster of conformers out of three simulations (n = 3 individual experiments). Clustering was achieved using the hierarchical agglomerative (bottom-up) algorithm. All polar interactions between substrate and enzyme are shown. Residues from the β-subunit are shown in light brown. Dashed lines show interactions: hydrogen bonds in yellow; interactions with Mn^{2+} in magenta; and distance (in Å) between the catalytic base (*Pu*CGE: H143) and the glycosidic carbon in light pink.

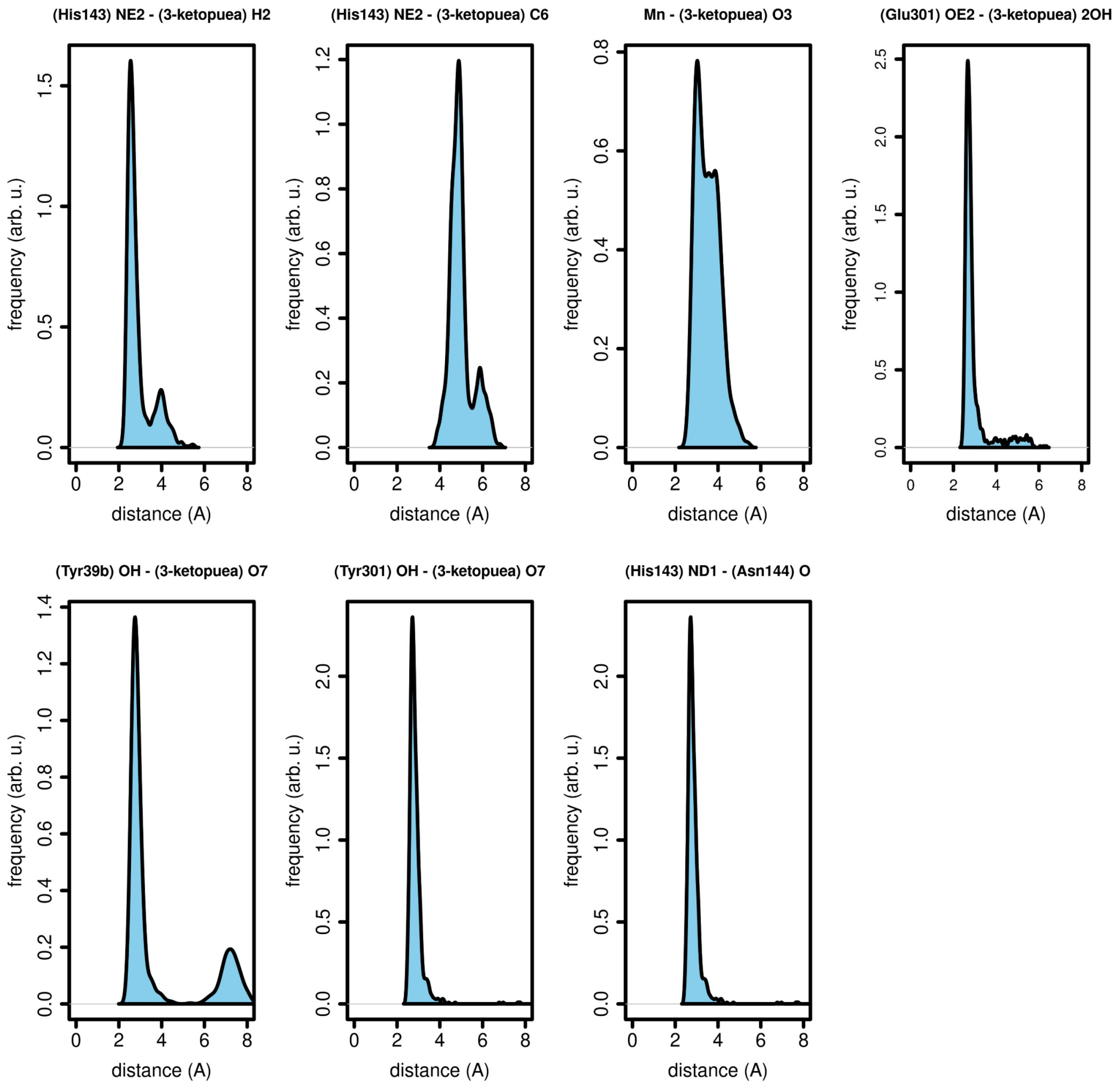

Supplementary Figure 36. Catalytically relevant interatomic distances in the active site of *Pu*CGE in complex with 3-keto-puerarin. 3-ketopuea = 3-keto-puerarin; Plots show the distance distribution observed during MD simulations (n = 3 individual simulations), the frequency is given as arbitrary units (arb. u.) and distances are given in Å. Source data are provided as a Source Data file.

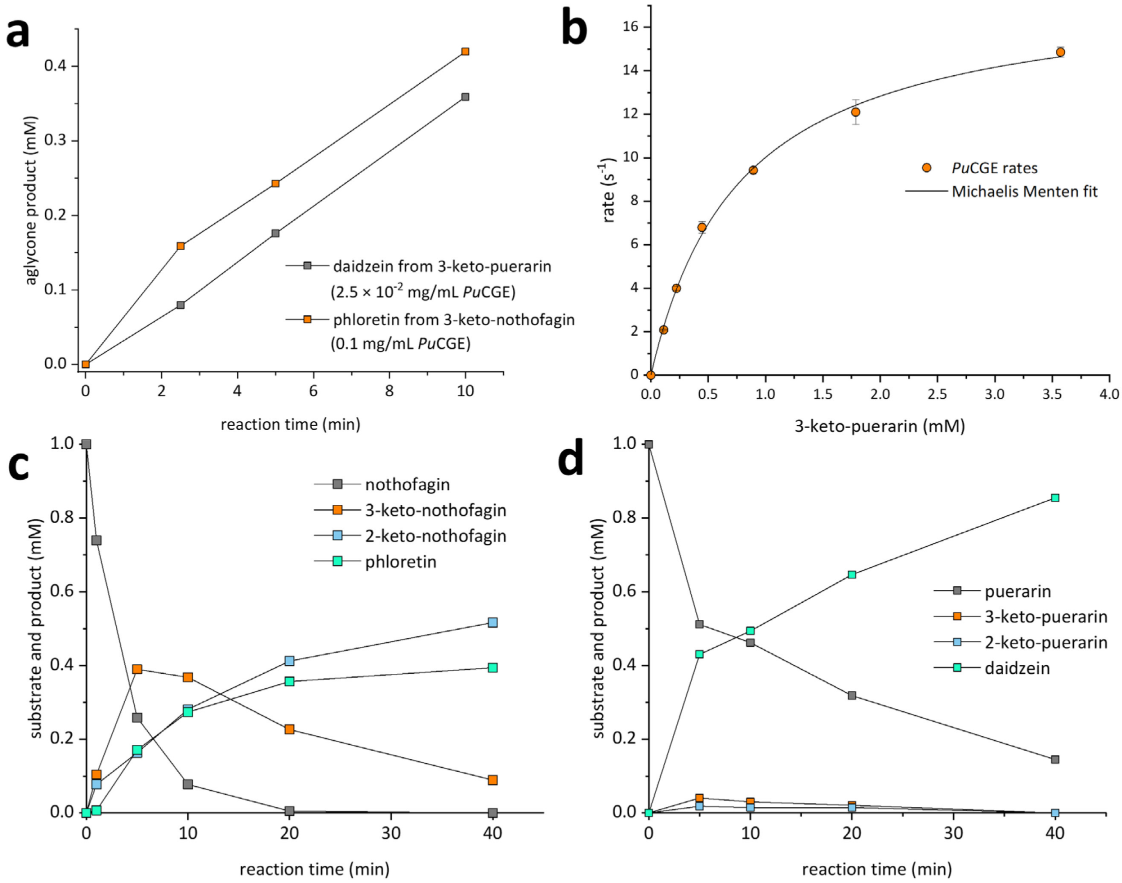

Supplementary Figure 37. *Pu*CGE-catalyzed elimination of keto-*C*-glycosides puerarin (**a**, **b**, **d**) and nothofagin (**a**, **c**). For single enzyme reactions **a** and **b**, C3 oxidized substrate was dissolved in potassium phosphate buffer (10 mM, pH 6.0, 5.0% DMSO) and incubated with *Pu*CGE, which was previously incubated with $MnCl_2$ (5.0 mM, 120 min). For one-pot reactions **c** and **d** substrate (1.0 mM) conditions were the same except $K_3[Fe(CN)_6]$ (8.0 mM), GlycDH (0.10 mg/mL) and preincubated *Pu*CGE (0.10 mg/mL) were added. All reactions were carried out at 37 °C and agitation (650 rpm). Samples were quenched with MeCN and analyzed by HPLC. Initial rates for the Michaelis Menten plot (**b**) were determined with 2.50×10^{-2} mg/mL of *Pu*CGE and 0.1–3.6 mM of 3-keto-puerarin and fitted to equation 2 using Origin software. The fit is shown as a solid line with S.D. indicated by error bars. (n = 1 individual experiment for **a**, **c** and **d**; n = 3 individual experiments for **b**). For further experimental details and for the analytical procedures used, see the Methods section of the main manuscript. Source data are provided as a Source Data file.

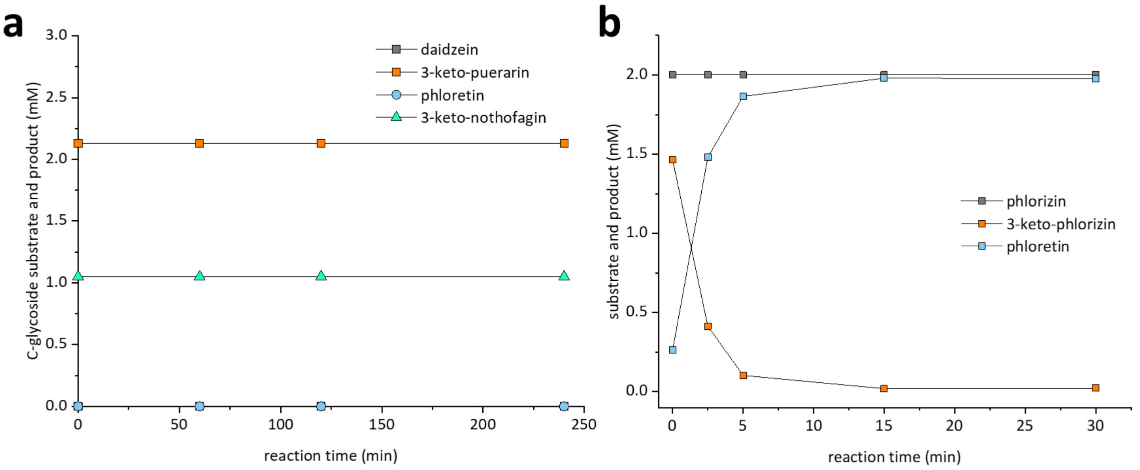

Supplementary Figure 38. Time courses of *At*OGE-catalyzed elimination of oxidized *C*-glycoside and oxidized/unoxidized *O*-glycoside substrates. **a.** 3-keto-puerarin and 3-keto-nothofagin. **b.** phlorizin and 3-keto-phlorizin. Initial rates were determined in potassium phosphate buffer (10 mM, pH 6.0, 5.0% (v/v) DMSO) with 0.50 mg/mL *At*OGE (preincubated with 5.0 mM $MnCl_2$ at 4.0 °C for 120 min) or 5.00 × 10^{-3} mg/mL for the 3-keto-phlorizin positive control (**b**). Reactions were carried out at 37 °C and agitation (650 rpm). Samples were quenched in MeCN and analyzed by HPLC (n = 1 individual experiment). For further experimental details and for the analytical procedures used, see the Methods section of the main manuscript. Source data are provided as a Source Data file.

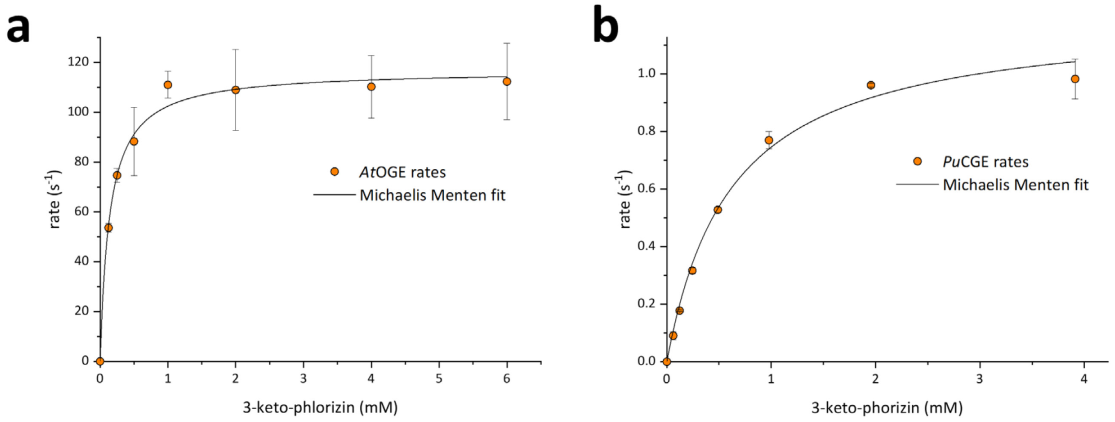

Supplementary Figure 39. Kinetic analysis of OGE- and CGE-catalyzed elimination of 3-keto-phlorizin. **a.** Kinetic analysis of *At*OGE (2.00 × 10^{-4} mg/mL) or **b.** *Pu*CGE (0.05 mg/mL) with 3-keto-phlorizin (0.0–6.0 and 0.0–4.0 mM, respectively). Potassium phosphate buffer (10 mM, pH 6.0) was used and reactions were incubated at 37 °C and agitation (650 rpm). Samples were quenched in MeCN and analyzed on HPLC (n = 3 individual experiments). Initial rates were fitted to the Michaelis Menten (equation 2) using Origin software. The fit is shown as a solid line with S.D. indicated by error bars. For further experimental details and for the analytical procedures used, see the Methods section of the main manuscript. Source data are provided as a Source Data file.

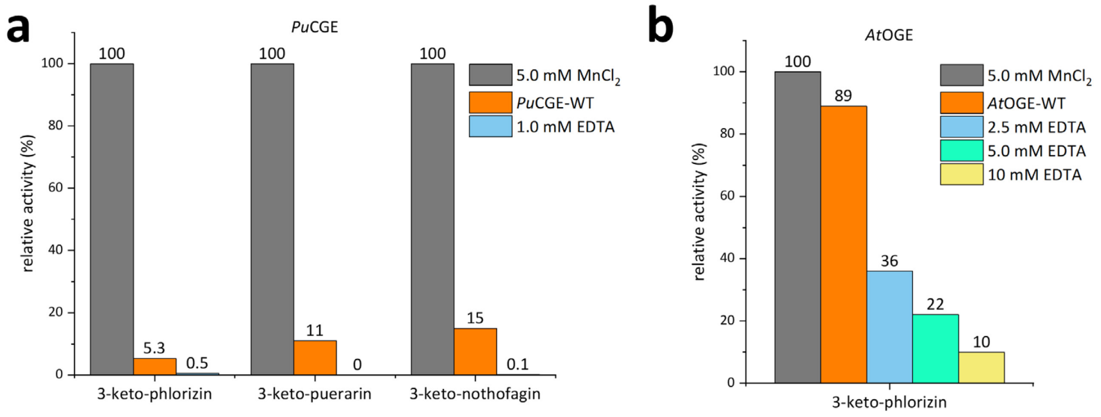

Supplementary Figure 40. Relative activity of **a.** *Pu*CGE- and **b.** *At*OGE in deglycosylation reactions of *O*- and *C*-glycosides in metal-chelating conditions. Relative activity was gained from dividing the specific activity of reactions by rates from reactions supplemented with $MnCl_2$. Enzymes were either used directly (WT), or preincubated with 5.0 mM $MnCl_2$ or in the persence of 1.0–10 mM EDTA, at 4.0 °C for 120 min. Reactions were conducted at 37 °C in potassium phosphate buffer (10 mM; pH 6.0; 5.0% DMSO) containing 2.0 mM substrate and 0.01 mg/mL of *At*OGE or 0.05 mg/mL of *Pu*CGE for 3-keto-puerarin and 0.10 mg/mL for both 3-keto-phlorizin and -nothofagin. Samples were quenched with MeCN and analyzed by HPLC (n = 1 individual experiment). For further experimental details and for the analytical procedures used, see the Methods section of the main manuscript. Source data are provided as a Source Data file.

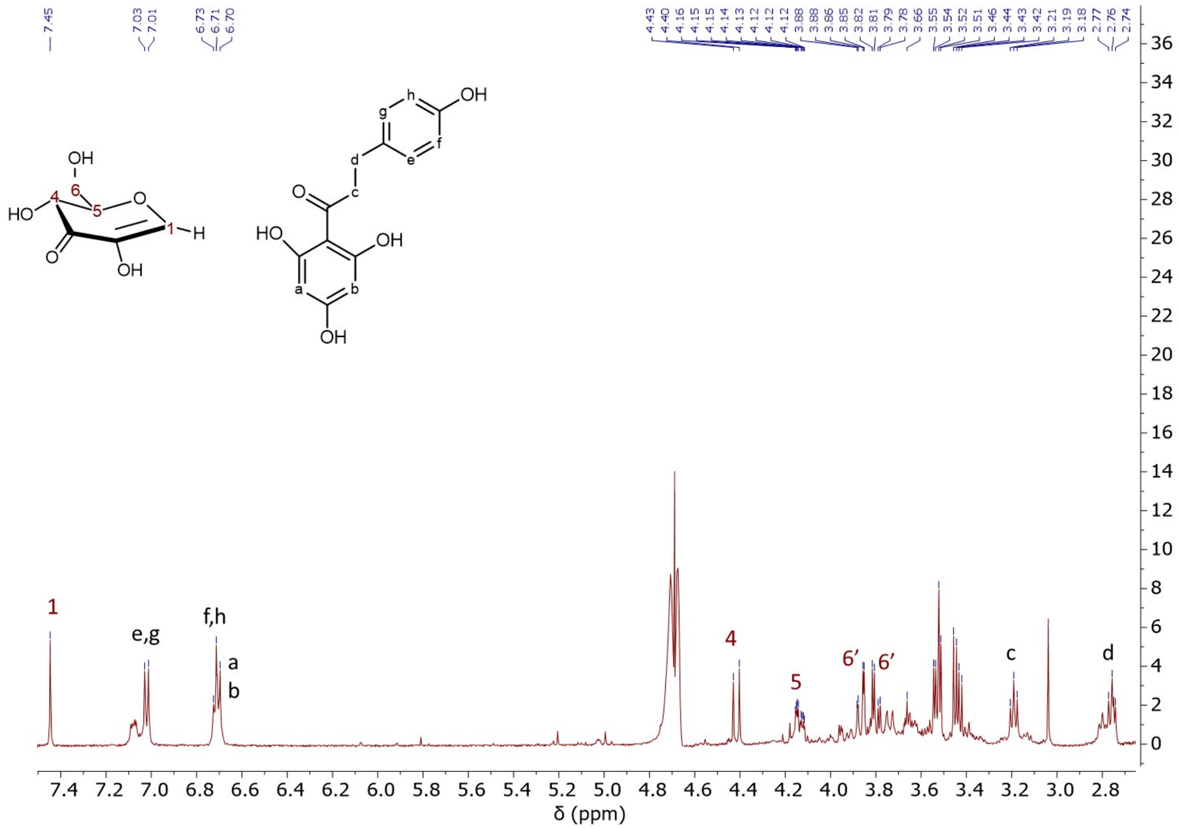

Supplementary Figure 41. ^{1}H NMR spectrum of products from *At*OGE reaction with 3-keto-phlorizin resulting in 3-keto-glucal and phloretin (500 MHz, 10% DMSO-d6, D_2O): δ 7.45 (s, 1H, H-1), 7.02 (d, J = 8.5 Hz, 2H, H-e and H-g), 6.72 (d, J = 6.0 Hz, 2H, H-f and H-h), 6.70 (s, 2H, H-a and H-b), 4.42 (d, J = 13.3 Hz, 1H, H-4), 4.14 (m, J = 13.2, 4.4, 2.2 Hz, 1H, H-5), 3.87 (dd, J = 13.0, 2.4 Hz, 1H, H-6), 3.80 (dd, J = 12.9, 4.5 Hz, 1H, H-6), 3.65–3.42 (m, Glycerol) 3.19 (t, J = 7.6 Hz, 2H, H-c), 2.76 (t, J = 7.5 Hz, 2H, H-d). Minor impurities of glycerol are visible (3.67–3.42 ppm).

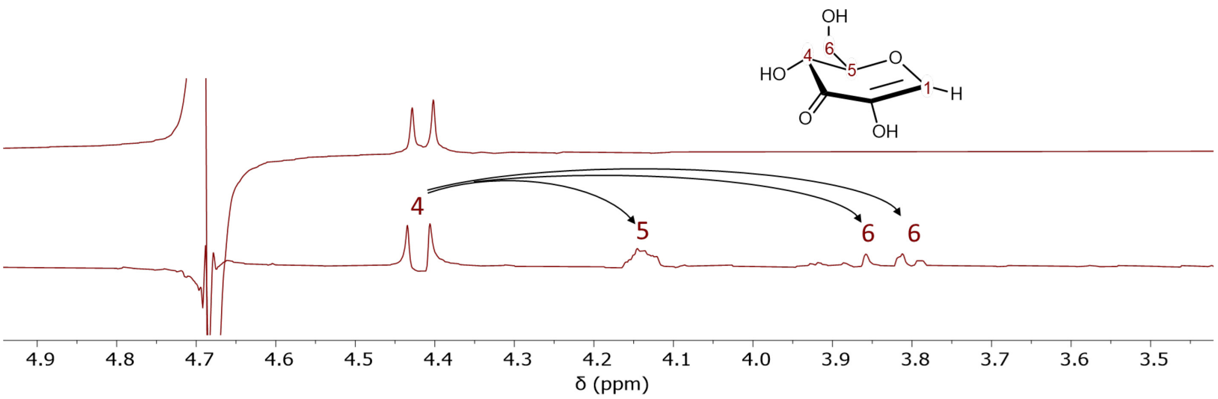

Supplementary Figure 42. TOCSY analysis from 2-hydroxy-3-keto-glucal product of *At*OGE-catalyzed 3-keto-phlorizin deglycosylation (500 MHz, 10% DMSO-d6, D_2O). Selective band center: 4.41 (ppm); width: 34.5 (Hz). Correlation between H-4, H-5, H6 and H6 as one spin system are displayed.

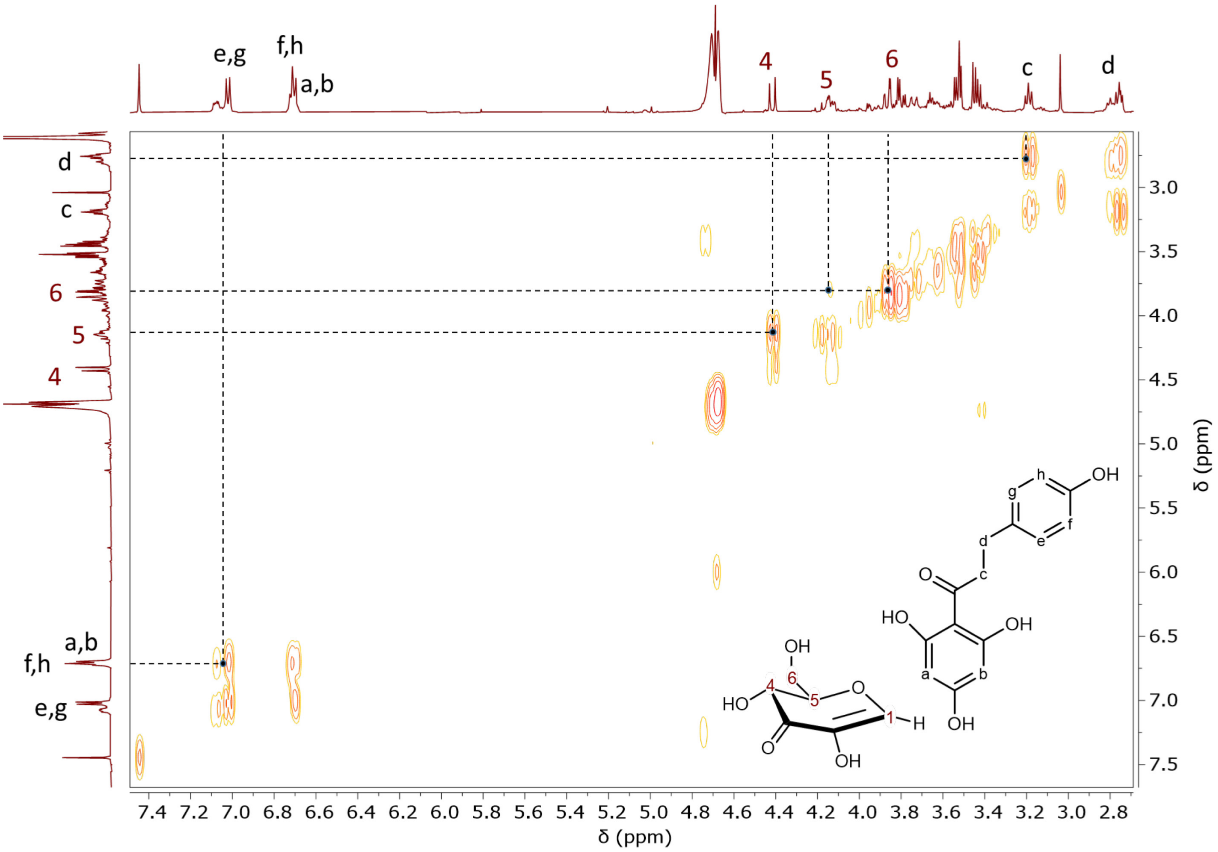

Supplementary Figure 43. COSY analysis of products from *At*OGE reaction with 3-keto-phlorizin resulting in 2-hydroxy-3-keto-glucal and phloretin (500 MHz, 10% DMSO-d6, D_2O). 2D-NMR shows proton coupling of H-e and H-g with H-f and H-h, H-4 with H-5, H-5 with H-6, H-6 with H-6, H-c with H-d.

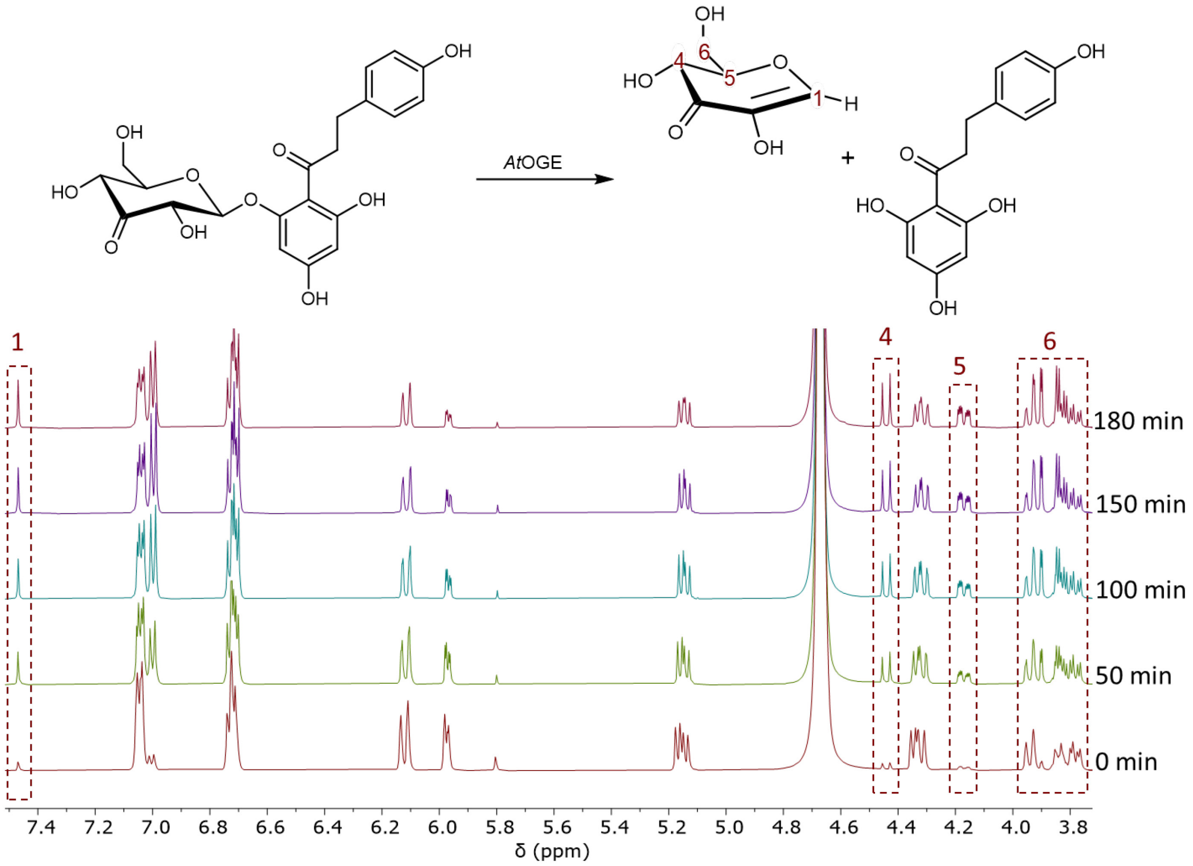

Supplementary Figure 44. In situ ^{1}H NMR analysis of *At*OGE reaction with 3-keto-phlorizin. The reaction results in 2-hydroxy-3-keto-glucal and phloretin (500 MHz, 10% DMSO-d6, 50 mM potassium phosphate buffer in D_2O (pD 7.4)). The proton signals from the sugar product 2-hydroxy-3-keto-glucal are framed and labelled in red. The reaction was measured over a time course of 180 min. For further experimental details and for the analytical procedures used, see the Methods section of the main manuscript.

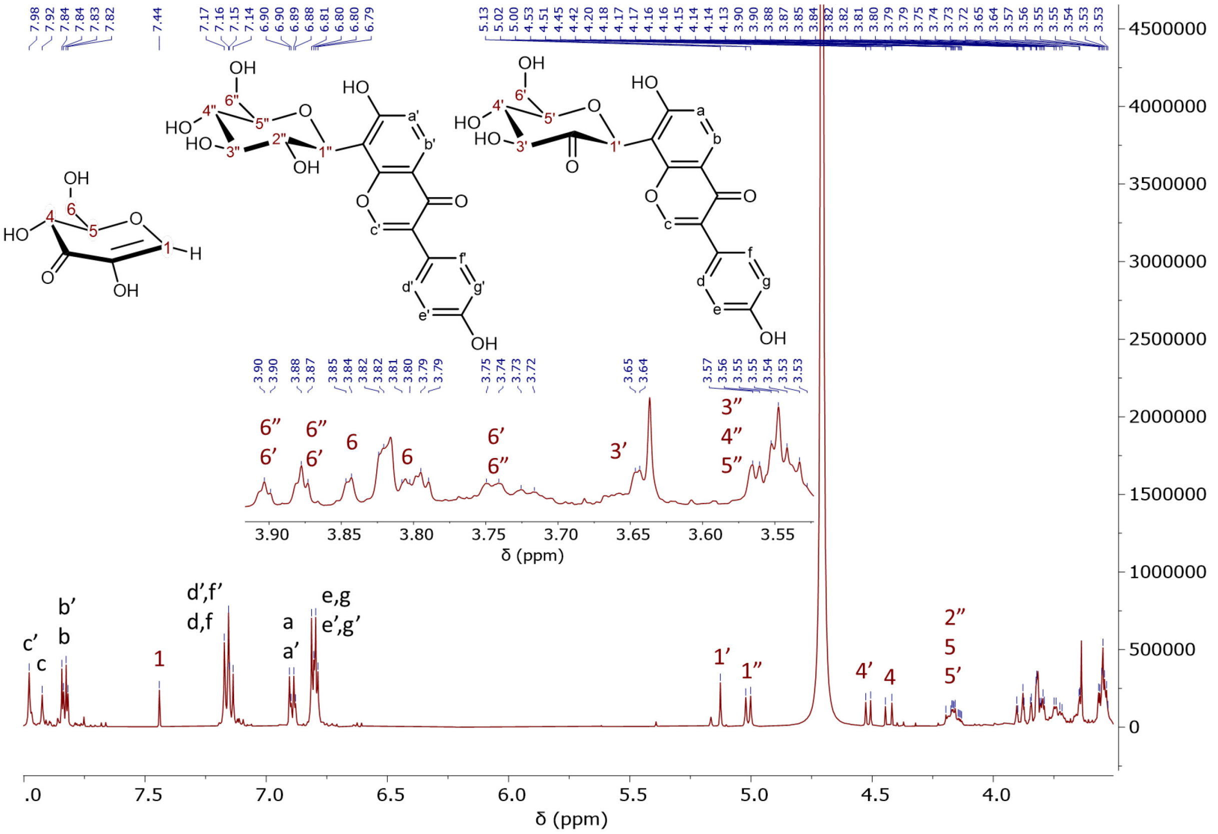

Supplementary Figure 45. ^{1}H NMR spectrum of product from *Pu*CGE reaction with 2-/3-keto-puerarin resulting in 2-hydroxy-3-keto-glucal and daidzein (not dissolved in the sample) with residuals of 2-keto-puerarin and puerarin due to incomplete conversion (500 MHz, 10% DMSO-d6, D_2O): δ 7.98 (s, 1H, H-c’), 7.92 (s, 1H, H-c), 7.83 (d, J = 8.9 Hz, 1H, H-b’), 7.83 (d, J = 8.9 Hz, 1H, H-b), 7.44 (s, 1H, H-1), 7.16 (d, J = 8.8 Hz, 2H, H-d’ and H-f’), 7.14 (d, J = 7.6 Hz, 2H, H-d and H-f), 6.90 (d, J = 9.0 Hz, 1H, H-a’), 6.89 (d, J = 9.0 Hz, 1H, H-a), 6.80 (d, J = 8.6 Hz, 2H, H-e’ and H-g’), 6.80 (d, J = 8.6 Hz, 2H, H-e and H-g), 5.13 (s, 1H, H-1’), 5.01 (d, J = 10.0 Hz, 1H, H-1”), 4.52 (d, J = 10.1 Hz, 1H, H-4’), 4.43 (d, J = 13.4 Hz, 1H, H-4), 4.19 (d, J = 10.2 Hz, 1H, H-2”), 4.15 (m, J = 13.3, 4.5, 2.3 Hz, 2H, H-5 and H-5’), 3.89 (dd, J = 12.9, 2.2 Hz, 2H, H-6” and H-6’), 3.83 (dd, J = 11.2, 1.9 Hz, 1H, H-6), 3.83–3.77 (m, 1H, H-6), 3.73 (dd, J = 12.1, 4.5 Hz, 2H, H-6” and H-6’), 3.64 (d, J = 10.0 Hz, 1H, H-3’), 3.59–3.51 (m, 3H, H-3”, H-4” and H-5”)

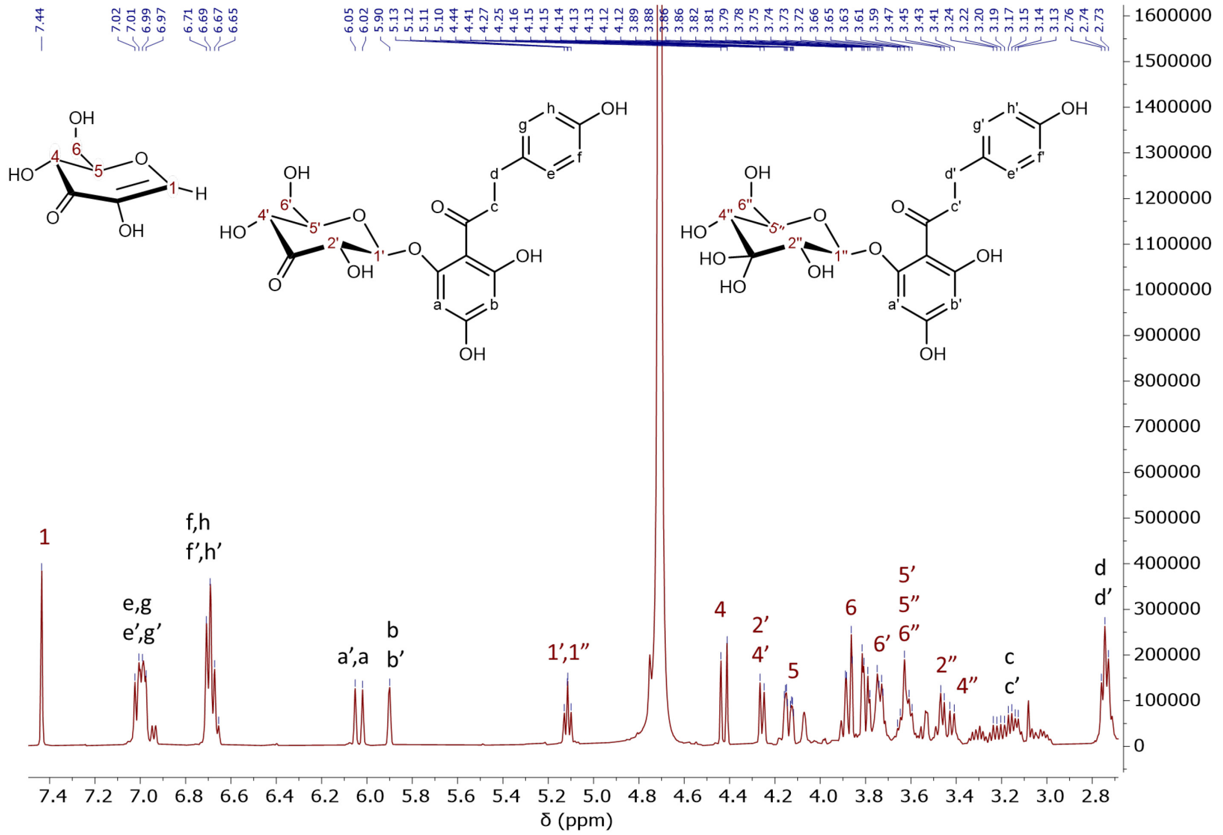

Supplementary Figure 46. ^{1}H NMR spectrum of products from *Pu*CGE reaction with 3-keto-phlorizin resulting in 2-hydroxy-3-keto-glucal and phloretin (not dissolved in the sample) as well as 3-keto-phlorizin and 3-diol-phlorizin due to incomplete conversion (500 MHz, 10% DMSO-d6, D_2O): δ 7.44 (s, 1H, H-1), 7.02 (d, J = 8.9 Hz, 2H, H-e and H-g), 6.98 (d, J = 8.1 Hz, 2H, H-e' and H-g'), 6.70 (d, J = 8.5 Hz, 2H, H-f' and H-h'), 6.66 (d, J = 9.0 Hz, 2H, H-f and H-h), 6.05 (s, 1H, H-a'), 6.02 (s, 1H, H-a), 5.90 (s, 2H, H-b and H-b'), 5.12 (d, J = 6.9 Hz, 1H, H-1'), 5.11 (d, J = 7.4 Hz, 1H, H-1"), 4.42 (d, J = 13.8 Hz, 1H, H-4), 4.26 (d, J = 9.0 Hz, 2H, H-2' and H-4'), 4.14 (m, J = 13.4, 4.5, 2.3 Hz, 1H, H-5), 3.87 (dd, J = 12.7, 2.3 Hz, 1H, H-6), 3.80 (dd, J = 12.8, 4.3 Hz, 1H, H-6), 3.74 (dd, J = 9.1, 3.4 Hz, 1H, H-6'), 3.68–3.58 (m, 3H, H-5', H-5" and H-6"), 3.46 (d, J = 7.6 Hz, 1H, H-2"), 3.42 (d, J = 9.6, 1H, H-4"), 3.21–3.10 (m, 4H, H-c and H-c'), 2.74 (t, 4H, H-d and H-d')

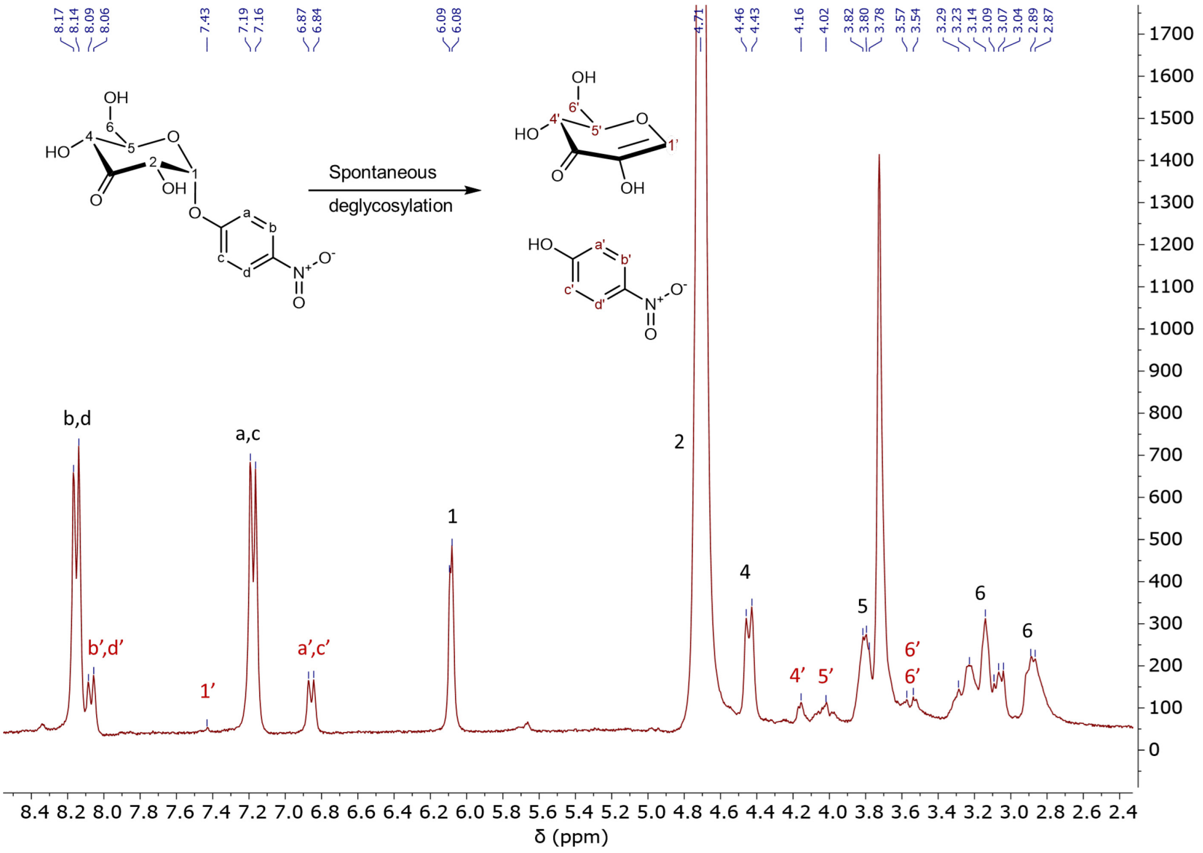

Supplementary Figure 47. ^{1}H NMR spectrum of 4-nitrophenyl α-D-*arabino*-hexopyranosid-3-ulose (4NPα3ketoG) and spontaneous deglycosylation products 4-nitrophenol and 1,5-anhydro-D-*erythro*-hex-1-en-3-ulose (2-hydroxy-3-keto-glucal) (300 MHz, D_2O): δ 8.15 (d, J = 8.7 Hz, 2H, H-b and H-d), 8.07 (d, J = 8.8 Hz, 2H, H-b' and H-d'), 7.43 (s, 1H, H-1'), 7.18 (d, J = 8.6 Hz, 2H, H-a and H-c), 6.86 (d, J = 8.6 Hz, 2H, H-a' and H-c'), 6.09 (d, J = 4.3 Hz, 1H, H-1), 4.71 (s, 1H, H-2), 4.44 (d, J = 9.4 Hz, 1H, H-4), 4.16 (s, 1H, H-4'), 4.02 (s, 1H, H-5'), 3.77 (m, 1H, H-5), 3.57 (s, 1H, H-6'), 3.54 (s, 1H, H-6'), 3.33–2.78 (m, 2H, H-6 and H-6).

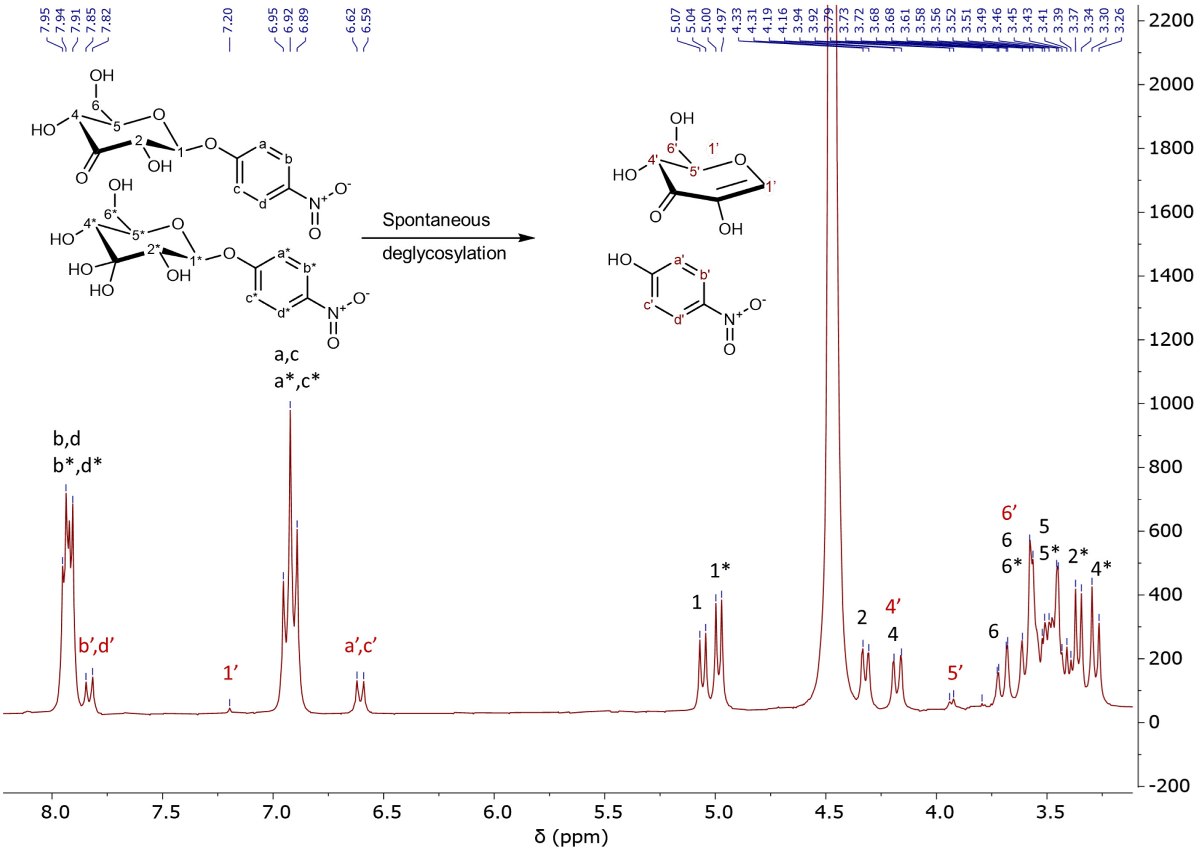

Supplementary Figure 48. ^{1}H NMR spectrum of 4-nitrophenyl β-D-*arabino*-hexopyranosid-3-ulose (4NPβ3ketoG), present partly in hydrated (gem-diol) form (60%); Spontaneous deglycosylation to products 4-nitrophenol and 2-hydroxy-3-keto-glucal (300 MHz, D_2O): δ 7.99-7.87 (m, 4H, H-b, H-d, H-b* and H-d*), 7.83 (d, J = 9.1 Hz, 2H, H-b' and H-d'), 7.20 (s, 1H, H-1'), 6.92 (m, 4H, H-a, H-c, H-a* and H-c*), 6.61 (d, J = 9.0 Hz, 2H, H-a' and H-c'), 5.06 (d, J = 7.9 Hz, 1H, H-1), 4.98 (d, J = 7.9 Hz, 1H, H-1*), 4.32 (d, J = 7.8 Hz, 1H, H-2), 4.17 (d, J = 10.1 Hz, 2H, H-4, H-4'), 3.93 (d, J = 5.3 Hz, 1H, H-5'), 3,70 (dd, J = 12.2, 2.0 Hz, 1H, H-6), 3.59 (m, 5H, H-6, H-6*, H-6*, H-6' and H-6'), 3.46 (m, 2H, H-5 and H-5*), 3.36 (d, J = 7.9 Hz, 1H, H-2*), 3.28 (d, J = 9.6 Hz, 1H, H-4*)

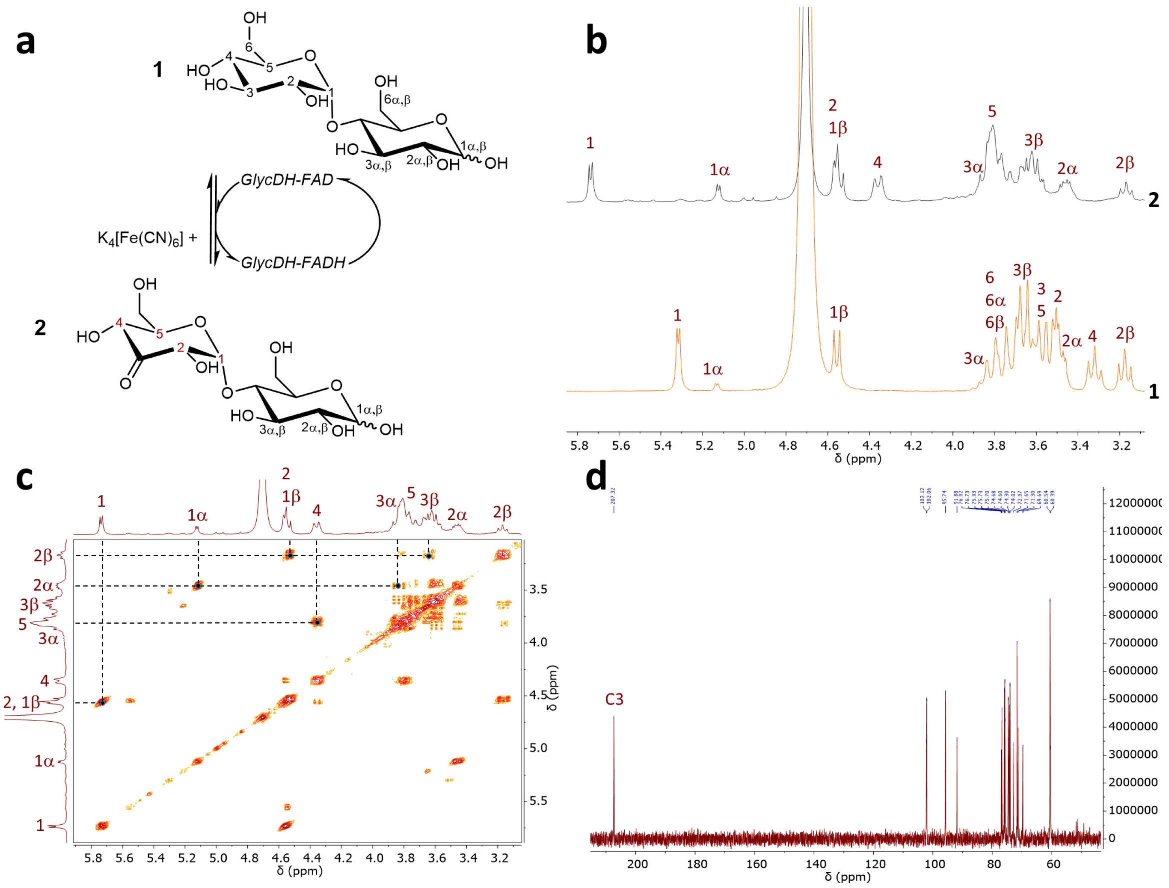

Supplementary Figure 49. Product identification from the GlycDH-catalyzed reaction with maltose. **a.** Reaction scheme for the conversion of maltose (**1**) into 3'-keto-maltose (**2**). **b.** Stacked substrate (**1**) and product (**2**) ^{1}H NMR spectra showing the missing H-3 signal in **2**, indicating full conversion of maltose into 3'-keto-maltose. The signals from H-1, H-2 and H-4 are shifted downfield due to the deshielding effect from the C3 keto-moiety. **c.** COSY analysis of 3'-keto-maltose. The relevant coupling interactions are indicated with dashed lines. No cross peak is observed for H-2, proving the oxidation at C3. **d.** ^{13}C NMR spectrum showing the expected chemical shift of a carbonyl carbon (~209 ppm) due to oxidation at C3. All the NMR experiments were performed in D_2O (^{1}H NMR, 300 MHz; ^{13}C NMR, 126 MHz).

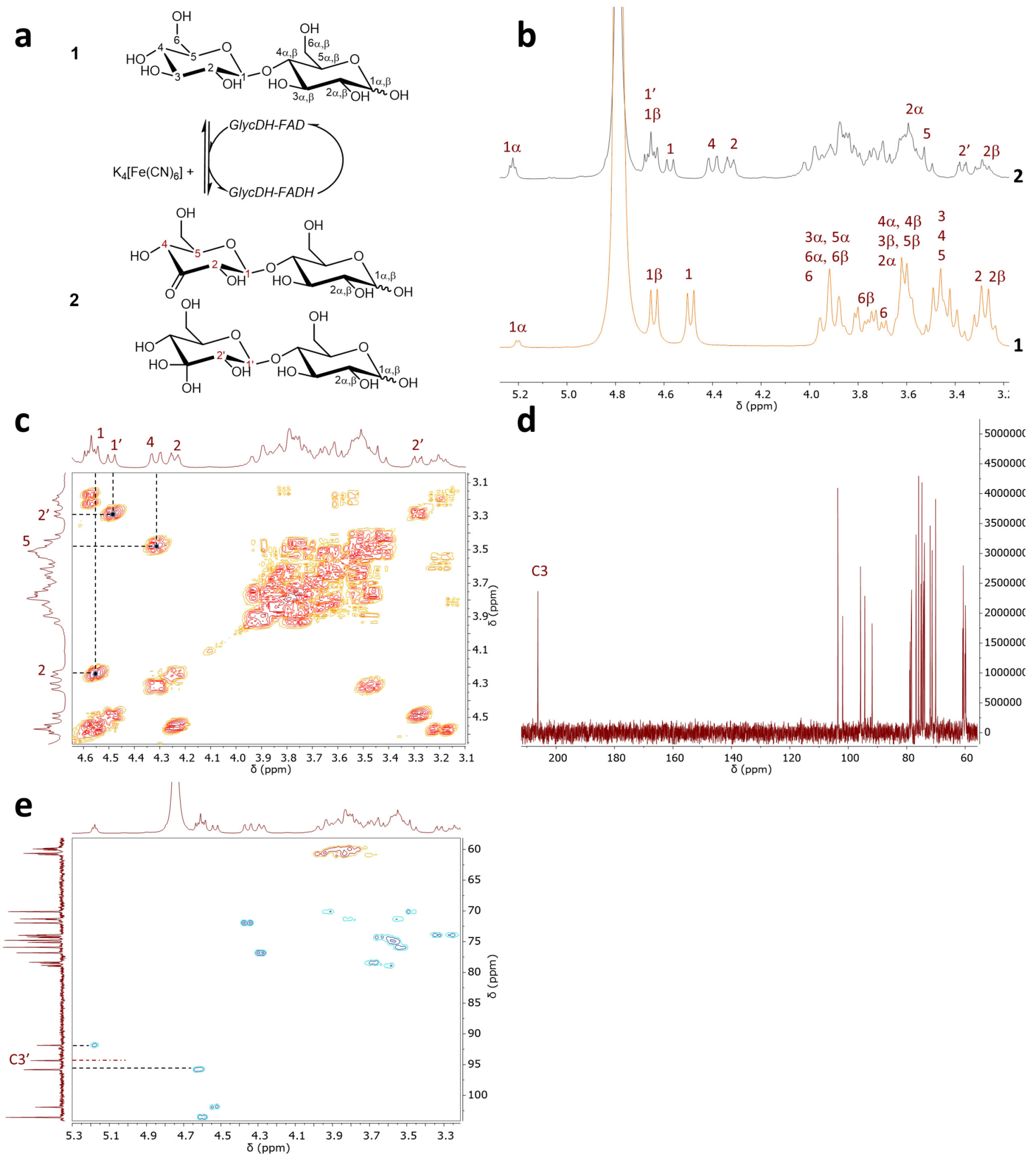

Supplementary Figure 50. Product identification from the GlycDH-catalyzed reaction with cellobiose. **a.** Reaction scheme for the conversion of cellobiose (**1**) into 3'-keto-cellobiose (**2**, present partly (55%) in the hydrated (gem-diol) form). **b.** Stacked substrate (**1**) and product (**2**) ^{1}H NMR spectra presenting the absence of H-3 signal in **2**, indicating full conversion of cellobiose into 3'-keto-cellobiose. The signals from H-1, H-2 and H-4 are shifted downfield due to the deshielding effect from the C3 keto-moiety. **c.** COSY analysis of 3'-keto-cellobiose showing the relevant coupling interactions with dashed lines. No cross peak is observed for H-2, confirming the oxidation at C3. **d.** ^{13}C NMR spectrum showing the expected chemical shift of a carbonyl carbon (~208 ppm) due to oxidation at C3. **e.** HSQC analysis of 3'-keto-cellobiose confirming the partial presence of gem-diol at C3' (~95 ppm, no proton-carbon bond correlation observed). The NMR experiments were performed in D_2O (^{1}H NMR, 300 MHz; ^{13}C NMR, 126 MHz).

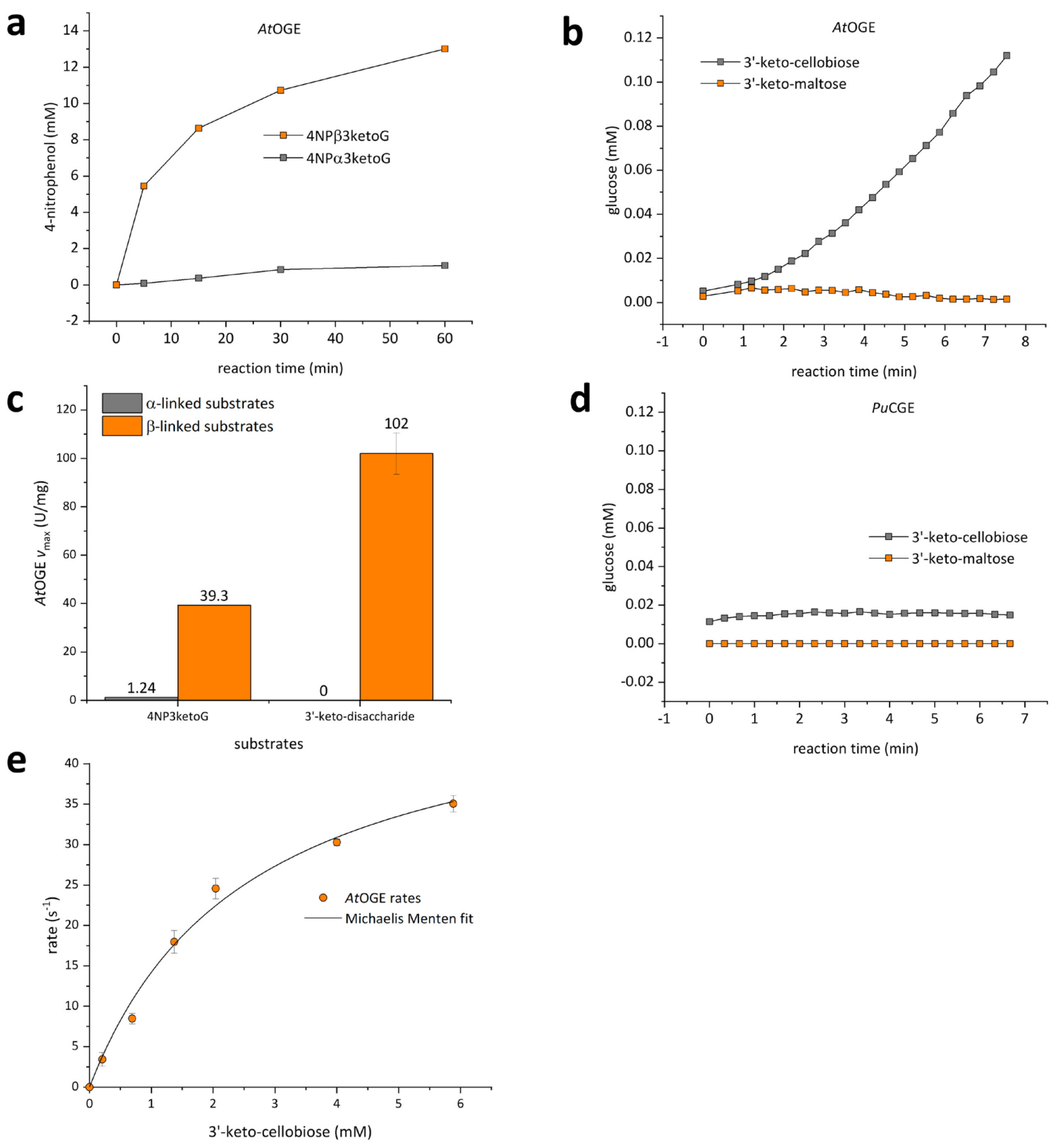

Supplementary Figure 51. Stereochemical preference of *At*OGE and *Pu*CGE analyzed with different α- and β-configured substrates. **a.** Time courses of *At*OGE catalyzed elimination of 4NPα3ketoG and 4NPβ3ketoG. **b.** Time courses of *At*OGE or **d.** *Pu*CGE catalyzed conversion of 3'-keto-maltose and 3'-keto-cellobiose. **c.** Specific activities of *At*OGE with α- and β-linked *O*-glycosides. **e.** Kinetic analysis of *At*OGE (3.00 × 10^{-4} mg/mL) and 3'-keto-cellobiose (0.2–6.0 mM). For 4NP-substrates (16 mM), initial rates were determined in potassium phosphate buffer (10 mM, pH 6.0) with 0.03 mg/mL of *At*OGE. Reactions with keto-disaccharide substrates were performed in hexokinase assay solution (100 mM PIPES (pH 7.6), 4.7 mM ATP, 3.1 mM NAD^+, 4.9 mM $MgCl_2$, 1.5 U/mL hexokinase, 1.5 U/mL glucose 6-phosphate dehydrogenase) with 3.00 × 10^{-4} mg/mL *At*OGE or 1.20 mg/mL *Pu*CGE. Reactions in **b** and **d** were performed with 2.0 mM 3-keto-dissacharide. All reactions were incubated at 37 °C and 650 rpm agitation. Initial rates were fitted to the Michaelis Menten (equation 2) using

Origin software. The fit is shown as a solid line with S.D. indicated by error bars. For further experimental details and for the analytical procedures used, see the Methods section of the main manuscript. Source data are provided as a Source Data file. (n = 1 individual experiment for **a**, 2 individual experiments for **d** and **e**, and 3 individual experiments for **b**).

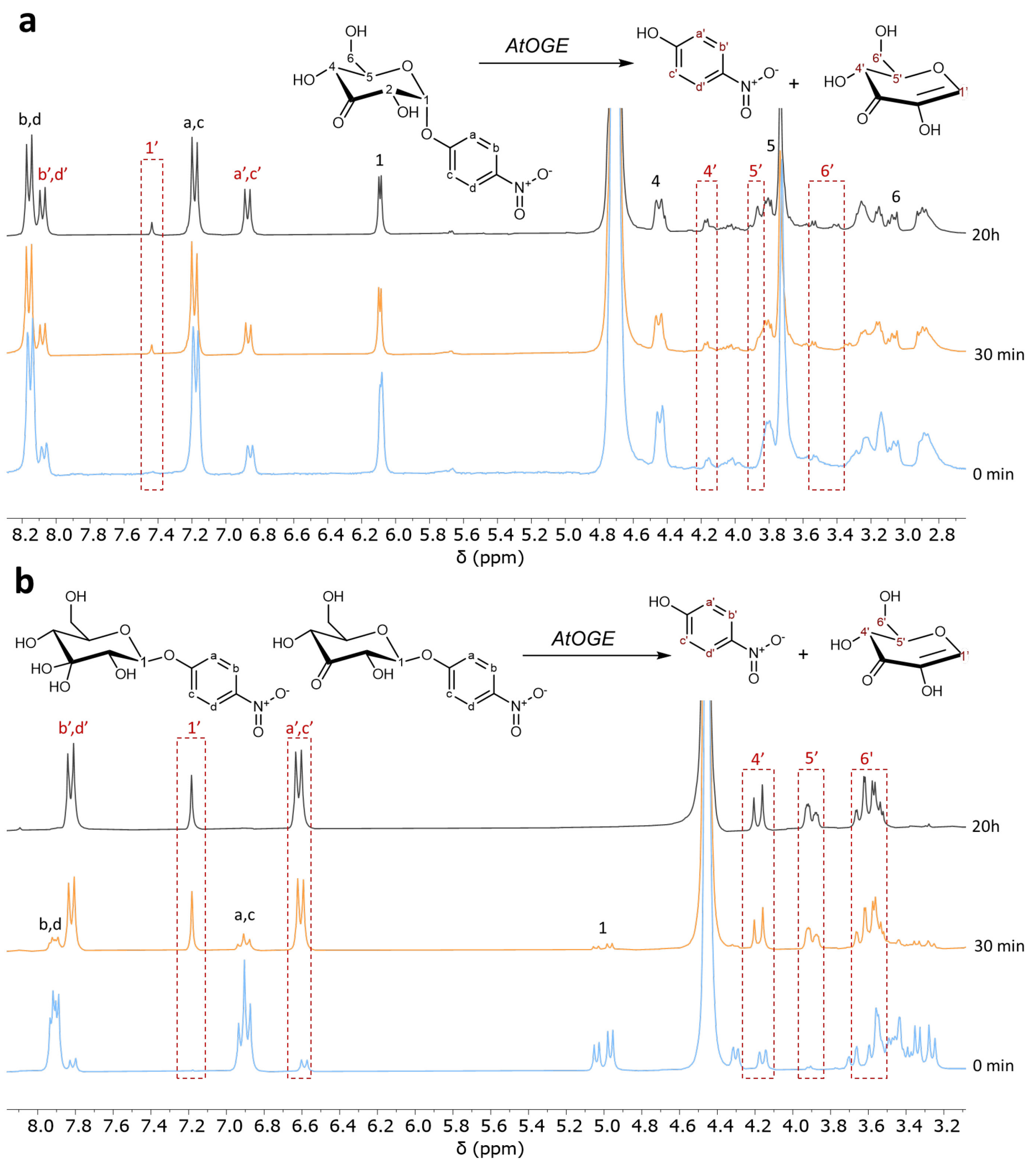

Supplementary Figure 52. ^{1}H NMR spectra of *At*OGE-catalyzed elimination of 4NPα3ketoG (**a**) and 4NPβ3ketoG **(b).** Starting substrate for **b** is a 3-keto and 3-hydrated (gem-diol) mixture. Substrate (15 mM) was dissolved in potassium phosphate buffer (10 mM, pD 6.9) and measured before, 30 min and 20 h after enzyme administration (0.04 mg/mL). Between measurements (300 MHz), the sample was incubated at 37 °C.

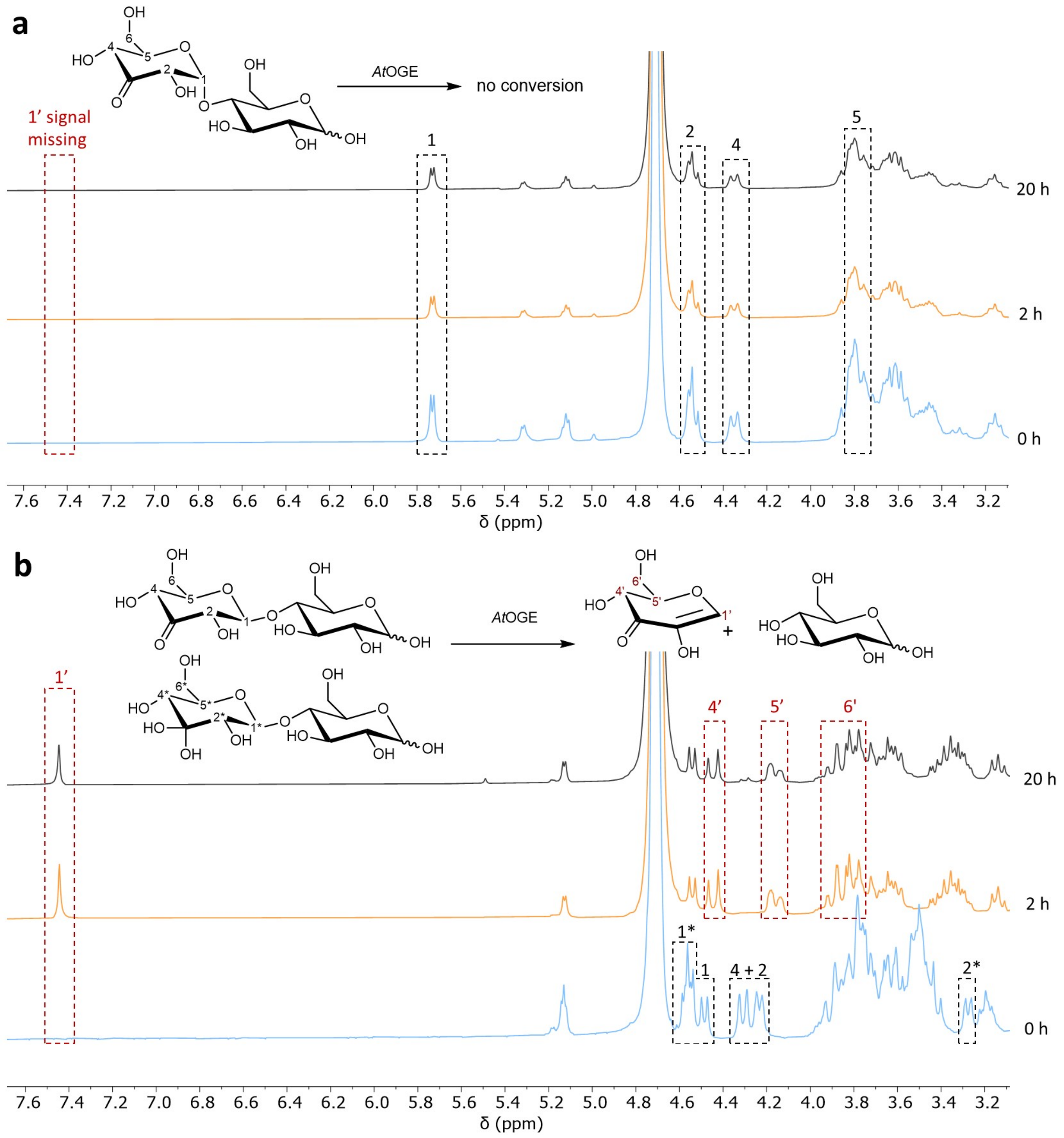

Supplementary Figure 53. ^{1}H NMR spectra of *At*OGE-catalyzed elimination of 3'-ketomaltose (**a**) and 3'-keto-cellobiose **(b).** Starting substrate for **b** is a 3-keto and 3-hydrated (gem-diol) mixture. Substrate (15 mM) was dissolved in potassium phosphate buffer (10 mM, pD 6.9) and measured before, 2 h and 20 h after *At*OGE administration (0.16 mg/mL). Between measurements (300 MHz), the sample was incubated at 37 °C.

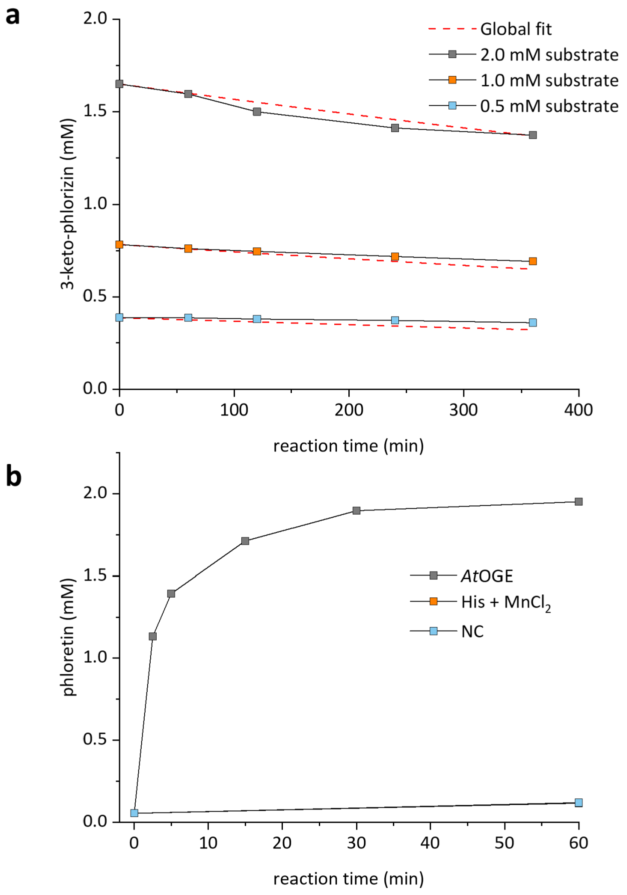

Supplementary Figure 54. Spontaneous deglycosylation of 3-keto-phlorizin based on phloretin release. **a.** 3-keto-phlorizin degradation at different starting concentrations with global fitting of the data with Equation 1 in main text (exponential fit) for determination of rate constant. **b.** Time course of phloretin release with the addition of free catalytic groups compared to *At*OGE as positive control (PC). For all reactions (n = 1 individual experiment), 3-keto-phlorizin (0.5–2.0 mM) was dissolved in HEPES buffer (50 mM; pH 6.5) to avoid precipitation of added $MnCl_2$, incubated at 37 °C for 4 h under agitation (650 rpm), quenched with MeCN and analyzed on HPLC. *At*OGE (5.00 × 10^{-3} mg/mL; 0.2 µM) was used as a positive control and 1000-fold equimolar amounts of histidine and $MnCl_2$ (167 µM) were added to the spontaneous degradation experiment (**b**). No enzyme or free catalytic group was added to the negative control (NC), which overlaps with the His + $MnCl_2$ graph. Sigma plot 10.0 was used to fit the rate constant for spontaneous elimination (k) using the time courses shown in **a**. k = -8.6 ± 1.5 × 10^{-6} s^{-1} (95% confidence interval). For further experimental details and for the analytical procedures used, see the Methods section of the main manuscript. Source data are provided as a Source Data file.

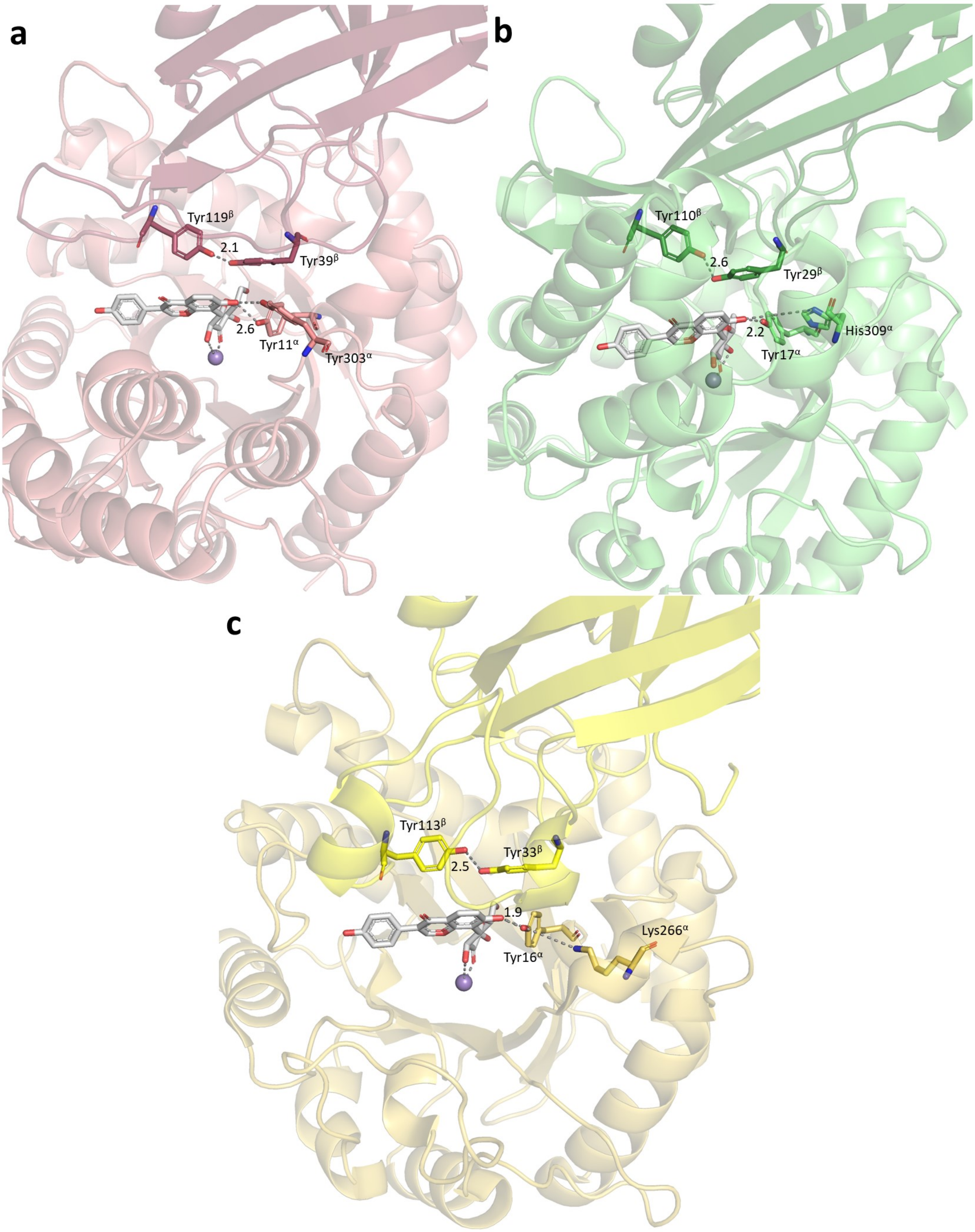

Supplementary Figure 55. Conserved Tyr residues among CGEs. CGEs show conserved active site/substrate coordinating Tyr residues in both subunits. CGEs from human gut bacterial strain PUE[8] (**a**), *A. globiformis*[8] (**b**) and *E. cellulosolvens*[8] (**c**) were compared. Docking from 7exz and 3-keto-puerarin was used and superimposed with *Ag*CGE2 and *Eu*CGE. Substrate 3-keto-puerarin in grey and Mn^{2+} in purple.

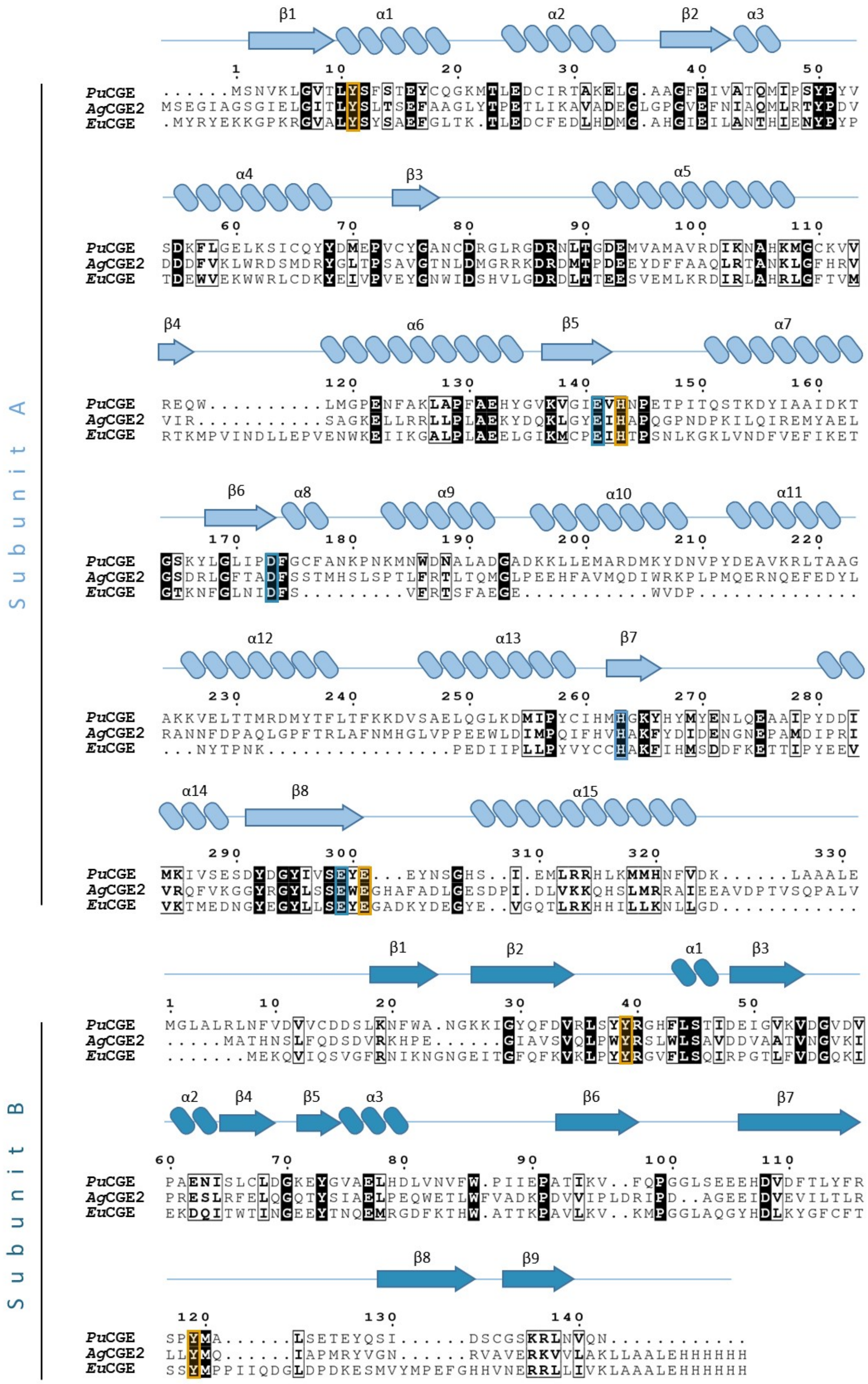

Supplementary Figure 56. Sequence alignment of CGEs showing conserved residues and secondary structures. Mn^{2+} coordinating residues highlighted in blue, conserved Tyr and other catalytic residues in ochre. Sequences were aligned using the Muscle alignment and processed in the ESPript 3 (https://espript.ibcp.fr/ESPript/ESPript/index.php) online tool.

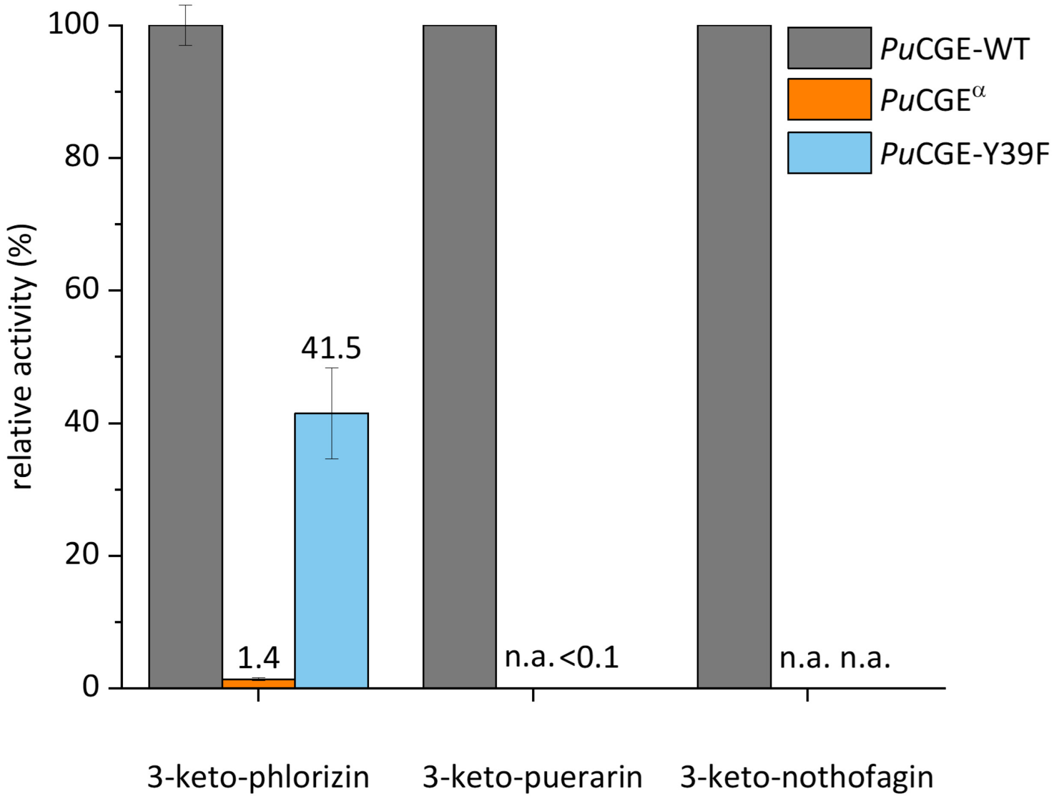

Supplementary Figure 57. Relative activity of *Pu*CGE variants with *O*- and *C*-glycosides compared to the wild-type (WT). Relative activities were determined by dividing the specific activity of reactions by WT enzyme rates. Substrates (1.5 mM) were dissolved in potassium phosphate buffer (10 mM, pH 6.0, 5.0% (v/v) DMSO) and incubated at 37 °C under agitation (650 rpm). For 3-keto-phlorizin, 3-keto-puerarin and 3-keto-nothofagin (both, n = 1 individual experiment) 0.10, 0.05 and 0.10 mg/mL *Pu*CGE-WT were added, respectively. Variants *Pu*CGE$^{\alpha}$ and *Pu*CGE-Y39F were used at a concentration of 0.50 mg/mL. Samples were quenched with MeCN and analyzed on HPLC. n.a. = no activity determined. For further experimental details and for the analytical procedures used, see the Methods section of the main manuscript. Source data are provided as a Source Data file. (n = 1 individual experiment for *C*-glycoside substrates; n = 3 individual experiments for *O*-glycoside substrate).

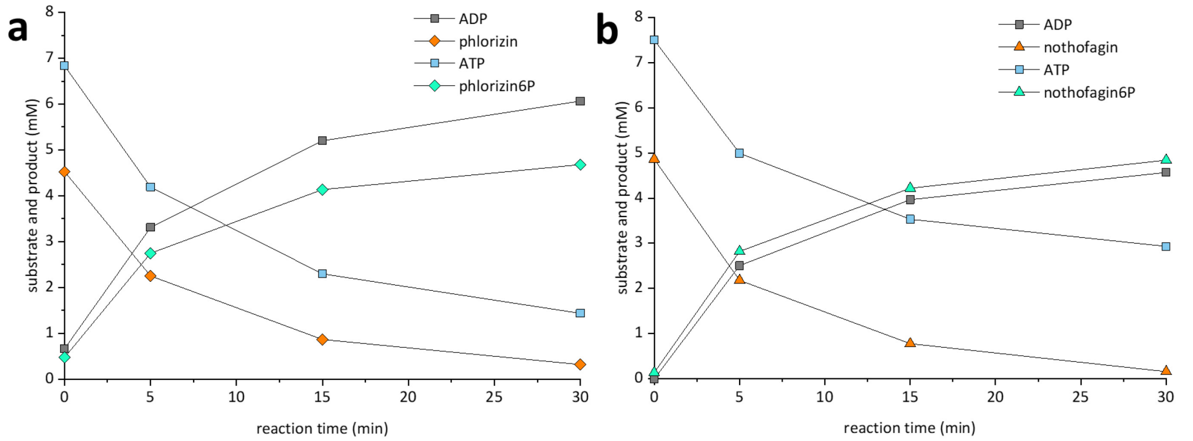

Supplementary Figure 58. The 6-phospho-β-glucoside kinase from *Klebsiella pneumoniae* (BglK)[11] catalyzed phosphorylation of phlorizin (**a**) and nothofagin (**b**). Typically, 5.0 mM substrates, 1.0 mM $MgCl_2$ and 1.5-fold mM of ATP in respect to substrate concentration were dissolved in 10% (v/v) DMSO and 50 mM HEPES buffer pH 6.5. BglK was used at 1.00 mg/mL and reactions were incubated at 37 °C and 650 rpm agitation. Reactions (n = 1 individual experiment) were quenched in MeCN and analyzed on HPLC**.** Phosphorylated product is shown as substrate6P. For further experimental details and for the analytical procedures used, see the Methods section of the main manuscript. Source data are provided as a Source Data file.

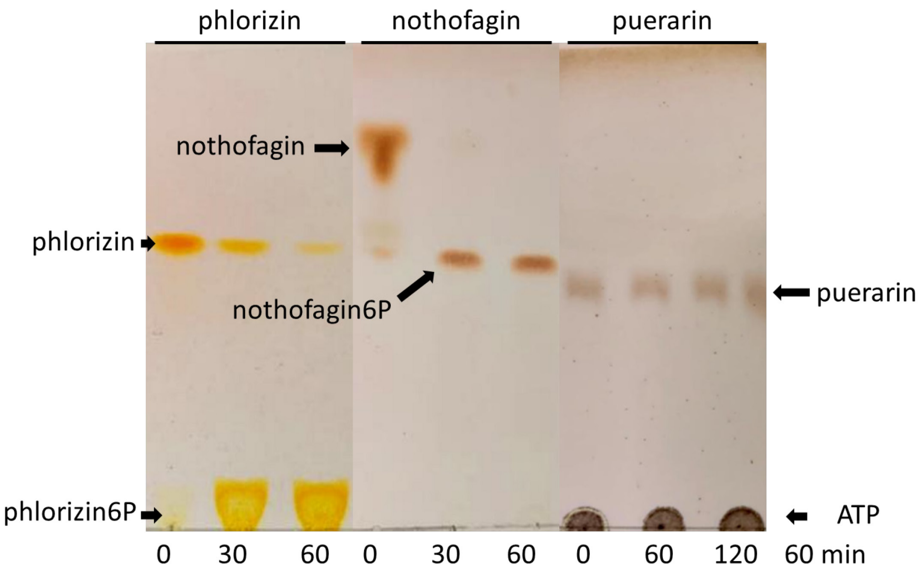

Supplementary Figure 59. TLC analysis of BglK-catalyzed phosphorylation of various substrates. For chromatograms of phlorizin and puerarin a mobile phase of 1:9 MeCN and dH_2O was used, for nothofagin 2:1:1 of 1-butanol, acetic acid and dH_2O. TLC plates detecting phlorizin were developed with *p*-anisaldehyde stain, all other TLC plates were developed using a thymol stain. Reaction parameters as described in Figure S58 were used (n = 1 individual experiment). Source data are provided as a Source Data file.

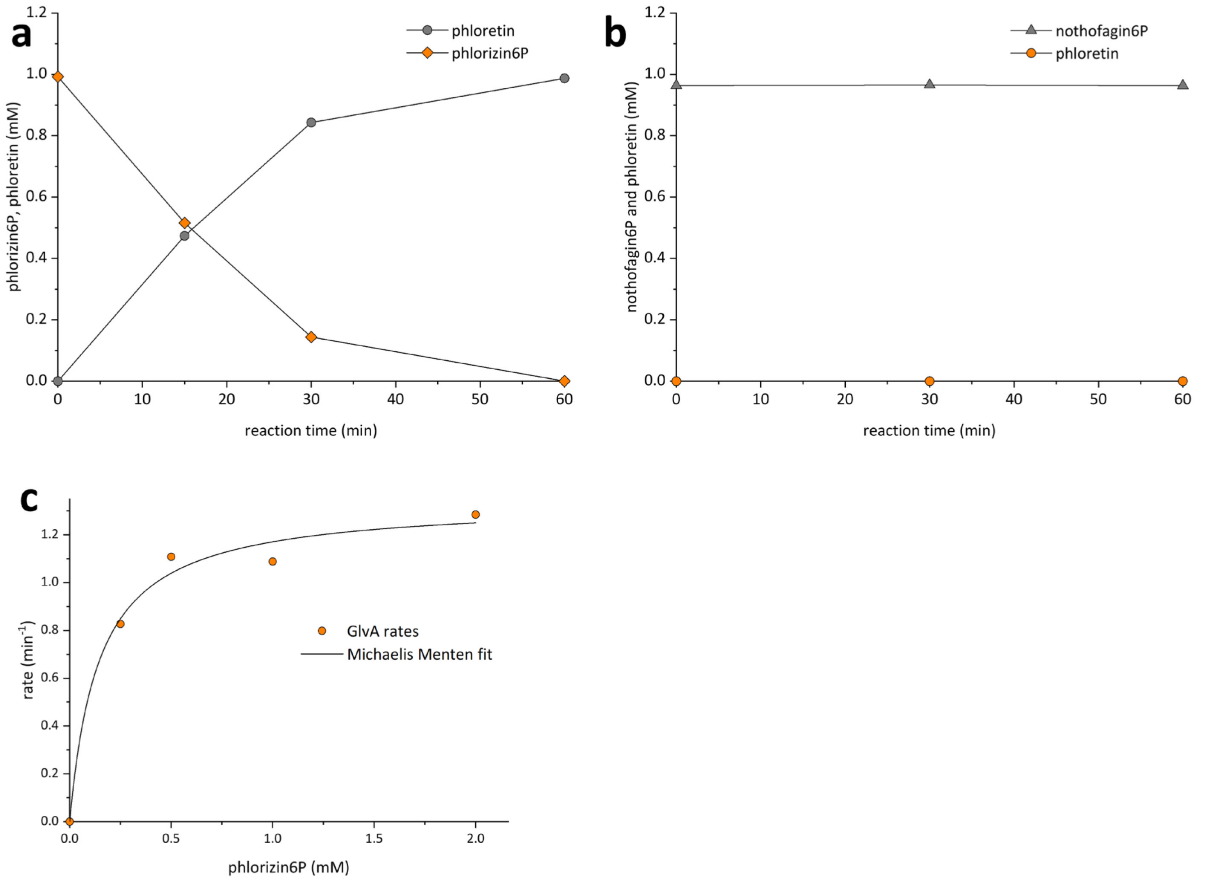

Supplementary Figure 60. Time courses of GlvA-catalyzed reactions and activity parameters associated with the reactions. **a.** Phlorizin-6-phosphate (phlorizin6P) **b.** Nothofagin-6-phosphate (nothofagin6P). **c.** Kinetic analysis of GlvA with phlorizin6P. A substrate range of 0.0–2.0 mM was tested. Reactions were conducted in 50 mM HEPES pH 7.5 (nothofagin6P in pH 6.5) buffer with 0.5 mM $MnSO_4$ and 0.3 mM NAD^+. The reactions were initiated with the addition of GlvA (for **a** and **b** 2.00, for **c** 1.00 mg/mL) at 37 °C. Reactions were quenched with MeCN and analyzed on HPLC (n = 1 individual experiment). Initial rates were fitted to the Michaelis Menten (equation 2) using Origin software. The fit is shown as a solid line. For further experimental details and for the analytical procedures used, see the Methods section of the main manuscript. Source data are provided as a Source Data file.

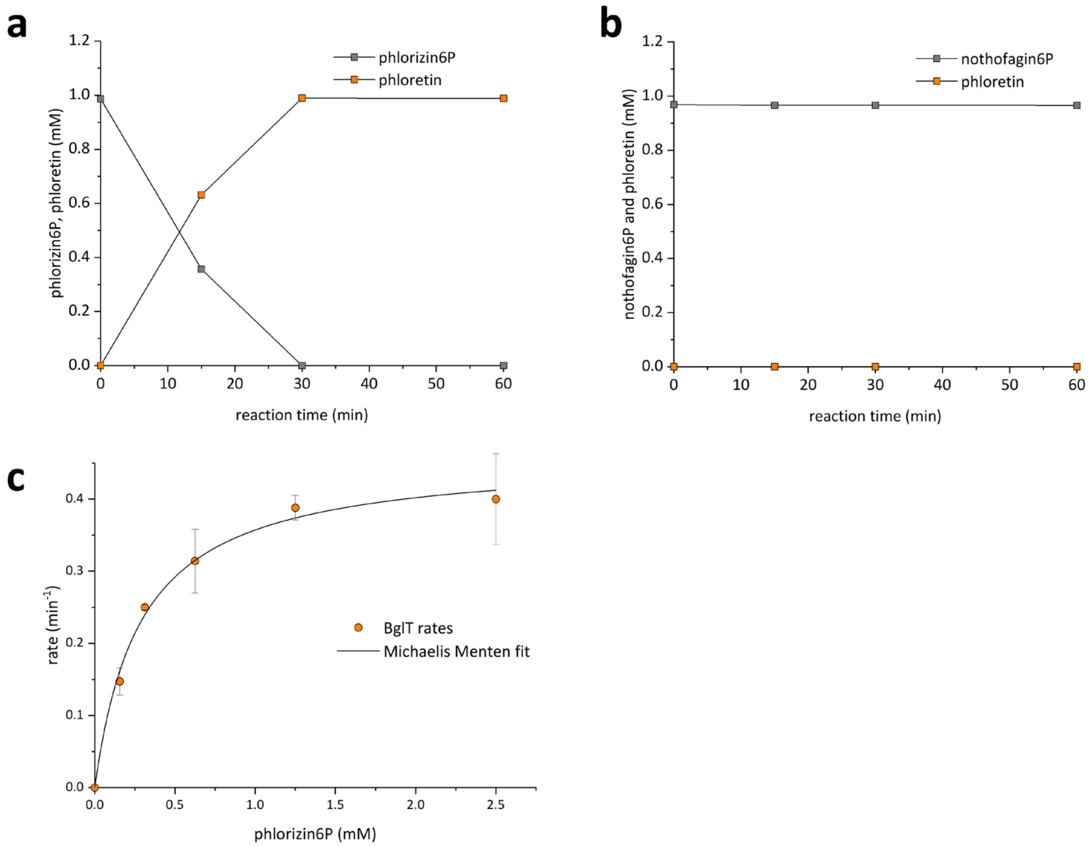

Supplementary Figure 61. Time courses of BglT[12]-catalyzed reactions and activity parameters associated with the reactions. **a.** Phlorizin-6-phosphate (phlorizin6P) **b.** Nothofagin-6-phosphate (nothofagin6P). **c.** Kinetic analysis of BglT with phlorizin6P. A substrate range of 0.1–2.5 mM was tested. Reactions were conducted in 50 mM HEPES pH 7.5 (nothofagin6P in pH 6.5) buffer with 0.5 mM $MnSO_4$ and 0.3 mM NAD^+. The reactions were started with the addition of BglT (2.00 for **a** and **b**, 1.00 mg/mL for **d**) at 37 °C. Reactions were quenched with MeCN and analyzed on HPLC (n = 1 individual experiment in **a** and **b**, n = 3 individual experiments in **c**). Initial rates were fitted to the Michaelis Menten (Equation 2, main text) using Origin software. The fit is shown as a solid line with S.D. indicated by error bars. For further experimental details and for the analytical procedures used, see the Methods section of the main manuscript. Source data are provided as a Source Data file.

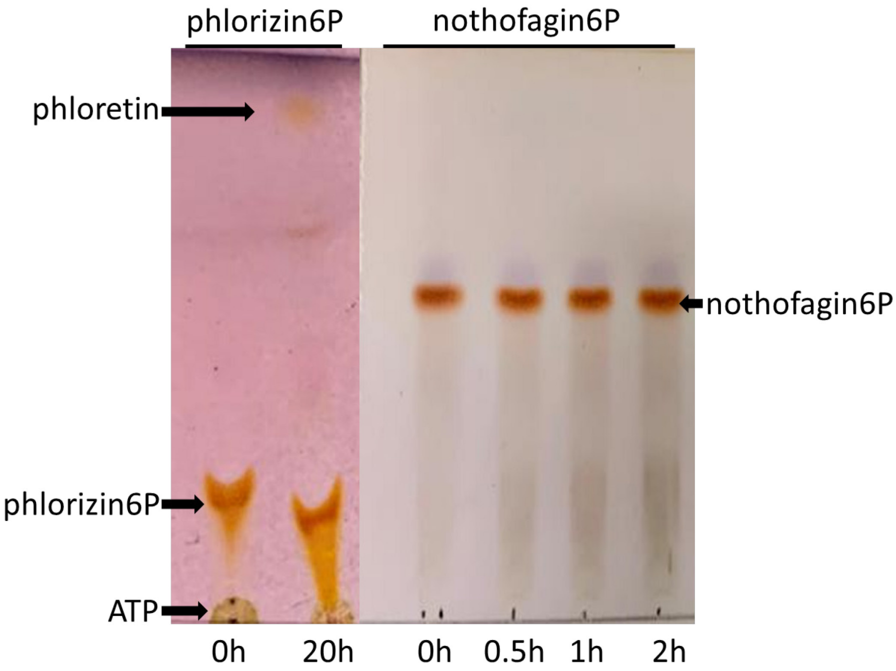

Supplementary Figure 62. TLC analysis of GlvA-catalyzed deglycosylation of phlorizin6P and nothofagin6P. Compounds from the phlorizin6P reaction were separated using a 9:1 mobile phase of MeCN and dH_2O. A *p*-anisaldehyde based stain was used. Compounds from the nothofagin6P reaction were separated using a 2:1:1 mobile phase of 1-butanol, acetic acid and dH_2O. A thymol-based stain was used. Same reaction parameters as described in Figure 60 were used (n = 1 individual experiment). Source data are provided as a Source Data file.

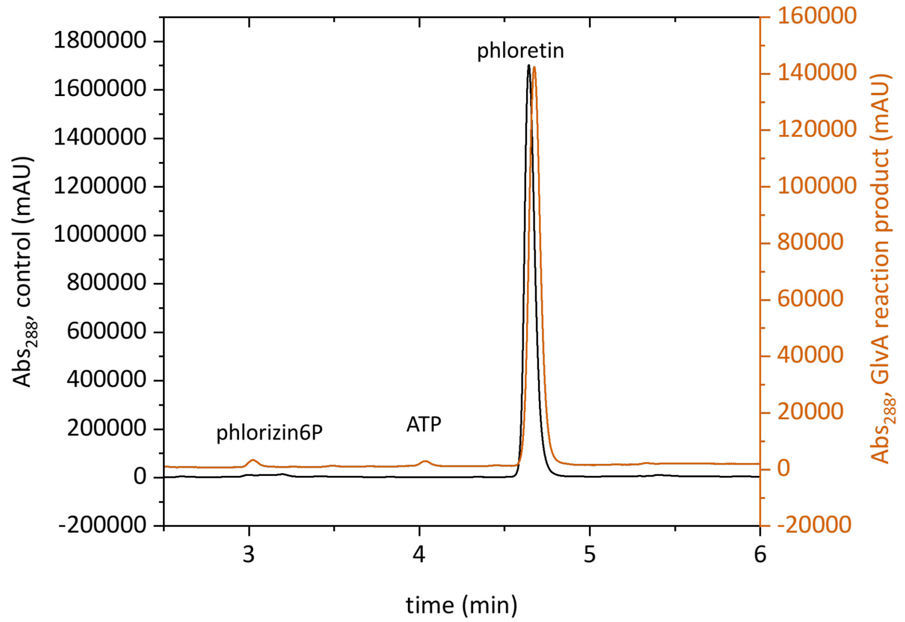

Supplementary Figure 63. HPLC chromatogram of GlvA-catalyzed deglycosylation of phlorizin6P, based on measurement of aglycone release (n = 1 individual experiment). The GlvA reaction sample (orange) is superimposed on phloretin control sample (black). Isolated phlorizin6P was dissolved in 50 mM HEPES buffer (pH 7.5) with 0.5 mM $MnSO_4$ and 0.3 mM NAD^+. The reaction was started using GlvA (2.00 mg/mL) at 37 °C, quenched with MeCN and analyzed on HPLC. Besides the aglycone product phloretin, small residues from the starting substrate and ATP were detected.

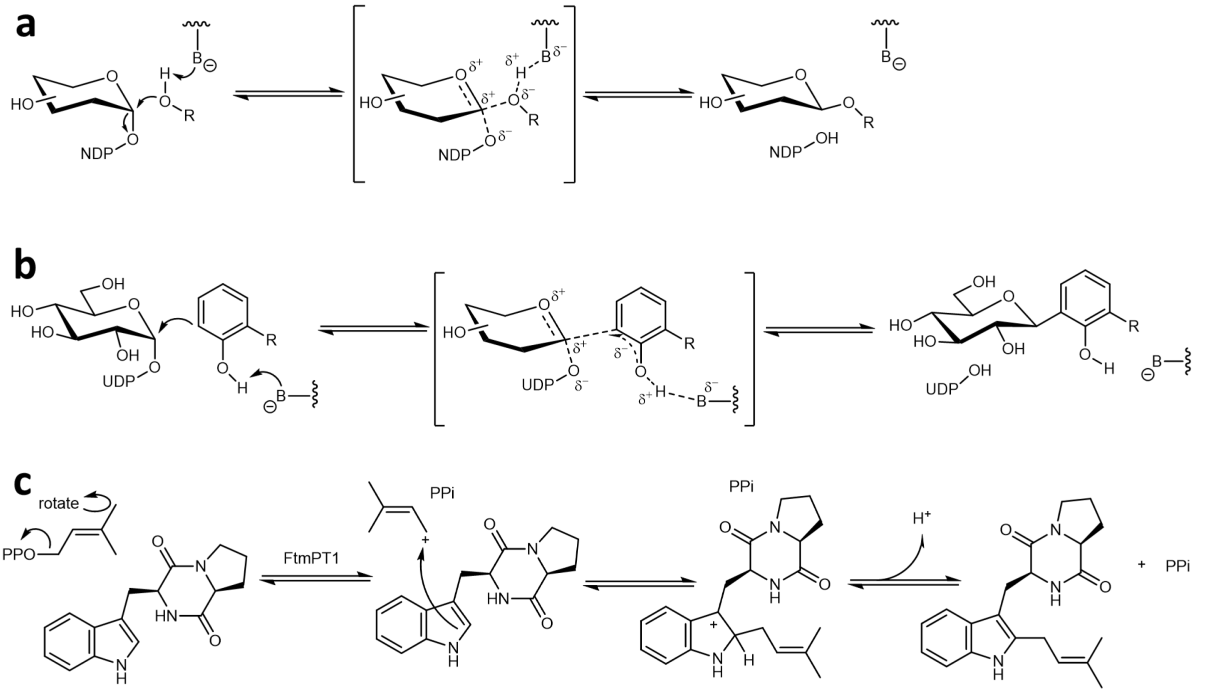

Supplementary Figure 64. Proposed mechanisms of glycosyltransferase and alkyltransferase reactions. **a.** General reaction of an inverting *O*-glycosyltransferase.[13] **b.** Proposed reaction of an aromatic *C*-glycosyltransferase.[13] **c.** Proposed reaction of the *C*-prenyltransferase FtmPT1[14] representing alkyltransferases. B = general base catalyst.

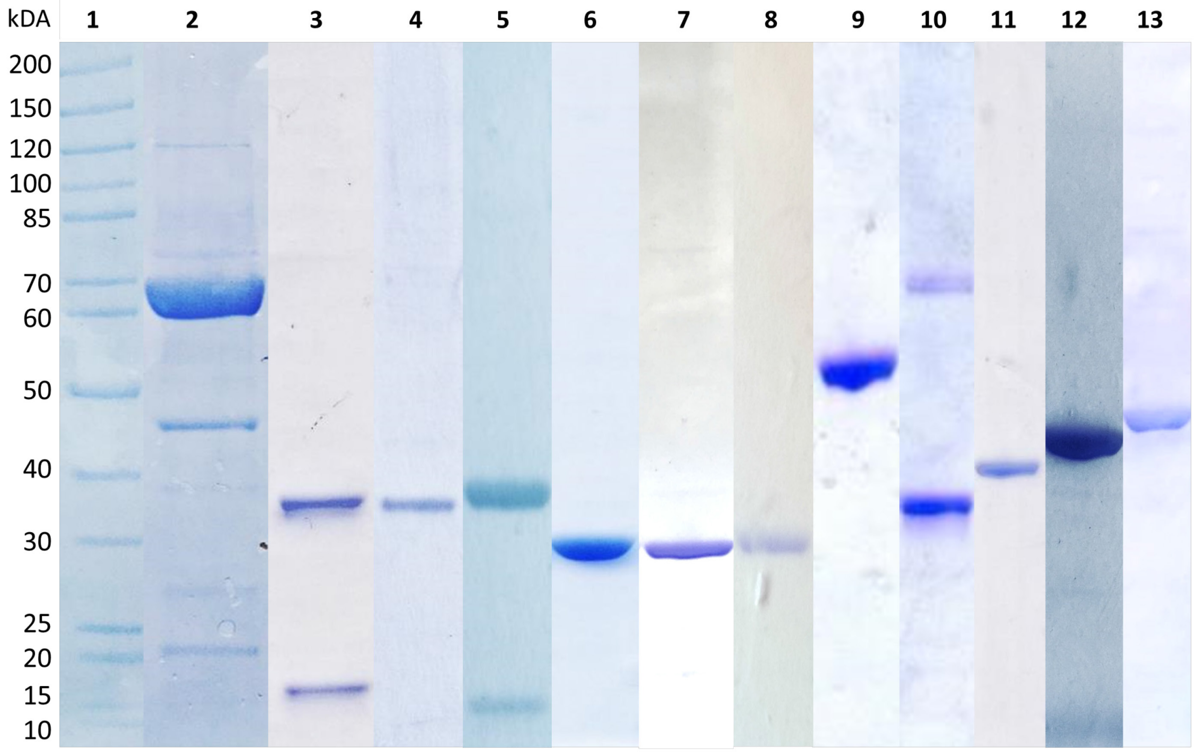

Supplementary Figure 65. SDS polyacrylamide gel showing the purified enzymes used in this study and their theoretical subunit molecular mass in parenthesis. **1:** protein size standard. **2:** GlycDH (66.3 kDa) and twin-arginine translocation pathway (TAT) signal protein (20.3 kDa). **3:** *Pu*CGE; monomer α (39.2 kDa) and β (18.3 kDa). **4:** *Pu*CGE$^{\alpha}$ (39.2 kDa). **5:** *Pu*CGE-Y39F$^{\beta}$; monomer α (39.2 kDa) and β (18.3 kDa). **6:** *At*OGE (29.9 kDa). **7:** *At*OGE-H134A (29.9 kDa). **8:** *At*OGE-H189A (29.9 kDa). **9:** GH4 family enzyme GlvA (52.1 kDa). **10:** BglK (34.9 kDa). **11:** *At*HYD (41.0 kDa). **12:** Gfo Oxo from *A. tumefaciens* (43.6 kDa). **13:** GH4 family enzyme BglT (49.0 kDa). Source data are provided as a Source Data file.

Supplementary Tables

Supplementary Table 1. Activity parameters of GlycDH for oxidation of different substrates (n = 1 individual experiment). Initial rates were determined at 2.0 mM substrate concentration. [1)]Rates determined from the sum of oxidized 3-keto- and further isomerized 2-keto-product; [2)]Apparent k_{cat} calculated from the specific activity. [3)]Reaction with O_2 instead of $K_3[Fe(CN)_6]$ as electron acceptor. [4)]mM isomerized per minute from 3-keto- to 2-keto-glucoside. [5)]Percentage of 2-keto-glucoside within converted product. For experimental details and the analytical procedures used, see the Methods section Synthesis and isolation of keto-glycosides.

Substrate	Specific activity (U/mg)	k_{cat} (s^{-1})[2)]	Conversion (%) / h [2.0 mM starting substrate]	Oxidized position
Phlorizin	5.25 *(0.01)*[3)]	5.80	100	C3
Puerarin[1)]	0.43	0.47	73	C2/C3
Nothofagin *(pH 5.7)*[1)]	1.86	2.05	93	C2/C3
	(0.02 mM/min isomerization)[4)]	-	*(11% 2-keto-form)*[5)]	
Nothofagin *(pH 7)*[1)]	3.78	4.18	100	C2/C3
	(0.30 mM/min isomerization)[4)]	-	*(85% 2-keto-form)*[5)]	

Supplementary Table 2. Crystallographic data collection and refinement statistics of *At*OGE. Statistics for the highest-resolution shell are shown in parentheses.

	***At*OGE**
Wavelength (Å)	1.033
Resolution range (Å)	46.83 - 1.995 (2.067 - 1.995)
Space group	P 2_1 2_1 2_1
Unit cell (Å,°)	36.58, 93.67, 123.68; 90 90 90
Total reflections	368496 (32392)
Unique reflections	29564 (2699)
Multiplicity	12.5 (12.0)
Completeness (%)	99.03 (91.99)
Mean I/sigma(I)	20.30 (6.24)
Wilson B-factor	28.81
R-merge	0.0794 (0.2910)
R-meas	0.0829 (0.3038)
R-pim	0.0234 (0.0859)
CC1/2	0.999 (0.985)
CC*	1 (0.996)
Reflections used in refinement	29542 (2698)
Reflections used for R-free	1124 (103)
R-work	0.1742 (0.2052)
R-free	0.2175 (0.2745)
CC(work)	0.972 (0.911)
CC(free)	0.939 (0.852)
Number of non-hydrogen atoms	3644
macromolecules	3339
ligands	3
solvent	302
Protein residues	439
RMS(bonds)	0.006
RMS(angles)	0.80
Ramachandran favored (%)	97.90
Ramachandran allowed (%)	2.10
Ramachandran outliers (%)	0.00
Rotamer outliers (%)	0.00
Clashscore	3.14
Average B-factor	34.22
macromolecules	34.05
ligands	39.08
solvent	36.05

Supplementary Table 3. RMSD values of *At*OGE monomer β superimposed with monomer α and CGEs and sequence alignment with CGEs. [1)]Root mean square deviation of atomic positions. [2)]Superposition of *At*OGE monomer β and *Pu*CGE subunit α TIM-barrel central fold. Calculated in PyMOL; sequence similarity/identity was calculated with EMBOSS Needle online tool (https://www.ebi.ac.uk/Tools/psa/emboss_needle/)

Superimposed enzyme	Entry ID	RMSD[1)]-value	Sequence similarity	Sequence identity
*At*OGE (subunit α)	8BVK	0.221	-	-
*Pu*CGE (subunit α)	7exz	2.775	27.1	15.5
	with TIM-barrel fold: 1.404[2)]			
*Ag*CGE2 (subunit α)	7dnn	2.585	22.2	13.4
*Eu*CGE (subunit α)	7exb	2.853	5.70	3.90

Supplementary Table 4. Activity parameters of deglycosylating enzyme systems compared. Standard deviation n = 3, otherwise n = 1; n.a. = no activity; -= not determined; Specific activity was determined at substrate saturation. [1)]Conversion after 24 h of incubation with 2.0 mM starting substrate, unless stated otherwise. [2)]*Pu*CGE was pre-incubated with 1.0 and *At*OGE with 10 mM EDTA, both for 120 min at 4.0 °C. [3)]Apparent k_{cat} was calculated from specific activity and enzyme concentration. [4)]Phlorizin6P was used as substrate. [5)]Parameter was obtained from a one-pot reaction, as described in **Figure S37d**. [6)]Initial rates determined at 2.0 mM (3-keto-nothofagin) and 16 mM substrate (*p*NPα3ketoG and *p*NPβ3ketoG). For experimental details and the analytical procedures used, see the respective Methods sections.

Substrate	Enzyme	Specific activity (U/mg)	Conversion (%)[1)]	k_{cat} (s^{-1})	K_M (mM)
3-keto-phlorizin 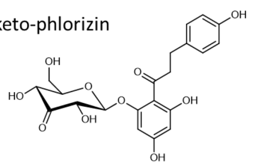	***Pu*CGE**	1.25 ± 0.05	>90	1.20 ± 0.05	0.6 ± 0.1
	w/ EDTA[2)]	0.01	3.0	0.01[3)]	
	***At*OGE**	235 ± 5.02	100	117 ± 2.50	0.1 ± <0.1
	w/ EDTA[2)]	3.14	10	1.57[3)]	
	**GlvA**[4)]	0.03 ± <0.01	>90	0.02 ± <0.01	0.1 ± <0.1
	**BglT**[4)]	0.01 ± <0.01	>90	0.01 ± <0.01	0.3 ± <0.1
3-keto-puerarin 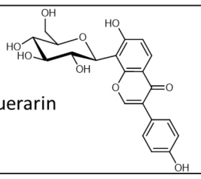	***Pu*CGE**	18.6 ± 0.41	>90[5)]	17.9 ± 0.39	0.8 ± <0.1
	w/ EDTA[2)]	n.a.	n.a.	-	-
	***At*OGE**	n.a.	n.a.	-	-
	w/ EDTA[2)]	-	-		
3-keto-nothofagin 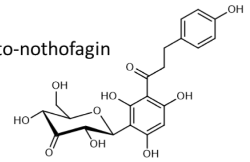	***Pu*CGE**	0.64[6)]	27	0.61[3)]	-
	w/ EDTA[2)]	n.a.	n.a.	-	
	***At*OGE**	n.a.	n.a.	-	-
	w/ EDTA[2)]	-	-		
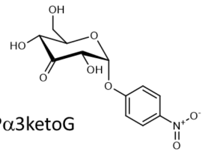 *p*NPα3ketoG	***At*OGE**	1.24[6)]	8.0 (16 mM)	0.62[3)]	-
*p*NPβ3ketoG 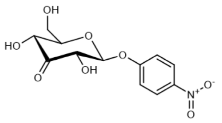	***At*OGE**	39.3[6)]	86 (16 mM)	19.6[3)]	-
3-ketomaltose 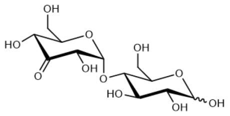	***At*OGE**	n.a.	n.a.	-	-
	***Pu*CGE**	n.a.	n.a.	-	-
3-ketocellobiose 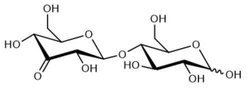	***At*OGE**	102 ± 8.26	100	50.8 ± 4.14	2.6 ± 0.1
	***Pu*CGE**	n.a.	n.a.	-	-

Supplementary Table 5. Activity of *Pu*CGE variants with *O*- and *C*-glycosides compared to the wild-type form. Initial rates determined at 1.5 mM substrate concentration. Values with standard deviation n=3, otherwise n=1. Parameters were calculated from reactions described in Figure S57. For experimental details and the analytical procedures used, see the respective Methods sections.

Substrate	Enzyme	*U/mg*	Activity relative to DgpBC (%)[1)]
Phlorizin	*Pu*CGE-WT	0.91 ± 0.03	100
	*Pu*CGE$^{\alpha}$	0.01 ± <0.01	1.4
	*Pu*CGE-Y39F	0.38 ± 0.06	42
Puerarin	*Pu*CGE-WT	18.6	100
	*Pu*CGE$^{\alpha}$	n.a.	n.a.
	*Pu*CGE-Y39F	<0.01	<0.1
Nothofagin	*Pu*CGE-WT	0.49	100
	*Pu*CGE$^{\alpha}$	n.a.	n.a.
	*Pu*CGE-Y39F	n.a.	n.a.

Supplementary Table 6. Sequences of enzymes.

**GlycDH** (with Strep-tag and TEV restriction site)	
Nucleotide sequence	1 ATGGTACATA TGTGGAGCCA TCCGCAGTTC GAGAAGGAAA ACCTGTATTT TCAGGGCAGC<br>61 GCCGGCGCGG ATCCGATGGC GAACAATCAT TACGATGCGA TTGTGGTTGG TAGCGGTATT<br>121 AGCGGTGGTT GGGCGGCGAA AGAACTGACC CAGAAGGGCC TGAAAGTGCT GCTGCTGGAA<br>181 CGTGGTCGTA ACATCGAGCA CATTACCGAT TACCAGAACG CGGACAAGGA AGCGTGGGAT<br>241 TATCCGCACC GTAACCGTGC GACCCAAGAG ATGAAAGCGA AGTACCCGGT TCTGAGCCGT<br>301 GACTATCTGC TGGAGGAAGC GACCCTGGGC ATGTGGGCGG ATGAGCAGGA AACCCCGTAC<br>361 GTGGAGGAAA AACGTTTCGA CTGGTTTCGT GGTTATCATG TTGGTGGCCG TAGCCTGCTG<br>421 TGGGGCCGTC AGACCTACCG TTGGAGCCAA ACCGACTTCG AGGCGAACGC GAAAGAAGGT<br>481 ATCGCGGTGG ATTGGCCGAT TCGTTATCAA GACGTGGCGC CGTGGTACGA TTATGTTGAA<br>541 CGTTTCGCGG GTATCAGCGG CAGCAAGGAA GGTCTGGACA TTCTGCCGGA TGGCGAGTTT<br>601 CTGCCGCCGA TCCCGCTGAA CTGCGTGGAG GAAGACGTTG CGCGTCGTCT GAAAGATCGT<br>661 TTCAAGGGTA CCCGTCACCT GATCAACAGC CGTTGCGCGA ACATTACCCA GGAGCTGCCG<br>721 GACCAAGATC GTACCCGTTG CCAGTTTCGT AACAAATGCC GTCTGGGTTG CCCGTTCGGT<br>781 GGCTACTTTA GCACCCAAAG CAGCACCCTG CCGGCGGCGG TGGCGACCGG CAACCTGACC<br>841 CTGCGTCCGT TCAGCATCGT TAAGGAAATC CTGTACGACA AGGATAAGAA AAAGGCGCGT<br>901 GGTGTTGAGA TCATTGACGC GGAAACCAAC ATGACCTACG AGTATACCGC GGATATCATT<br>961 TTTCTGAACG CGAGCACCCT GAACAGCACC TGGGTGCTGA TGAACAGCGC GACCGATGTT<br>1021 TGGGAAGGTG GCCTGGGTAG CAGCAGCGGC GAGCTGGGTC ACAACGTGAT GGATCACCAC<br>1081 TTCCGTATGG GTGCGACCGG CGAGGTTGAA GGCTTTGACG AATTCTACTT TAAGGGTCGT<br>1141 CGTCCGGCGG GCTTCTATAT CCCGCGTTTT CGTAACATTG GTGACGAGAA ACGTAAATAC<br>1201 CTGCGTGGTT TCGGTTATCA AGGCAGCGCG AGCCGTAGCC GTTGGGAGCG TGAAATCGCG<br>1261 GAAATGAACA TTGGTGCGGA CTACAAAGAT GCGCTGACCG AGCCGGGTGG CTGGACCATC<br>1321 GGTATGACCG CGTTTGGCGA AATGCTGCCG TATCACGAGA ACCGTGTGAA ACTGGACCAG<br>1381 AACAAAAAGG ATAAGTGGGG CCTGCCGGTG CTGAGCATGA ACGTTGAGCT GAAGCAAAAC<br>1441 GAACTGGACA TGCGTGAGGA TATGGTGAAC GACGCGGTTG AGATGTTCGA AGCGGTGGGT<br>1501 ATCAAAAACG TGAAACCGAC CCGTGGCAGC TACGCGCCGG GTATGGGCAT TCACGAAATG<br>1561 GGTACCGCGC GTATGGGCCG TGACCCGAAG AGCAGCGTGC TGAACGGTAA CAACCAAGTT<br>1621 TGGGACGCGC CGAACGTGTT TGTTACCGAT GGTGCGTGCA TGACCAGCGC GGCGTGCGTT<br>1681 AACCCGAGCC TGACCTACAT GGCGCTGACC GCGCGTGCGG CGGACTTTGC GGTTAGCGAA<br>1741 CTGAAGAAGG GTAATCTGTA A
Protein Sequence	1 MVHMWSHPQF EKENLYFQGS AGADPMANNH YDAIVVGSGI SGGWAAKELT QKGLKVLLLE<br>61 RGRNIEHITD YQNADKEAWD YPHRNRATQE MKAKYPVLSR DYLLEEATLG MWADEQETPY<br>121 VEEKRFDWFR GYHVGGRSLL WGRQTYRWSQ TDFEANAKEG IAVDWPIRYQ DVAPWYDYVE<br>181 RFAGISGSKE GLDILPDGEF LPPIPLNCVE EDVARRLKDR FKGTRHLINS RCANITQELP<br>241 DQDRTRCQFR NKCRLGCPFG GYFSTQSSTL PAAVATGNLT LRPFSIVKEI LYDKDKKKAR<br>301 GVEIIDAETN MTYEYTADII FLNASTLNST WVLMNSATDV WEGGLGSSSG ELGHNVMDHH<br>361 FRMGATGEVE GFDEFYFKGR RPAGFYIPRF RNIGDEKRKY LRGFGYQGSA SRSRWEREIA<br>421 EMNIGADYKD ALTEPGGWTI GMTAFGEMLP YHENRVKLDQ NKKDKWGLPV LSMNVELKQN<br>481 ELDMREDMVN DAVEMFEAVG IKNVKPTRGS YAPGMGIHEM GTARMGRDPK SSVLNGNNQV<br>541 WDAPNVFVTD GACMTSAACV NPSLTYMALT ARAADFAVSE LKKGNL
**BglK** (with Strep-tag and TEV restriction site)	
Nucleotide sequence	1 ATGTGGAGCC ACCCCCAGTT CGAGAAGGCC GGCGCCGAGA ACCTGTACTT CCAGGGCATG<br>61 AAAATTGCGG CGTTTGATAT TGGCGGCACC GCGCTGAAAA TGGGCGTGAT GGCGCGCGAT<br>121 GGCCGCCTGC TGGAAACCGC GCGCCAGAGC ATTAACGATA GCGATGGCGA TCGCATTCTG<br>181 CAGGCGATGC TGAGCTGGCT GGCGGCGCAT CCGAGCTGCG AAGGCATTGC GATTAGCGCG<br>241 CCGGGCTATA TTGATCCGCA TAGCGGCCTG ATTACCATGG GCGGCGCGAT TCGCCGCTTT<br>301 GATAACTTTG CGATGAAAAG CTGGCTGGAA ACCCGCACCG GCCTGCCGGT GAGCGTGGAA<br>361 AACGATGCGA ACTGCGTGCT GCTGGCGGAA CGCTGGCAGG GCAAAGCGGC GGAAATGGCG<br>421 AACTTTCTGG TGCTGACCAT TGGCACCGGC ATTGGCGGCG CGATTTTTTG CCAGCATCAG<br>481 CTGATTAACG GCGCGCGCTT TCGCGCGGGC GAATTTGGCT ATATGCTGAC CGATCGCCCG<br>541 GGCGGCCGCG ATCCGCGCCG CTATAGCATG AACGAAAACT GCACCCTGCG CGTGCTGCGC<br>601 CATCGCTATG CGCAGCATAT TGGCGCGCCG CTGGATAGCG TGACCGGCGA ACTGATTTTT<br>661 GATCGCTATG ATGCGGGCGA TCCGGTGTGC CAGCGCCTGG TGGCGGAATT TTTTAACGGC<br>721 CTGGGCCATG GCCTGTATAA CCTGGTGCAT ATTTTTGATC CGCAGACCAT TTTTATTGGC<br>781 GGCGGCGTGG TGGAACGCCC GGGCTTTCTG ACCCTGCTGC GCCAGCATCT GGCGTGGTTT<br>841 GGCATTGCGG ATTATCTGGA TACCGTGAGC CATGGCAACG ATGCGGGCCT GATTGGCGCG<br>901 GTGTATCATT TTAACCAGCT GTATCGCAGC CCGGATGATG ATCGCCAT
Protein sequence	1 MWSHPQFEKA GAENLYFQGM KIAAFDIGGT ALKMGVMARD GRLLETARQS INDSDGDRIL<br>61 QAMLSWLAAH PSCEGIAISA PGYIDPHSGL ITMGGAIRRF DNFAMKSWLE TRTGLPVSVE<br>121 NDANCVLLAE RWQGKAAEMA NFLVLTIGTG IGGAIFCQHQ LINGARFRAG EFGYMLTDRP<br>181 GGRDPRRYSM NENCTLRVLR HRYAQHIGAP LDSVTGELIF DRYDAGDPVC QRLVAEFFNG<br>241 LGHGLYNLVH IFDPQTIFIG GGVVERPGFL TLLRQHLAWF GIADYLDTVS HGNDAGLIGA<br>301 VYHFNQLYRS PDDDRH

<table>
<tr><td colspan="2">AtOGE (with Strep-tag and TEV restriction site)</td></tr>
<tr><td>Nucleotide sequence</td><td>1 ATGTGGAGCC ATCCGCAGTT CGAGAAGGAA AACCTGTATT TTCAGGGCAA GCTTGACGAT
61 AGCAAGACCC TGCCGATTGC GGCGCAGATG TATACCCTGC GTAACGCGGG TACCCTGGAG
121 GAACAACTGG CGATCCTGAA CCGTGCGGGC GTGAGCGCGG TTGAGACCGT GGACATGCAG
181 AAAGTTAGCG CGAGCGAGCT GAACGCGCTG CTGGAAAAGC ACAAAATCAA GGTTATTAGC
241 AGCCACGTGC CGATCGACAA ACTGCGTGGT AACCTGGATG AGGTTATTAC CGAACAAAAG
301 GCGGTGGGCA ACCCGGTGGT TACCGTTCCG TTCCTGAAGC CGGAGGATCG TCCGAAGGAT
361 GCGGCGGGTT GGACCGCGTT TGGCAAAGAA CTGGGTGGCT ACGCGGACAA GCTGAGCGCG
421 GCGGGTCTGA GCATGGCGTA TCACAACCAC GACTTCGAGA TGGTGAAATT TGATGGCAAG
481 ACCGCGCTGG AACTGCTGCT GGATGCGGCG GGTCCGAAAC TGCAAAGCGA ACTGGATGTT
541 GCGTGGGTGG CGCGTAGCGG TAACGATCCG GCGGAATTCC TGGGTACCCT GAACGGCCGT
601 GTGTTTGCGA TTCATGCGAA GGACAACGCG CCGGCGGGTA CCGCGGAGAA CGAACGTGGC
661 TTCGCGACCA TCGGTACCGG CGTTCTGGAT TGGAAAACCA TTCTGCCGGC GGCGAAGCAT
721 GCGGGTGCGC AGTGGTTCAT CCTGGAGCAC GACCTGCCGC TGGATGCGGA AGCGGTGGTT
781 ACCAAGGGCA ACGCGTTTCT GAGCGAACGT CTGCCGACCA TTCAATAA</td></tr>
<tr><td>Protein sequence</td><td>1 MWSHPQFEKE NLYFQGKLDD SKTLPIAAQM YTLRNAGTLE EQLAILNRAG VSAVETVDMQ
61 KVSASELNAL LEKHKIKVIS SHVPIDKLRG NLDEVITEQK AVGNPVVTVP FLKPEDRPKD
121 AAGWTAFGKE LGGYADKLSA AGLSMAYHNH DFEMVKFDGK TALELLLDAA GPKLQSELDV
181 AWVARSGNDP AEFLGTLNGR VFAIHAKDNA PAGTAENERG FATIGTGVLD WKTILPAAKH
241 AGAQWFILEH DLPLDAEAVV TKGNAFLSER LPTIQ</td></tr>
<tr><td colspan="2">GlvA (with Strep-tag and TEV restriction site)</td></tr>
<tr><td>Nucleotide sequence</td><td>1 ATGTGGAGCC ACCCCCAGTT CGAGAAGGCC GGCGCCATGA AGAAAAAATC GTTCTCAATC
61 GTAATAGCGG GCGGAGGGAG CACTTTCACT CCAGGGATCG TACTCATGCT CTTGGACCAT
121 TTGGAGGAGT TTCCGATCAG AAAGCTGAAG CTGTATGATA ATGATAAGGA GAGACAGGAT
181 CGAATTGCAG GCGCCTGTGA CGTTTTTATC AGAGAAAAAG CGCCGGATAT TGAATTTGCA
241 GCGACGACTG ACCCGGAAGA AGCTTTTACA GATGTCGATT TTGTTATGGC GCACATCAGA
301 GTAGGGAAAT ACGCGATGCG TGCGCTTGAT GAGCAAATTC CGTTAAAGTA CGGAGTTGTC
361 GGCCAGGAGA CGTGCGGGCC GGGCGGGATC GCATACGGTA TGCGTTCGAT CGGCGGTGTG
421 CTTGAAATAT TAGATTACAT GGAAAAATAC TCGCCTGATG CGTGGATGCT CAATTATTCC
481 AATCCGGCGG CAATTGTGGC TGAAGCTACG AGACGCCTTA GACCGAATTC TAAAATTCTC
541 AATATCTGTG ATATGCCGGT TGGGATCGAA GACCGGATGG CGCAAATTCT TGGCTTATCC
601 TCAAGAAAAG AAATGAAGGT CCGCTATTAC GGATTAAATC ACTTCGGCTG GTGGACATCA
661 ATTCAGGATC AAGAGGGCAA CGATTTAATG CCGAAGCTGA AGGAGCATGT ATCTCAATAC
721 GGCTATATTC CGAAAACAGA GGCTGAAGCG GTGGAGGCGA GCTGGAATGA CACATTCGCC
781 AAAGCGCGTG ACGTGCAGGC CGCAGATCCT GATACACTGC CGAATACGTA TTTGCAATAT
841 TATTTGTTCC CGGATGATAT GGTGAAAAAA TCAAATCCGA ATCATACGCG GGCAAATGAA
901 GTGATGGAAG GGCGCGAAGC TTTTATTTTC AGCCAATGTG ACATGATTAC ACGTGAGCAG
961 TCCACAGAAA ACAGCGAAAT CAAAATCGAT GACCACGCAT CATATATCGT TGATCTTGCC
1021 CGGGCGATTG CCTACAACAC AGGTGAAAGA ATGCTGTTGA TCGTTGAAAA TAACGGCGCA
1081 ATTGCGAACT TTGACCCGAC TGCGATGGTC GAGGTGCCAT GCATTGTCGG CTCAAATGGG
1141 CCTGAACCGA TTACCGTCGG CACCATTCCG CAATTCCAAA AAGGGCTTAT GGAGCAGCAG
1201 GTATCCGTTG AGAAGCTGAC TGTTGAAGCG TGGACGGAGA AATCGTTCCA AAAGCTGTGG
1261 CAGGCGCTAA TTTTGTCAAA AACAGTGCCG AACGCGCGTG TGGCAAGACT CATTCTTGAG
1321 GATTTAATGG AGGCCAACAA AGACTTCTGG CCTGAGCTTG ATCAAAGCCC GACCCGTATA
1381 TCATAA</td></tr>
<tr><td>Protein sequence</td><td>1 MWSHPQFEKA GAMKKKSFSI VIAGGGSTFT PGIVLMLLDH LEEFPIRKLK LYDNDKERQD
61 RIAGACDVFI REKAPDIEFA ATTDPEEAFT DVDFVMAHIR VGKYAMRALD EQIPLKYGVV
121 GQETCGPGGI AYGMRSIGGV LEILDYMEKY SPDAWMLNYS NPAAIVAEAT RRLRPNSKIL
181 NICDMPVGIE DRMAQILGLS SRKEMKVRYY GLNHFGWWTS IQDQEGNDLM PKLKEHVSQY
241 GYIPKTEAEA VEASWNDTFA KARDVQAADP DTLPNTYLQY YLFPDDMVKK SNPNHTRANE
301 VMEGREAFIF SQCDMITREQ STENSEIKID DHASYIVDLA RAIAYNTGER MLLIVENNGA
361 IANFDPTAMV EVPCIVGSNG PEPITVGTIP QFQKGLMEQQ VSVEKLTVEA WTEKSFQKLW
421 QALILSKTVP NARVARLILE DLMEANKDFW PELDQSPTRI S</td></tr>
</table>

**BglT** (with Strep-tag and TEV restriction site)	
Nucleotide sequence	1 ATGTGGAGCC ACCCCCAGTT CGAGAAGGCC GGCGCCATGC GCATTGCGGT GATTGGCGGC<br>61 GGCAGCAGCT ATACCCCGGA ACTGGTGAAA GGCCTGCTGG ATATTAGCGA AGATGTGCGC<br>121 ATTGATGAAG TGATTTTTTA TGATATTGAT GAAGAAAAAC AGAAAATTGT GGTGGATTTT<br>181 GTGAAACGCC TGGTGAAAGA TCGCTTTAAA GTGCTGATTA GCGATACCTT TGAAGGCGCG<br>241 GTGGTGGATG CGAAATATGT GATTTTTCAG TTTCGCCCGG GCGGCCTGAA AGGCCGCGAA<br>301 AACGATGAAG GCATTCCGCT GAAATATGGC CTGATTGGCC AGGAAACCAC CGGCGTGGGC<br>361 GGCTTTAGCG CGGCGCTGCG CGCGTTTCCG ATTGTGGAAG AATATGTGGA TACCGTGCGC<br>421 AAAACCAGCA ACGCGACCAT TGTGAACTTT ACCAACCCGA GCGGCCATAT TACCGAATTT<br>481 GTGCGCAACT ATCTGGAATA TGAAAAATTT ATTGGCCTGT GCAACGTGCC GATTAACTTT<br>541 ATTCGCGAAA TTGCGGAAAT GTTTAGCGCG CGCCTGGAAG ATGTGTTTCT GAAATATTAT<br>601 GGCCTGAACC ATCTGAGCTT TATTGAAAAA GTGTTTGTGA AAGGCGAAGA TGTGACCGAA<br>661 AAAGTGTTTG AAAACCTGAA ACTGAAACTG AGCAACATTC CGGATGAAGA TTTTCCGACC<br>721 TGGTTTTATG ATAGCGTGCG CCTGATTGTG AACCCGTATC TGCGCTATTA TCTGATGGAA<br>781 AAAAAAATGT TTAAAAAAAT TAGCACCCAT GAACTGCGCG CGCGCGAAGT GATGAAAATT<br>841 GAAAAAGAAC TGTTTGAAAA ATATCGCACC GCGGTGGAAA TTCCGGAAGA ACTGACCAAA<br>901 CGCGGCGGCA GCATGTATAG CACCGCGGCG GCGCATCTGA TTCGCGATCT GGAAACCGAT<br>961 GAAGGCAAAA TTCATATTGT GAACACCCGC AACAACGGCA GCATTGAAAA CCTGCCGGAT<br>1021 GATTATGTGC TGGAAATTCC GTGCTATGTG CGCAGCGGCC GCGTGCATAC CCTGAGCCAG<br>1081 GGCAAAGGCG ATCATTTTGC GCTGAGCTTT ATTCATGCGG TGAAAATGTA TGAACGCCTG<br>1141 ACCATTGAAG CGTATCTGAA ACGCAGCAAA AAACTGGCGC TGAAAGCGCT GCTGAGCCAT<br>1201 CCGCTGGGCC CGGATGTGGA AGATGCGAAA GATCTGCTGG AAGAAATTCT GGAAGCGAAC<br>1261 CGCGAATATG TGAAACTGGG CTAAC
Protein sequence	1 MWSHPQFEKA GAMRIAVIGG GSSYTPELVK GLLDISEDVR IDEVIFYDID EEKQKIVVDF<br>61 VKRLVKDRFK VLISDTFEGA VVDAKYVIFQ FRPGGLKGRE NDEGIPLKYG LIGQETTGVG<br>121 GFSAALRAFP IVEEYVDTVR KTSNATIVNF TNPSGHITEF VRNYLEYEKF IGLCNVPINF<br>181 IREIAEMFSA RLEDVFLKYY GLNHLSFIEK VFVKGEDVTE KVFENLKLKL SNIPDEDFPT<br>241 WFYDSVRLIV NPYLRYYLME KKMFKKISTH ELRAREVMKI EKELFEKYRT AVEIPEELTK<br>301 RGGSMYSTAA AHLIRDLETD EGKIHIVNTR NNGSIENLPD DYVLEIPCYV RSGRVHTLSQ<br>361 GKGDHFALSF IHAVKMYERL TIEAYLKRSK KLALKALLSH PLGPDVEDAK DLLEEILEAN<br>421 REYVKLG
***At*HYD** (with Strep-tag and TEV restriction site)	
Nucleotide sequence	1 ATGTGGAGCC ACCCGCAGTT CGAGAAGGAA AACCTGTACT TTCAGGGTAT GAAGACCATC<br>61 AAAGGCCCGG CGATTTTCCT GGCGCAGTTT GTGGGTGATA AAGCGCCGTT CGACACCCTG<br>121 GATAACCTGG GCCAGTGGGC GGCGAGCCTG GGTTACAAGG GCATCCAAGT TCCGACCGAT<br>181 CCGAAACTGT TTGACCTGGA GAAGGCGGCG GCGAGCAAAG CGTATTGCGA CGATATCAAG<br>241 GGTCGTCTGG CGGAAACCGG CATCGAGATT ACCGAACTGA GCACCCACAT TCAGGGTCAA<br>301 CTGGTGAGCG TTCACCCGGC GTACGATGAG ATGTTCGATG GTTTTGCGCC GGCGGAACTG<br>361 CGTGGTCGTC CGCAGGCGCG TCAAGAGTGG GCGGTGAACC AGCTGAAGTG CGCGGCGAAA<br>421 GCGAGCCAAC ACCTGGGTCT GAAGAGCCAT GCGAGCTTCA GCGGTGCGCT GGCGTGGCCG<br>481 TTTATCTATC CGTGGCCGCA GCGTCCGGCG GGTCTGGTGG AGATGGCGTT TGCGGAACTG<br>541 GGCAAACGTT GGACCCCGAT TCTGGATACC TTTGAGGAAA ACGGTGTTGA CCTGTGCTAC<br>601 GAGCTGCACC CGGGTGAAGA CCTGCACGAT GGCATCACCT TCGAGCGTTT TCTGGAAGCG<br>661 ACCGGCAACC ACAGCCGTGC GAACATTCTG TATGACCCGA GCCACTTCGT GCTGCAAGCG<br>721 ATGGACTACC TGGATTTTAT CGACATTTAT CACGAGCGTA TCCGTGCGTT CCACGTTAAG<br>781 GATGCGGAAT TTAACCCGAC CGGTCGTAGC GGCGTGTATG GTGGCTATCA GGGTTGGGTT<br>841 GACCGTCCGG GCCGTTTCCG TAGCCTGGGT GATGGCCACG TGGACTTCGG TGCGGTTTTT<br>901 AGCAAGCTGA CCCAATATGA TTTTGAAGGT TGGGCGGTGC TGGAGTGGGA ATGCGCGCTG<br>961 AAACATCCGG AGGATGGTGC GCGTGAAGGC GCGGGCTTCA TTGAGAACCA CATCATTCGT<br>1021 GTTACCGAAC GTGCGTTCGA CGATTTTGCG AAAAGCGGTA CCGACGATGC GGCGAACCGT<br>1081 CGTCTGCTGG GCCTGTAA
Protein sequence	1 MWSHPQFEKE NLYFQGMKTI KGPAIFLAQF VGDKAPFDTL DNLGQWAASL GYKGIQVPTD<br>61 PKLFDLEKAA ASKAYCDDIK GRLAETGIEI TELSTHIQGQ LVSVHPAYDE MFDGFAPAEL<br>121 RGRPQARQEW AVNQLKCAAK ASQHLGLKSH ASFSGALAWP FIYPWPQRPA GLVEMAFAEL<br>181 GKRWTPILDT FEENGVDLCY ELHPGEDLHD GITFERFLEA TGNHSRANIL YDPSHFVLQA<br>241 MDYLDFIDIY HERIRAFHVK DAEFNPTGRS GVYGGYQGWV DRPGRFRSLG DGHVDFGAVF<br>301 SKLTQYDFEG WAVLEWECAL KHPEDGAREG AGFIENHIIR VTERAFDDFA KSGTDDAANR<br>361 RLLGL

Pu**CGE**$^{\alpha}$ (with Strep-tag and TEV restriction site)	
Nucleotide sequence	1 ATGGGCTGGA GCCATCCGCA GTTCGAGAAG GAAAACCTGT ATTTTCAGGG CAAGCTTATG<br>61 AGCAACGTGA AACTGGGTGT TACCCTGTAT AGCTTCAGCA CCGAGTACTG CCAGGGCAAA<br>121 ATGACCCTGG AAGACTGCAT TCGTACCGCG AAGGAGCTGG GTGCGGCGGG TTTCGAAATC<br>181 GTGGCGACCC AGATGATTCC GAGCTACCCG TATGTTAGCG ACAAATTTCT GGGCGAGCTG<br>241 AAGAGCATTT GCCAATACTA TGATATGGAA CCGGTGTGCT ACGGTGCGAA CTGCGACCGT<br>301 GGTCTGCGTG GCGATCGTAA CCTGACCGGC GACGAAATGG TGGCGATGGC GGTTCGTGAT<br>361 ATCAAGAACG CGCACAAAAT GGGTTGCAAG GTGGTTCGTG AGCAGTGGCT GATGGGCCCG<br>421 GAAAACTTCG CGAAACTGGC GCCGTTTGCG GAGCACTACG GTGTGAAGGT TGGCATCGAG<br>481 GTTCACAACC CGGAAACCCC GATTACCCAA AGCACCAAGG ATTATATCGC GGCGATTGAC<br>541 AAAACCGGTA GCAAGTACCT GGGCCTGATT CCGGACTTCG GTTGCTTTGC GAACAAGCCG<br>601 AACAAAATGA ACTGGGATAA CGCGCTGGCG GATGGTGCGG ATAAGAAACT GCTGGAGATG<br>661 GCGCGTGACA TGAAATATGA TAACGTGCCG TACGACGAAG CGGTTAAGCG TCTGACCGCG<br>721 GCGGGTGCGA AGAAAGTGGA GCTGACCACC ATGCGTGATA TGTATACCTT CCTGACCTTT<br>781 AAGAAAGACG TTAGCGCGGA ACTGCAGGGT CTGAAAGATA TGATCCCGTA CTGCATTCAC<br>841 ATGCACGGCA AGTACCACTA TATGTACGAG AACCTGCAAG AAGCGGCGAT CCCGTATGAC<br>901 GATATCATGA AAATTGTGAG CGAGAGCGAC TATGATGGTT ACATCGTTAG CGAATATGAG<br>961 GAATACAACA GCGGCCACAG CATTGAGATG CTGCGTCGTC ACCTGAAGAT GATGCACAAC<br>1021 TTTGTGGATT AA
Protein sequence	1 MGWSHPQFEK ENLYFQGKLM SNVKLGVTLY SFSTEYCQGK MTLEDCIRTA KELGAAGFEI<br>61 VATQMIPSYP YVSDKFLGEL KSICQYYDME PVCYGANCDR GLRGDRNLTG DEMVAMAVRD<br>121 IKNAHKMGCK VVREQWLMGP ENFAKLAPFA EHYGVKVGIE VHNPETPITQ STKDYIAAID<br>181 KTGSKYLGLI PDFGCFANKP NKMNWDNALA DGADKKLLEM ARDMKYDNVP YDEAVKRLTA<br>241 AGAKKVELTT MRDMYTFLTF KKDVSAELQG LKDMIPYCIH MHGKYHYMYE NLQEAAIPYD<br>301 DIMKIVSESD YDGYIVSEYE EYNSGHSIEM LRRHLKMMHN FVD
Pu**CGE**$^{\beta}$ (with Strep-tag and TEV restriction site)	
Nucleotide sequence	1 ATGTGGAGCC ATCCGCAGTT CGAGAAGGAA AACCTGTATT TTCAGGGCAG ATCTATGGGT<br>61 CTGGCGCTGC GTCTGAACTT TGTGGACGTG GTTTGCGACG ATAGCCTGAA GAACTTCTGG<br>121 GCGAACGGTA AGAAAATCGG CTACCAGTTT GACGTTCGTC TGAGCTACTA TCGTGGCCAC<br>181 TTCCTGAGCA CCATCGATGA AATTGGTGTG AAGGTTGACG GCGTGGATGT TCCGGCGGAG<br>241 AACATCAGCC TGTGCCTGGA CGGTAAAGAA TATGGCGTGG CGGAGCTGCA CGATCTGGTG<br>301 AACGTGTTCT GGCCGATCAT TGAACCGGCG ACCATTAAGG TGTTCCAACC GGGTGGCCTG<br>361 AGCGAGGAAG AGCACGACGT TGATTTCACC CTGTACTTTC GTAGCCCGTA TATGGCGCTG<br>421 AGCGAAACCG AGTACCAGAG CATTGATAGC TGCGGTAGCA AACGTCTGAA CGTTCAAAAC<br>481 TAA
Protein sequence	1 MWSHPQFEKE NLYFQGRSMG LALRLNFVDV VCDDSLKNFW ANGKKIGYQF DVRLSYYRGH<br>61 FLSTIDEIGV KVDGVDVPAE NISLCLDGKE YGVAELHDLV NVFWPIIEPA TIKVFQPGGL<br>121 SEEEHDVDFT LYFRSPYMAL SETEYQSIDS CGSKRLNVQN
**Gfo Oxo** from *A. tumefaciens* (with Strep-tag and TEV restriction site)	
Nucleotide sequence	1 ATGTGGAGCC ATCCGCAGTT CGAGAAGGAA AACCTGTATT TTCAGGGCAT GAGCAGCGCG<br>61 ACCGCGCGTT TCAACAGCCG TCGTATCCGT CTGGGTATGG TGGGTGGCGG TCAGGGTGCG<br>121 TTTATCGGCG CGGTTCACCG TATTGCGGCG CGTCTGGACG ATCGTTACGA GCTGGTTGCG<br>181 GGTGCGCTGA GCAGCGACCC GGCGCGTGCG AGCGTTAGCG CGACCCTGCT GGGTATTGCG<br>241 CCGGAACGTA GCTATGCGAG CTTTGAGGAA ATGGCGAGCG CGGAGGCGGG TCGTGAAGAT<br>301 GGCATCGAGG CGGTGGCGAT TGTTACCCCG AACCACCTGC ACTTTGCGCC GAGCAAGTTC<br>361 TTTCTGGAGA GCGGCATCCA CGTGATTTGC GACAAACCGG TTACCGCGAC CCTGGAGGAA<br>421 GCGAAGGAAC TGGCGAAAAT CGTGCGTGCG AGCGATCGTC TGTTCATTCT GACCCACAAC<br>481 TACACCGGTT ATGCGATGCT GCGTCAAATG CGTGAGATGG TGGCGAACGG TGCGATCGGC<br>541 AAGCTGCGTC ACGTTCAGGC GGAATACGCG CAAGACTGGC TGACCGAGGC GGTTGAAAAG<br>601 ACCGGTGCGA AGGGTGCGGA GTGGCGTACC GACCCGAGCC GTAGCGGTGC GGGCGGTGCG<br>661 ATCGGTGATA TTGGCACCCA CGCGTTCAAC GCGGCGGCGT TTGTGACCGG CGAAATTCCG<br>721 GCGAGCCTGT ACGCGGATCT GACCAGCTTC GTTCCGGGTC GTCAGCTGGA CGATAGCGCG<br>781 AACATCCTGC TGCGTTATGA GAGCGGTGCG AAAGGCATGC TGTGGGCGAG CCAAATTGCG<br>841 GTGGGCAACG AAAACGCGCT GAGCCTGCGT GTTTACGGTG ACAAGGGCGG TCTGGAATGG<br>901 CACCACCGTG TGCCGGATGA GCTGTGGTTT ACCCCGTATG GCGAACCGAA ACGTCTGATC<br>961 ACCCGTAACG GTGCGGGTGC GGGTGCGGCG GCGAACCGTG TGAGCCGTGT TCCGAGCGGT<br>1021 CACCCGGAAG GCTACCTGGA GGGTTTCGCG ACCATTTATC GTGAGGCGGC GGACGCGATC<br>1081 ATTGCGAAGC GTGAAGGCAA AGCGGCGGCG GGTGACGTTA TCTATCCGGG TATTGAGGAT<br>1141 GGTCTGGCGG GCCTGGCGTT TATTGATGCG GCGGTGCGTA GCAGCCTGAC CAGCAGCTGG<br>1201 GTTGAAATCG ATATTTAA
Protein sequence	1 MWSHPQFEKE NLYFQGMSSA TARFNSRRIR LGMVGGGQGA FIGAVHRIAA RLDDRYELVA<br>61 GALSSDPARA SVSATLLGIA PERSYASFEE MASAEAGRED GIEAVAIVTP NHLHFAPSKF<br>121 FLESGIHVIC DKPVTATLEE AKELAKIVRA SDRLFILTHN YTGYAMLRQM REMVANGAIG<br>181 KLRHVQAEYA QDWLTEAVEK TGAKGAEWRT DPSRSGAGGA IGDIGTHAFN AAAFVTGEIP<br>241 ASLYADLTSF VPGRQLDDSA NILLRYESGA KGMLWASQIA VGNENALSLR VYGDKGGLEW<br>301 HHRVPDELWF TPYGEPKRLI TRNGAGAGAA ANRVSRVPSG HPEGYLEGFA TIYREAADAI<br>361 IAKREGKAAA GDVIYPGIED GLAGLAFIDA AVRSSLTSSW VEIDI

Supplementary Table 7. Oligonucleotide primers used in this study. Homologous overhangs are in italic.

**Primers used for sub-cloning of *Pu*CGE**	
DgpB-del1-fwd	*GTACACGGCCG*GGAACTATATCCGGATTGGCGAATGG
DgpB-del1-rev	*CCGGATATAGTTCC*CGGCCGTGTACAATACGATTACTTTCTG

Supplementary References

1. Koshland Jr., D. E. Stereochemistry and the mechanism of enzymatic reactions. *Biological Reviews* **28**, 416–436 (1953).
2. Zechel, D. L. & Withers, S. G. Glycosidase mechanisms: anatomy of a finely tuned catalyst. *Acc Chem Res* **33**, 11–8 (2000).
3. Thompson, J. *et al.* The Gene *glvA* of *Bacillus subtilis* 168 encodes a metal-requiring, NAD(H)-dependent 6-phospho-α-glucosidase. *Journal of Biological Chemistry* **273**, 27347–27356 (1998).
4. Rajan, S. S. *et al.* Novel catalytic mechanism of glycoside hydrolysis based on the structure of an NAD^+/Mn^{2+}-dependent phospho-α-glucosidase from *Bacillus subtilis*. *Structure* **12**, 1619–1629 (2004).
5. Yip, V. L. Y., Thompson, J. & Withers, S. G. Mechanism of GlvA from *Bacillus subtilis*: A detailed kinetic analysis of a 6-phospho-α-glucosidase from glycoside hydrolase family 4. *Biochemistry* **46**, 9840–9852 (2007).
6. Sannikova, N. *et al.* Both Chemical and non-chemical steps limit the catalytic efficiency of family 4 glycoside hydrolases. *Biochemistry* **57**, 3378–3386 (2018).
7. Nakamura, K., Zhu, S., Komatsu, K., Hattori, M. & Iwashima, M. Deglycosylation of the Isoflavone *C*-glucoside puerarin by a combination of two recombinant bacterial enzymes and 3-oxo-glucose. *Appl Environ Microbiol* **86**, e00607-20 (2020).
8. Mori, T. *et al.* *C*-Glycoside metabolism in the gut and in nature: Identification, characterization, structural analyses and distribution of C-C bond-cleaving enzymes. *Nat Commun* **12**, 6294 (2021).
9. Kim, E. M. *et al.* Identification and characterization of the *Rhizobium* sp. strain GIN611 glycoside oxidoreductase resulting in the deglycosylation of ginsenosides. *Appl Environ Microbiol* **78**, 242–249 (2012).
10. Kuritani, Y. *et al.* Conversion of levoglucosan into glucose by the coordination of four enzymes through oxidation, elimination, hydration, and reduction. *Sci Rep* **10**, 20066 (2020).
11. Thompson, J., Lichtenthaler, F. W., Peters, S. & Pikis, A. β-glucoside kinase (BglK) from *Klebsiella pneumoniae*. Purification, properties, and preparative synthesis of 6-phospho-β-D-glucosides. *Journal of Biological Chemistry* **277**, 34310–34321 (2002).
12. Yip, V. L. Y. *et al.* An unusual mechanism of glycoside hydrolysis involving redox and elimination steps by a family 4 β-glycosidase from *Thermotoga maritima*. *J Am Chem Soc* **126**, 8354–8355 (2004).
13. Tegl, G. & Nidetzky, B. Leloir glycosyltransferases of natural product *C*-glycosylation: structure, mechanism and specificity. *Biochem Soc Trans* **48**, 1583–1598 (2020).
14. Tanner, M. E. Mechanistic studies on the indole prenyltransferases. *Nat Prod Rep* **32**, 88–101 (2015).
